# Formosins A–F: Diterpenoids with Anti-microbial Activities from *Excoecaria formosana*

**DOI:** 10.1007/s13659-016-0086-6

**Published:** 2016-01-27

**Authors:** Bing-Dong Lin, Bin Zhou, Lei Dong, Yan Wu, Jian-Min Yue

**Affiliations:** State Key Laboratory of Drug Research, Shanghai Institute of Materia Medica, Chinese Academy of Sciences, 555 Zuchongzhi Road, Shanghai, 201203 People’s Republic of China

**Keywords:** *Excoecaria formosana*, Halimane-type, Clerodane-type, Diterpenoid, Anti-microbial

## Abstract

**Abstract:**

Three new halimane-type diterpenoids formosins A–C (**1**–**3**), and three clerodane-type diterpenoids formosins D–F (**4**–**6**), were isolated from the twigs of *Excoecaria formosana*. Their structures were assigned on the basis of spectroscopic data analysis. Compounds **1** and **4** showed moderate anti-microbial activities against *Bacillus subtilis* (MIC = 50 and 50 μg/mL, respectively). Compound **6** exhibited moderate anti-microbial activities against two strains of *Helicobacter pylori* (*Hp*-SS1 and ATCC 43504) with MIC values of 50 and 50 μg/mL, respectively.

**Graphical abstract:**

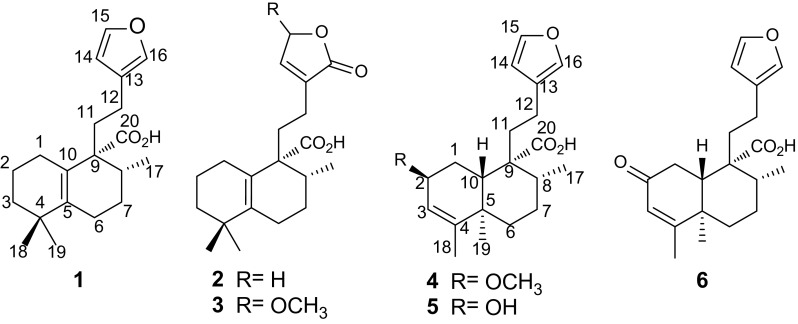

**Electronic supplementary material:**

The online version of this article (doi:10.1007/s13659-016-0086-6) contains supplementary material, which is available to authorized users.

## Introduction

The genus *Excoecaria* (Euphorbiaceae) compromising 40 species, are widely distributed in Africa and East Asia [1]. Several plants in this genus have been used in folk medicine to treat psoriasis, dermatitis and pruritus [2–4]. The characteristic of this plant genus is the poisonous milk latex, which causes skin blister [5]. Chemical investigations on this plant genus have led to the isolation of structurally diverse compounds with significant biological activities including anti-tumor promoting, anti-ulcer, and anti-microbial activities [6–10]. In the current study, three new halimane-type diterpenoids formosins A–C (**1**–**3**), and three clerodane-type diterpenoids formosins D–F (**4**–**6**), were isolated from the twigs of *Excoecaria formosana*. Presented herein are the isolation, structural characterization, and biological evaluation of these compounds.

## Results and Discussion

**Fig. 1:**
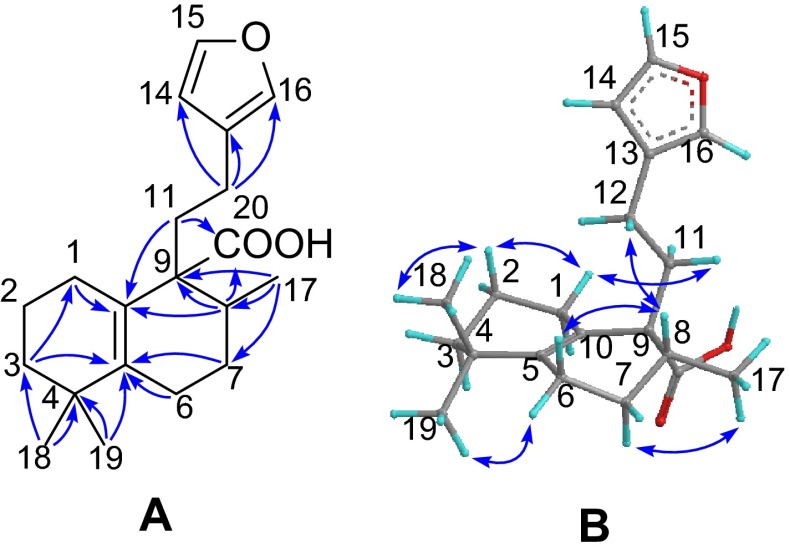
**a** Selected HMBC, and **b** ROESY correlations of **1**

Compound **1**, a white powder, gave a molecular formula C_20_H_28_O_3_ as determined by the (+)-HRESIMS ion at *m*/*z* 339.1937 [M + Na]^+^ (calcd 339.1931) requiring seven degrees of unsaturation. The IR absorptions (3000–2800 cm^−1^, broad band) and (1695 cm^−1^) showed the presence of a carboxylic group. The diagnostic NMR data (Tables 1, 2) suggested the presence of a *β*-substituted furan ring (*δ*_H_ 6.27, 7.22, and 7.34), a persubstituted double bond, and a carboxylic group (*δ*_C_ 181.5). These functionalities accounted for five out of the seven indices of hydrogen deficiency, requiring the presence of two additional rings in the structure of **1**. The aforementioned data suggested that compound **1** is a halimane-type diterpenoid and is structurally related to crotohalimaneic acid [11]. The planar structure of **1** was deduced by 2D NMR spectra. In the HMBC spectrum (Fig. 1), two tertiary methyls (*δ*_H_ 1.00, and 1.04, each 3H, s) correlating with C-3, C-4 and C-5 were attached to C-4. The multiple HMBC correlations of H-3, H-7, and H_3_-19/C-5 (*δ*_C_ 141.3); H-1, H-8, and H-11/C-10 (*δ*_C_ 125.2); and H_3_-17/C-7, C-8, and C-9 indicated the presence of a typical Δ^5(10)^ double bond and 8-Me (C-17). The *β*-substituted furan ring and carboxylic group were located at C-12 and C-9 by the HMBC correlations of H-12/C-13, C-14 and C-16; and H-8 and H-11/C-20, respectively. The relative configuration of **1** was established by the ROESY experiment (Fig. 1). The ROESY cross-peaks of H_3_-18/H-2*β*, H-2*β*/H-1*β*, H-1*β*/H-11, and H-12/H-8 indicated that they are co-facial and randomly assigned in a *β*-configuration. In consequence, H_3_-19 and H_3_-17 were *α*-oriented by the ROESY correlations of H_3_-19/H-6*α* and H_3_-17/H-7*α*. The structure of **1** (formosin A) was herein elucidated as shown.

**Table 1:** ^1^H NMR data for compounds **1**–**6** in CDCl_3_ at 400 MHz

Position	**1** (mult., *J* in Hz)	**2** (mult., *J* in Hz)	**3** (mult., *J* in Hz)	**4** (mult., *J* in Hz)	**5** (mult., *J* in Hz)	**6** (mult., *J* in Hz)
1	*α* 1.92 (m)	*α* 1.86 (m)	*α* 1.90 (m)	*α* 1.76 (m)	*α* 1.67 (m)	*α* 2.89 (dd, 17.9, 14.5)
	*β* 1.68 (m)	*β* 1.64 (m)	*β* 1.68 (m)	*β* 2.22 (m)	*β* 2.32 (m)	*β* 2.66 (dd, 17.9, 3.1)
2	1.64 (m)	1.60 (m)	1.60 (m)	3.61 (m)	4.19 (m)	
3	*α* 1.36 (td, 12.5, 5.4)	*α* 1.35 (td, 12.1, 4.3)	*α* 1.36 (m)	5.43 (d, 4.7)	5.27 (br s)	5.78 (s)
	*β* 1.50 (m)	*β* 1.46 (m)	*β* 1.52 (m)			
6	*α* 2.12 (m)	*α* 2.12 (m)	*α* 2.16 (m)	*α* 1.79 (m)	*α* 1.80 (m)	*α* 2.16 (m)
	*β* 1.94 (m)	*β* 1.92 (m)	*β* 1.94 (m)	*β* 1.29 (m)	*β* 1.21 (m)	*β* 1.40 (m)
7	*α* 1.52 (m)	*α* 1.52 (m)	*α* 1.56 (m)	*α* 2.11 (m)	*α* 2.11 (m)	*α* 2.24 (m)
	*β* 1.70 (m)	*β* 1.66 (m)	*β* 1.72 (m)	*β* 1.47 (m)	*β* 1.47 (m)	*β* 1.56 (m)
8	1.78 (m)	1.73 (m)	1.75 (m)	1.61 (m)	1.55 (m)	1.62 (m)
10				1.95 (m)	1.62 (m)	1.90 (m)
11	1.98 (m)	1.98 (m)	2.00 (m)	1.94 (m) 2.23 (m)	1.92 (m) 2.25 (m)	1.94 (m) 2.18 (m)
12	2.10 (m) 2.27 (m)	2.02 (m) 2.15 (m)	2.03 (m) 2.18 (m)	2.32 (m) 2.57 (m)	2.34 (m)	2.33 (m)
14	6.27 (br s)	7.17 (t, 1.6)	6.83 (br s)	6.28 (br s)	6.28 (br s)	6.26 (br s)
15	7.34 (br s)	4.77 (d, 1.8)	5.74 (br s)	7.34 (br s)	7.35 (br s)	7.34 (br s)
16	7.22 (br s)			7.22 (br s)	7.23 (br s)	7.22 (br s)
17	0.95 (d, 6.7)	0.94 (d, 6.7)	0.94 (d, 5.6)	1.16 (d, 6.8)	1.15 (d, 6.9)	1.17 (d, 6.8)
18	1.04 (s)	1.04 (s)	1.03 (s)	1.65 (s)	1.62 (s)	1.90 (s)
19	1.00 (s)	1.00 (s)	0.99 (s)	0.91 (s)	0.99 (s)	1.08 (s)
2-OMe				3.34 (s)		
15-OMe			3.49 (s)

**Table 2:** ^13^C NMR data for compounds **1**–**6** in CDCl_3_ at 100 MHz

Carbons	**1**	**2**	**3**	**4**	**5**	**6**
1	19.5	19.4	19.4	24.6	30.7	36.5
2	28.2	28.0	28.1	74.3	69.4	199.9
3	39.4	39.3	39.3	120.6	125.1	125.8
4	34.8	34.8	34.8	149.2	146.5	171.4
5	141.3	141.7	142.2	39.3	39.2	40.2
6	25.1	25.0	25.0	36.8	37.1	36.2
7	27.5	27.4	27.4	27.1	27.0	26.7
8	33.6	33.4	33.5	36.8	36.9	36.7
9	54.9	54.6	54.6	49.5	49.4	49.8
10	125.2	125.3	125.1	42.5	46.1	46.1
11	31.0	28.7	28.4 (28.5)	33.7	33.7	33.4
12	18.8	19.5	19.5	17.2	17.4	17.8
13	125.7	134.5	138.9	124.8	124.3	123.9
14	111.0	143.9	141.5	110.9	110.8	110.7
15	142.7	70.2	102.5	142.8	142.9	142.9
16	138.5	174.3	171.3	138.5	138.6	138.7
17	17.4	17.4	17.4	16.5	16.6	16.3
18	26.6	26.6	26.6	18.1	17.9	19.2
19	29.0	28.9	28.9	16.0	17.4	16.0
20	181.5	180.4	180.0	182.6	181.8	181.3
2-OMe				56.3		
15-OMe			56.9 (57.0)

Compound **2** had the molecular formula of C_20_H_28_O_4_, as determined by the ^13^C NMR data and the (+)-HRESIMS ion at *m*/*z* 355.1839 [M + Na]^+^ (calcd 355.1880), which is 16 mass units more than that of **1**. Comparison of its NMR spectroscopic data (Tables 1, 2) with those of **1** revealed they are structural analogues with the obvious difference being the presence of an *α*,*β*-unsaturated-*γ*-lactone moiety instead of the *β*-substituted furan ring. It was confirmed by the NMR data (*δ*_H_ 4.77 and 7.17; *δ*_C_ 70.2, 134.5, 143.9, and 174.3), as well as the key HMBC correlations from H-12 to C-13, C-14, and C-16 (Figure S13, Supporting Information). Thus, the structure of **2** (formosin B) was determined as shown.

Compound **3** displayed a molecular formula of C_21_H_30_O_5_ as established by the (+)-HRESIMS at *m/z* 385.1991 [M + Na]^+^ (calcd 385.1991) and the ^13^C NMR data. Analysis of the NMR data (Tables 1, 2) of **3** showed many similarities to those of **2.** The only difference was the presence of an additional methoxy group (*δ*_H_ 3.49, s, 3H), which was located at C-15 to form the acetal motif, which was confirmed by the downfield shifted C-15 (Δ*δ*_*C*_ 32.3) as compared to that of **2**. Compound **3** was obtained as a pair of inseparable C-15 epimers, which exhibited several pairs of very close carbon resonances in the ^13^C NMR spectrum (Figure S19, Supplementary Material). Therefore, the structure of **3** (formosin C) with its relative configuration was confirmed as depicted by the HMBC and ROESY spectra (Figures S21 and S22, Supplementary Material).

**Fig. 2:**
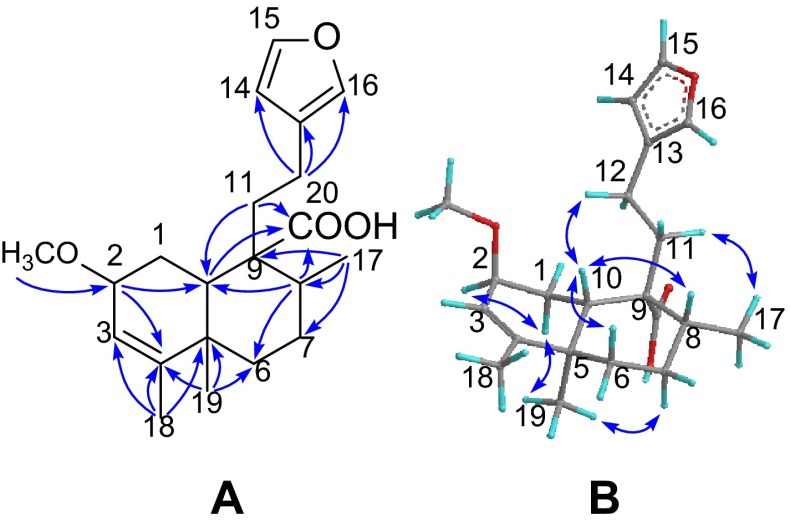
**a** Selected HMBC, and **b** ROESY correlations of **4**

Compound **4** was obtained as a white powder with a molecular formula of C_21_H_30_O_4_ as established by the ^13^C NMR data and the (+)-HRESIMS ion at *m/z* 369.1970 [M + Na]^+^ (calcd 369.2036), demanding seven degrees of unsaturation. The IR absorption bands (3000–2800 cm^−1^, broad band; and 1695 cm^−1^) showed the presence of a carboxylic group. The characteristic NMR signals for a *β*-furan ring, a trisubstituted double bond, a methoxyl and a carboxylic groups were observed from the ^1^H and ^13^C NMR spectroscopic data analysis (Tables 1, 2). Comprehensive analysis of the NMR spectra of **4** revealed its structure is highly related with that of junceic acid [12] with a clerodane-type diterpenoid backbone. The only difference was the presence of an additional methoxyl group in **4**, which was placed at C-2 by the HMBC correlation (Fig. 2) from CH_3_O (*δ*_H_ 3.34) to C-2 (*δ*_C_ 74.3). The carboxylic group was attached to C-9 via the HMBC correlations from H-8, H-10 and H-11 to C-20 (*δ*_C_ 182.6). The key HMBC cross-peaks from H_3_-18 to C-3 (*δ*_C_ 120.6), C-4 (*δ*_C_ 149.2) and C-5, and from H_3_-19 to C-4 revealed the presence of Δ^3^ double bond. The relative configuration of **4** was established by the ROESY experiment (Fig. 2). The ROESY correlations of H-10/H-6*β* and H-10/H-8 indicated that H-8 and H-10 are co-facial and randomly assigned in a *β*-configuration. Consequently, H_3_-19 and H-2 were thus assigned to be *α*-directed by the ROESY correlations of H_3_-19/H-1*α*, H_3_-19/H-7*α*, and H-2/H-1*α.* Therefore, the structure of **4** (formosin D) was established as depicted.

Compound **5** possessed a molecular formula C_20_H_28_O_4_ based on the ^13^C NMR data and the (+)-HRESIMS ion at *m/z* 355.1885 [M + Na]^+^ (calcd 355.1888), which is 14 mass units less than that of **4**. Detailed analysis of the NMR data (Tables 1, 2) of **5** revealed that its structure is closely related with that of **4** with the only difference being the absence of the methyl etherification, which is consistent with the molecular formula. The structure of **5** (formosin E) with the relative configuration was further confirmed by HMBC and ROESY spectra (Figures S39 and S40, Supplementary Material).

Compound **6**, named formosin F, exhibited a sodiated molecular ion at *m/z* 353.1724 [M + Na]^+^ (calcd 353.1723) in the (+)-HRESIMS, consistent with a molecular formula of C_20_H_26_O_4_, which was supported by the ^13^C NMR data. Comparison of the NMR data (Tables 1, 2) of **6** with those of **5** revealed they are structural analogues. The main difference was the presence of a keto group at C-2 in **6** instead of the hydroxy group in the latter, which was confirmed by the HMBC correlations (Figure S42, Supporting Information) from H-3 and H-10 to C-2 (*δ*_C_ 199.9). The structure of **6** was thus determined as shown.

The new isolates were tested for anti-microbial activities against a panel of microbes *in vitro* by the microdilution method [13, 14]. Compounds **1** and **4** exhibited moderate activity against *Bacillus subtilis ATCC 6633* with MIC values of 50 and 50 μg/mL, respectively, where magnolol was used as the positive control (MIC = 12.5 μg/mL). Compound **6** showed moderate antibacterial activities against two strains *Helicobacter pylori* (*Hp*-SS1 or ATCC 43504) with MIC values of 50 and 50 μg/mL, respectively, and metronidazole was used as the positive control (MIC = 0.312 and 128 μg/mL, respectively).

## Experimental Section

### General Experimental Procedures

Optical rotations were determined on a Perkin-Elmer 341 polarimeter. UV spectra were recorded on a Shimadzu UV-2550 spectrophotometer. IR spectra were acquired on a Perkin-Elmer 577 spectrometer. NMR spectra were measured on a Bruker AM-400 spectrometer with TMS as internal standard. ESIMS and HRESIMS were performed on a Bruker Daltonics Esquire 3000 plus and a Waters-Micromass Q-TQF Ultima Global mass spectrometer, respectively. Semi-preparative HPLC was performed on a Waters 1525 binary pump system with a Waters 2489 detector (210 nm) and equipped with a YMC-Pack ODS-A (250 × 10 mm, S-5 μm). Silica gel (200–300 mesh, Qingdao Haiyang Chemical Co., Ltd.), C_18_ reversed-phase (RP-18) silica gel (20–45 μm, Fuji Silysia Chemical Ltd.), CHP20P MCI gel (75–150 μm, Mitsubishi Chemical Corporation), and Sephadex LH-20 gel (Amersham Biosciences) were used for column chromatography (CC). Pre-coated silica gel GF_254_ plates (Qingdao Haiyang Chemical Co., Ltd.) were used for TLC detection. All solvents used for CC were of analytical grade (Shanghai Chemical Reagents Co., Ltd.), and solvents used for HPLC were of HPLC grade (J & K Scientific Ltd.).

### Plant Material

The twigs of *E. formosana* were collected from Sanya city of Hainan Province, the People’s Republic of China, and authenticated by Prof. S.-M. Huang, Department of Biology, Hainan University. A voucher specimen has been deposited in Shanghai Institute of Materia Medica, Chinese Academy of Sciences (accession number: SMEF-2006-1Y).

### Extraction and Isolation

The air-dried, powdered twigs of *E. formosana* (6.0 kg) was extracted three times with 95 % EtOH at room temperature to give a crude extract (290 g), which was partitioned between EtOAc and H_2_O. The EtOAc-soluble fraction (85 g) was subjected to passage over an MCI gel column (MeOH/H_2_O, 3:7–9:1) to afford fractions A–G. Fraction C (25.7 g) was separated over a silica gel column eluted with gradient mixtures of petroleum ether–acetone (35:1–1:1, v/v) to afford major fractions C1-C6. Fraction C3 (3.4 g) was separated on a reversed-phase C_18_ silica gel column (MeOH/H_2_O, 55–100 %) to yield three major portions (C3a–C3c), and each of those was purified by a semi-preparative HPLC (60 % CH_3_CN in H_2_O, 3 mL/min) to yield compounds **1** (20 mg), **2** (10 mg), and **6** (100 mg), respectively. Fraction C4 (515 mg) was purified by a semi-preparative HPLC (55 % CH_3_CN in H_2_O, 3 mL/min) to give compound **4** (15 mg). Fraction C6 (1.5 g) was separated on a column of Sephadex LH-20, and then purified by a semi-preparative HPLC (50 % CH_3_CN in H_2_O, 3 mL/min) to yield compound **5** (8 mg). Fraction E (11.4 g) was chromatographed on a silica gel column eluted with petroleum ether-ethyl acetate (25:1–1:4, v/v) to afford subfractions E1–E4. Fraction E2 (217 mg) was separated on a reversed-phase column containing C_18_ silica gel (MeOH/H_2_O, 70–100%) to yield three fractions E2a–E2c. Fraction E2b (35 mg) was separated by a semi-preparative HPLC (70 % CH_3_CN in H_2_O, 3 mL/min) to yield compound **3** (8 mg).

### Formosin A (**1**)

White powder; $[\alpha]_{\rm D}^{21}$ +123 (*c* 0.10, MeOH); UV (MeOH) *λ*_max_ (log *ε*) 206 (4.19) nm; IR (KBr) ν_max_ 3444, 3000–2500, 1695, 1622, 1458, 1257, 1024, 600 cm^−1^; ^1^H NMR (CDCl_3_), see Table 1 and ^13^C NMR (CDCl_3_) see Table 2; (+)-ESIMS *m/z* 339.2 [M + Na]^+^; (+)-HRESIMS *m*/*z* 339.1937 [M + Na]^+^ (calcd for C_20_H_28_O_3_Na, 339.1931).

### Formosin B (**2**)

White powder; $[\alpha ]_{\text{D}}^{21}$ +212 (*c* 0.10, MeOH); UV (MeOH) *λ*_max_ (log *ε*) 207 (3.92); IR (KBr) *ν*_max_ 3435, 3000–2500, 1753, 1695, 1456, 1205, 1070, 756 cm^−1^; ^1^H NMR (CDCl_3_), see Table 1 and ^13^C NMR (CDCl_3_) see Table 2; (+)-ESIMS *m*/*z* 355.2 [M + Na]^+^; (+)-HRESIMS *m*/*z* 355.1839 [M + Na]^+^ (calcd for C_20_H_28_O_4_Na, 355.1880).

### Formosin C (**3**)

White powder; $[\alpha ]_{\text{D}}^{21}$ +22 (*c* 0.10, MeOH); UV (MeOH) *λ*_max_ (log *ε*) 211 (3.58); IR (KBr) *ν*_max_ 3425, 3000–2500, 1726, 1693, 1659, 1468, 1178, 1065, 600 cm^−1^; ^1^H NMR (CDCl_3_), see Table 1 and ^13^C NMR (CDCl_3_) see Table 2; (+)-ESIMS *m*/*z* 385.2 [M + Na]^+^; (+)-HRESIMS *m*/*z* 385.1991 [M + Na]^+^ (calcd for C_21_H_30_O_5_Na, 385.1991).

### Formosin D (**4**)

White powder; $[\alpha ]_{\text{D}}^{21}$ −164 (*c* 0.025, MeOH); UV (MeOH) *λ*_max_ (log *ε*) 204 (4.21); IR (KBr) *ν*_max_ 3427, 3000–2500, 1695, 1452, 1254, 1082 cm^−1^; ^1^H NMR (CDCl_3_), see Table 1 and ^13^C NMR (CDCl_3_) see Table 2; (+)-ESIMS *m*/*z* 369.2 [M + Na]^+^; (+)-HRESIMS *m*/*z* 369.1970 [M + Na]^+^ (calcd for C_21_H_30_O_4_Na, 369.2036).

### Formosin E (**5**)

White powder; $[\alpha ]_{\text{D}}^{21}$ −20 (*c* 0.05, MeOH); UV (MeOH) *λ*_max_ (log *ε*) 199 (3.89); IR (KBr) *ν*_max_ 3419, 3000–2500, 1695, 1452, 1383, 1244, 1026, 874, 600 cm^−1^; ^1^H NMR (CDCl_3_), see Table 1 and ^13^C NMR (CDCl_3_) see Table 2; (+)-ESIMS *m*/*z* 355.2 [M + Na]^+^; (+)-HRESIMS *m*/*z* 355.1885 [M + Na]^+^ (calcd for C_20_H_28_O_4_Na, 355.1888).

### Formosin F (**6**)

White powder; $[\alpha ]_{\text{D}}^{21}$ −175 (*c* 0.10, MeOH); UV (MeOH) *λ*_max_ (log *ε*) 237 (4.86); IR (KBr) *ν*_max_ 3000–2500, 1703, 1633, 1435, 1381, 1219, 1026, 874 cm^−1^; ^1^H NMR (CDCl_3_), see Table 1 and ^13^C NMR (CDCl_3_) see Table 2; (+)-ESIMS *m*/*z* 331 [M + H]^+^; (+)-HRESIMS *m*/*z* 353.1724 [M + Na]^+^ (calcd for C_20_H_26_O_4_Na, 353.1723).

### Antimicrobial Activity Assay

The in vitro antibacterial activities against *Bacillus subtilis* ATCC 6633 were tested by applying the protocols described in our previous research [13].

Antibacterial tests against *Helicobacter pylori* strains (*Hp*-SS1 or ATCC 43504 strain) were carried out in vitro according to the protocols described previously [14].

## Electronic supplementary material

Supplementary material 1 (PDF 3792 kb) **Electronic supplementary material** The online version of this article (doi:) contains supplementary material, which is available to authorized users
