# Supplementary material for: Formosins A–F: Diterpenoids with Anti-microbial Activities from Excoecaria formosana
Source: Nat Prod Bioprospect. 2016 Jan 27;6(1):57–61. doi: 10.1007/s13659-016-0086-6 (PMC4749522; doi:10.1007/s13659-016-0086-6)

Supporting Information for

Formosins A–F: Diterpenoids with Anti-microbial Activities from *Excoecaria formosana*

Bing-Dong Lin, Bin Zhou, Lei Dong, Yan Wu, and Jian-Min Yue*

State Key Laboratory of Drug Research, Shanghai Institute of Materia Medica, Chinese Academy of Sciences, 555 Zuchongzhi Road, Shanghai 201203, People's Republic of China

Figure S1. ^{1}H NMR spectrum of formosin A (**1**) in $CDCl_3$

Figure S2. ^{13}C NMR spectrum of formosin A (**1**) in $CDCl_3$

Figure S3. HSQC spectrum of formosin A (**1**) in $CDCl_3$

Figure S4. HMBC spectrum of formosin A (**1**) in $CDCl_3$

Figure S5. ROESY spectrum of formosin A (**1**) in $CDCl_3$

Figure S6. (+)-ESIMS spectrum of formosin A (**1**)

Figure S7. (−)-ESIMS spectrum of formosin A (**1**)

Figure S8. (+)-HRESIMS spectrum of formosin A (**1**)

Figure S9. IR spectrum of formosin A (**1**)

Figure S10. ^{1}H NMR spectrum of formosin B (**2**) in $CDCl_3$

Figure S11. ^{13}C NMR spectrum of formosin B (**2**) in $CDCl_3$

Figure S12. HSQC spectrum of formosin B (**2**) in $CDCl_3$

Figure S13. HMBC spectrum of formosin B (**2**) in $CDCl_3$

Figure S14. (+)-ESIMS spectrum of formosin B (**2**)

Figure S15. (−)-ESIMS spectrum of formosin B (**2**)

Figure S16. (+)-HRESIMS spectrum of formosin B (**2**)

Figure S17. IR spectrum of formosin B (**2**)

Figure S18. ^{1}H NMR spectrum of formosin C (**3**) in $CDCl_3$

Figure S19. ^{13}C NMR spectrum of formosin C (**3**) in $CDCl_3$

Figure S20. HSQC spectrum of formosin C (**3**) in $CDCl_3$

Figure S21. HMBC spectrum of formosin C (**3**) in $CDCl_3$

Figure S22. ROESY spectrum of formosin C (**3**) in $CDCl_3$

Figure S23. (+)-ESIMS spectrum of formosin C (**3**)

Figure S24. (−)-ESIMS spectrum of formosin C (**3**)

Figure S25. (+)-HRESIMS spectrum of formosin C (**3**)

Figure S26. IR spectrum of formosin C (**3**)

Figure S27. ^{1}H NMR spectrum of formosin D (**4**) in $CDCl_3$

Figure S28. ^{13}C NMR spectrum of formosin D (**4**) in $CDCl_3$

Figure S29. HSQC spectrum of formosin D (**4**) in $CDCl_3$

Figure S30. HMBC spectrum of formosin D (**4**) in $CDCl_3$

Figure S31. ROESY spectrum of formosin D (**4**) in $CDCl_3$

Figure S32. (+)-ESIMS spectrum of formosin D (**4**)

Figure S33. (−)-ESIMS spectrum of formosin D (**4**)

Figure S34. (+)-HRESIMS spectrum of formosin D (**4**)

Figure S35. IR spectrum of formosin D (**4**)

Figure S36. ^{1}H NMR spectrum of formosin E (**5**) in $CDCl_3$

Figure S37. ^{13}C NMR spectrum of formosin E (**5**) in $CDCl_3$

Figure S38. HSQC spectrum of formosin E (**5**) in $CDCl_3$

Figure S39. HMBC spectrum of formosin E (**5**) in $CDCl_3$

Figure S40. ROESY spectrum of formosin E (**5**) in $CDCl_3$

Figure S41. (+)-ESIMS spectrum of formosin E (**5**)

Figure S42. (−)-ESIMS spectrum of formosin E (**5**)

Figure S43. (+)-HRESIMS spectrum of formosin E (**5**)

Figure S44. IR spectrum of formosin E (**5**)

Figure S45. ^{1}H NMR spectrum of formosin F (**6**) in $CDCl_3$

Figure S46. ^{13}C NMR spectrum of formosin F (**6**) in $CDCl_3$

Figure S47. HSQC spectrum of formosin F (**6**) in $CDCl_3$

Figure S48. HMBC spectrum of formosin F (**6**) in $CDCl_3$

Figure **S49**. (+)-ESIMS spectrum of formosin F (**6**)

Figure S50. (−)-ESIMS spectrum of formosin F (**6**)

Figure S51. (+)-HRESIMS spectrum of formosin F (**6**)

Figure S52. IR spectrum of formosin F (**6**)

Figure S1. ^{1}H NMR spectrum of formosin A (**1**) in $CDCl_3$

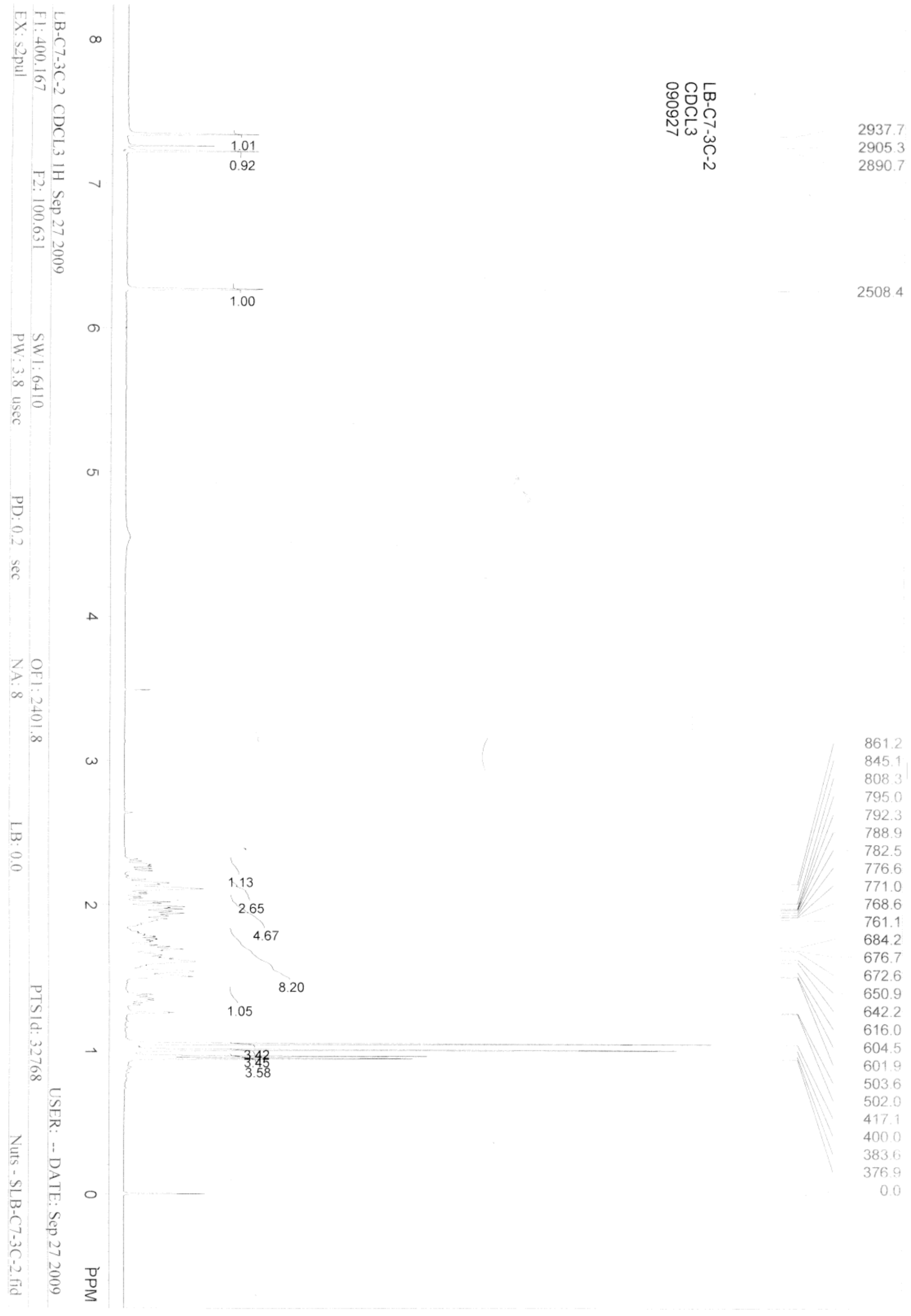

Figure S2. ^{13}C NMR spectrum of formosin A (**1**) in $CDCl_3$

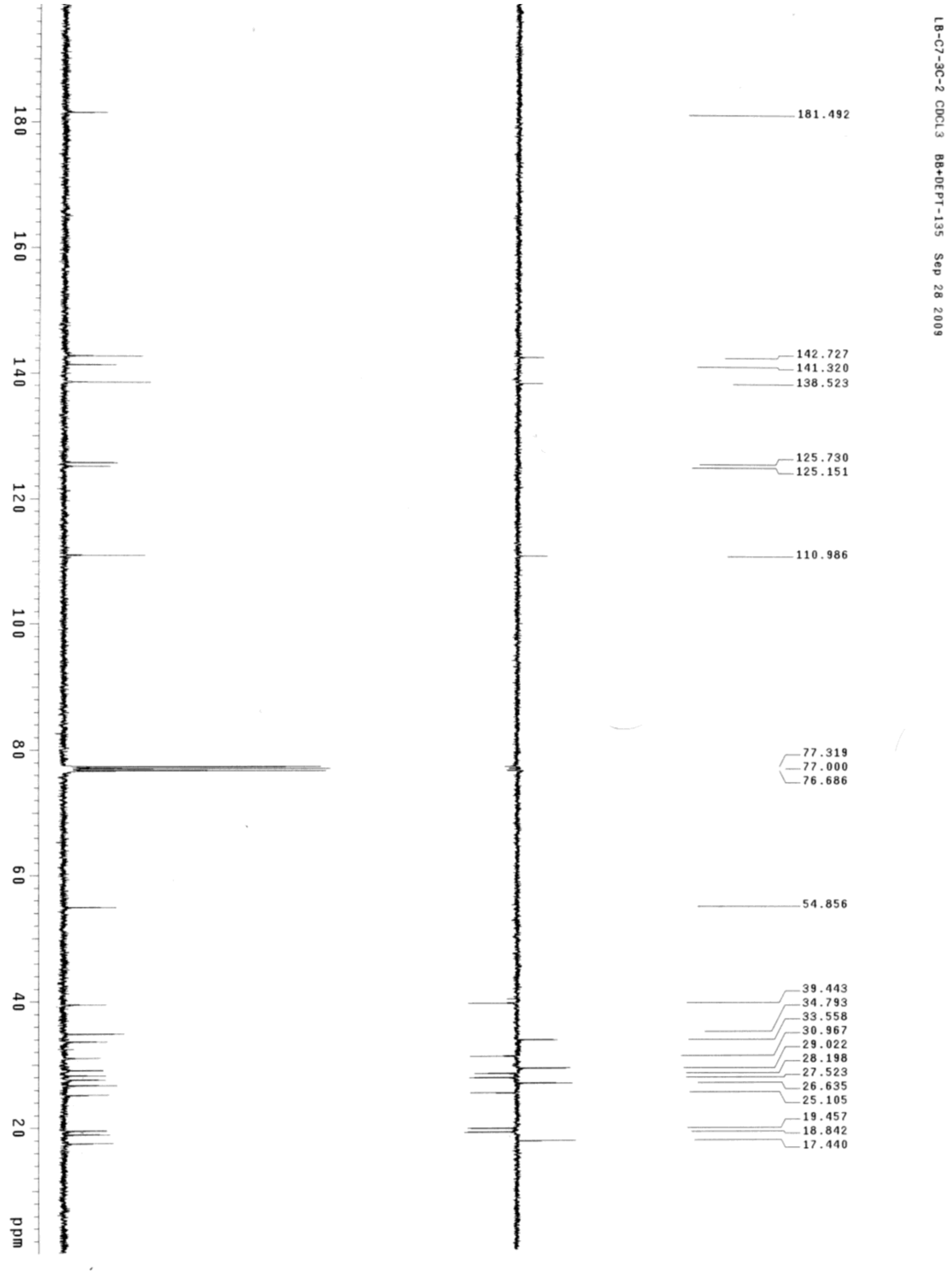

Figure S3. HSQC spectrum of formosin A (**1**) in $CDCl_3$

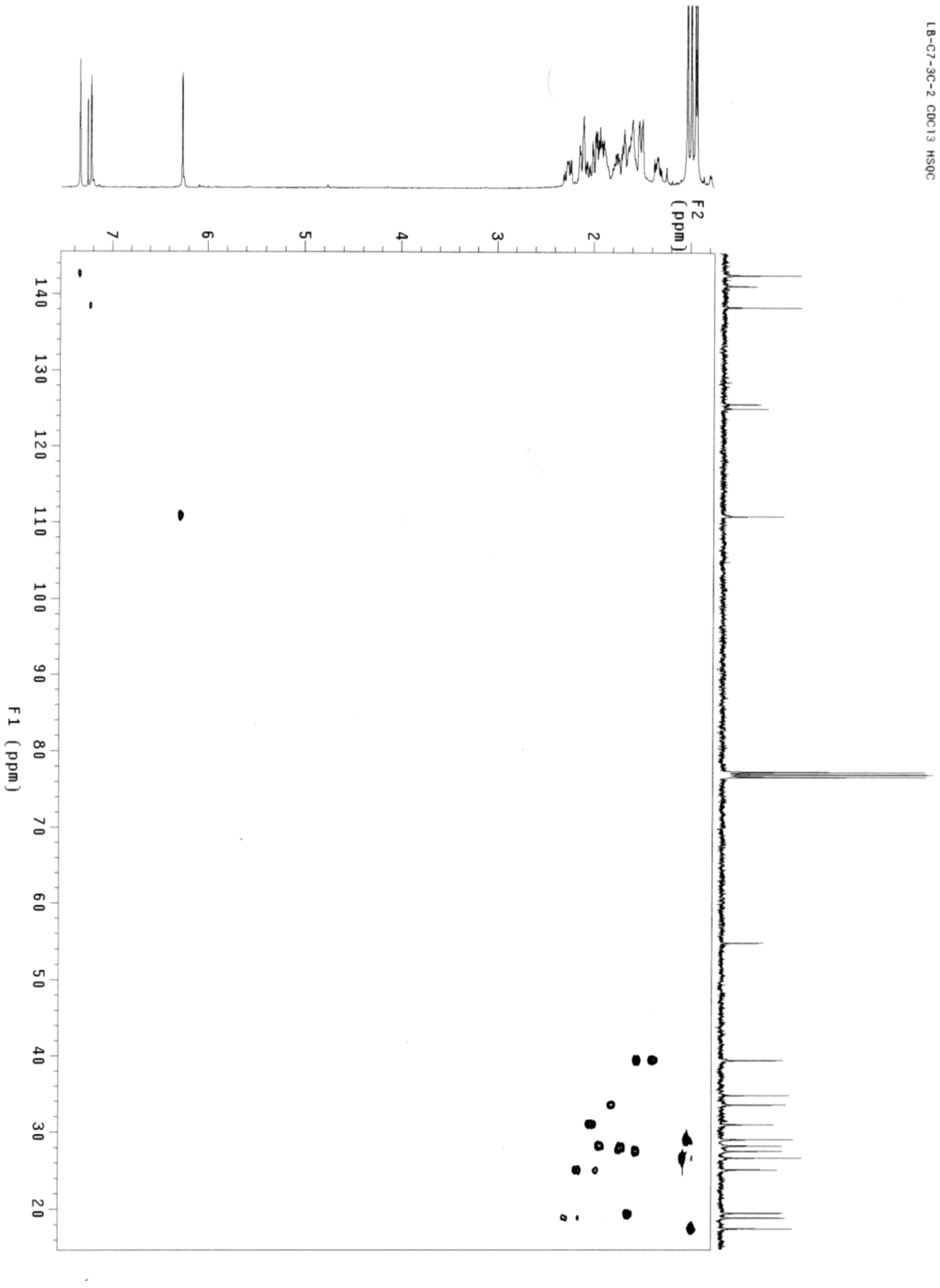

Figure S4. HMBC spectrum of formosin A (**1**) in $CDCl_3$

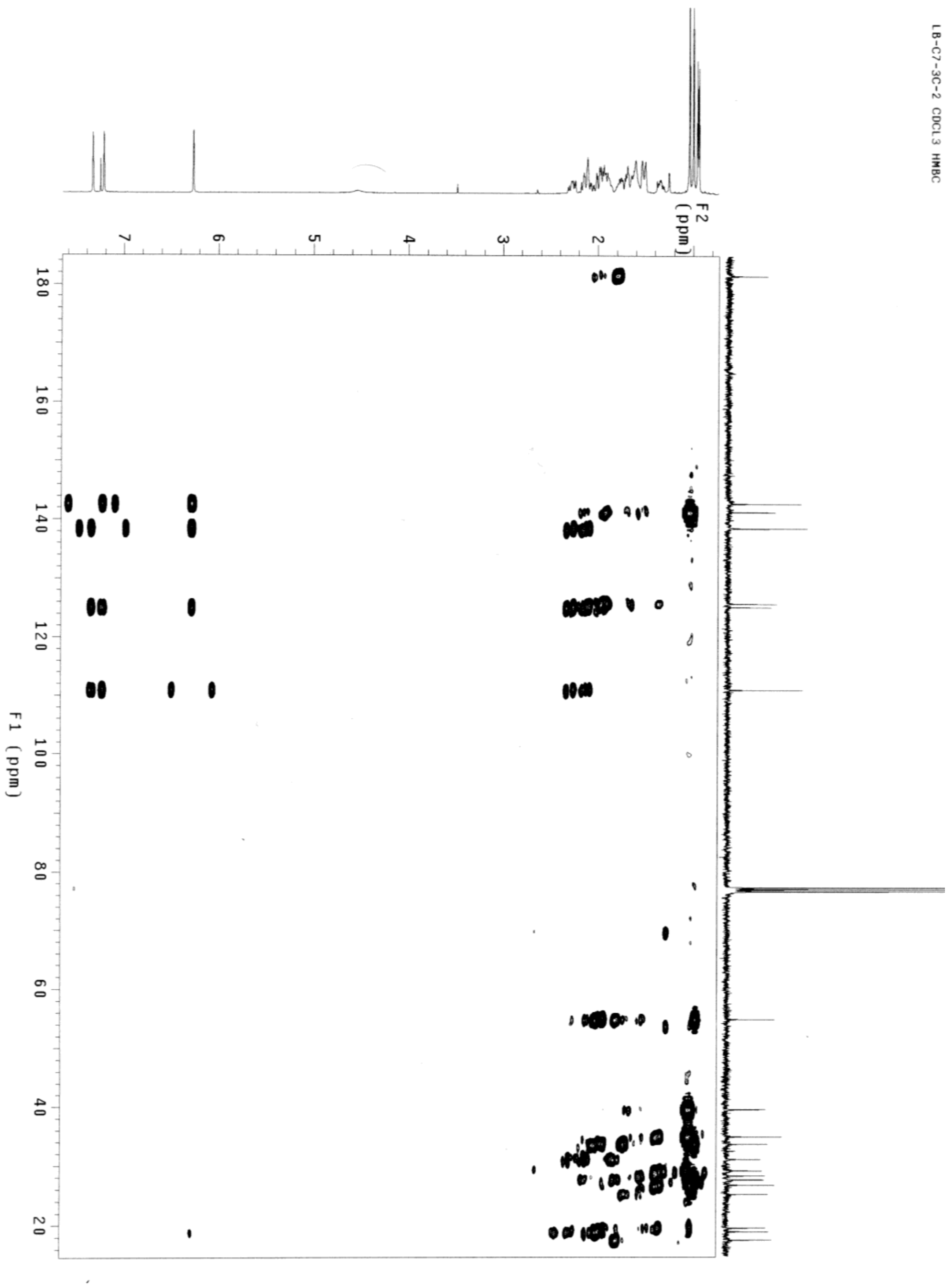

Figure S5. ROESY spectrum of formosin A (**1**) in $CDCl_3$

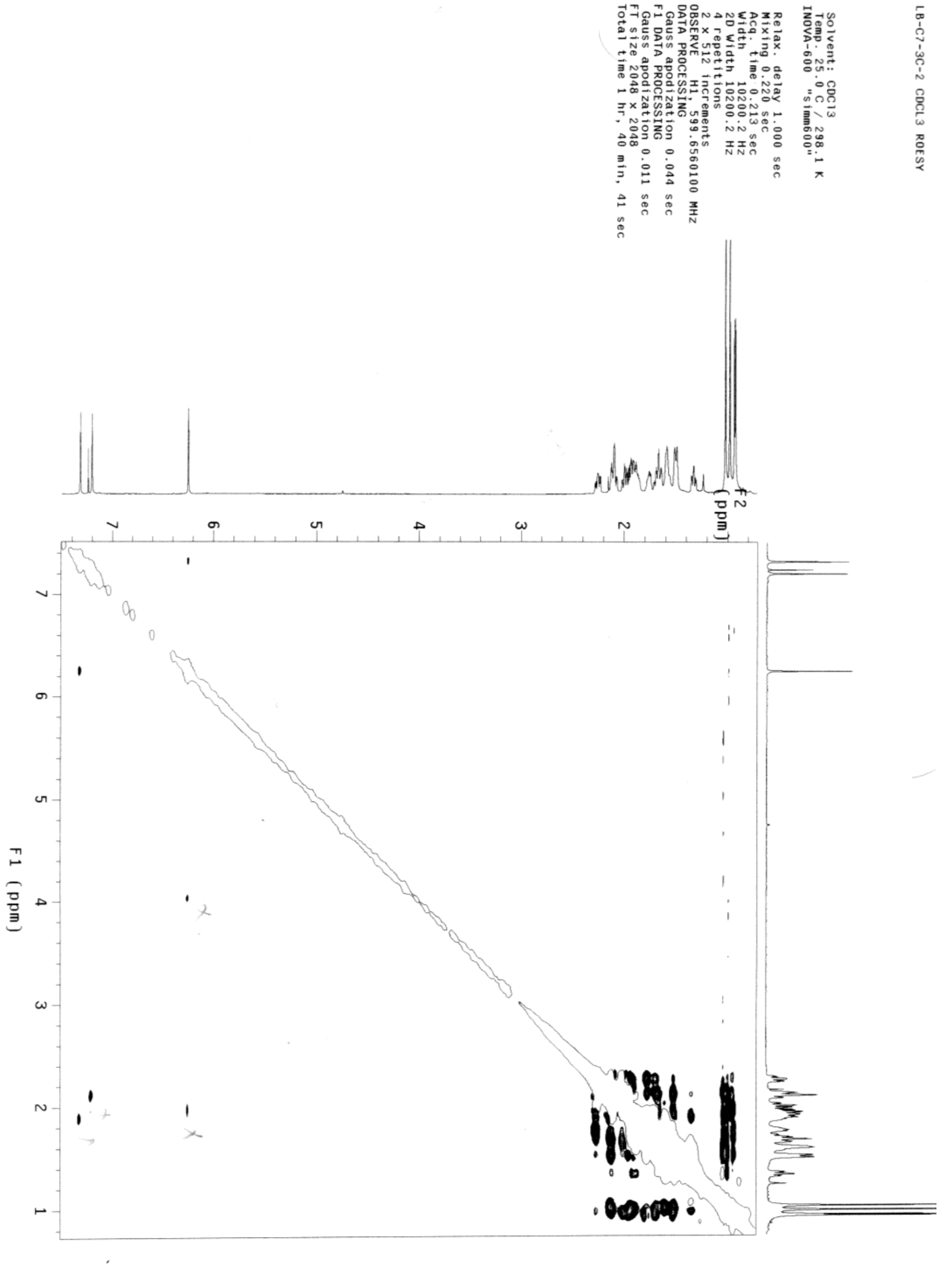

Figure S6. (+)-ESIMS spectrum of formosin A (**1**)

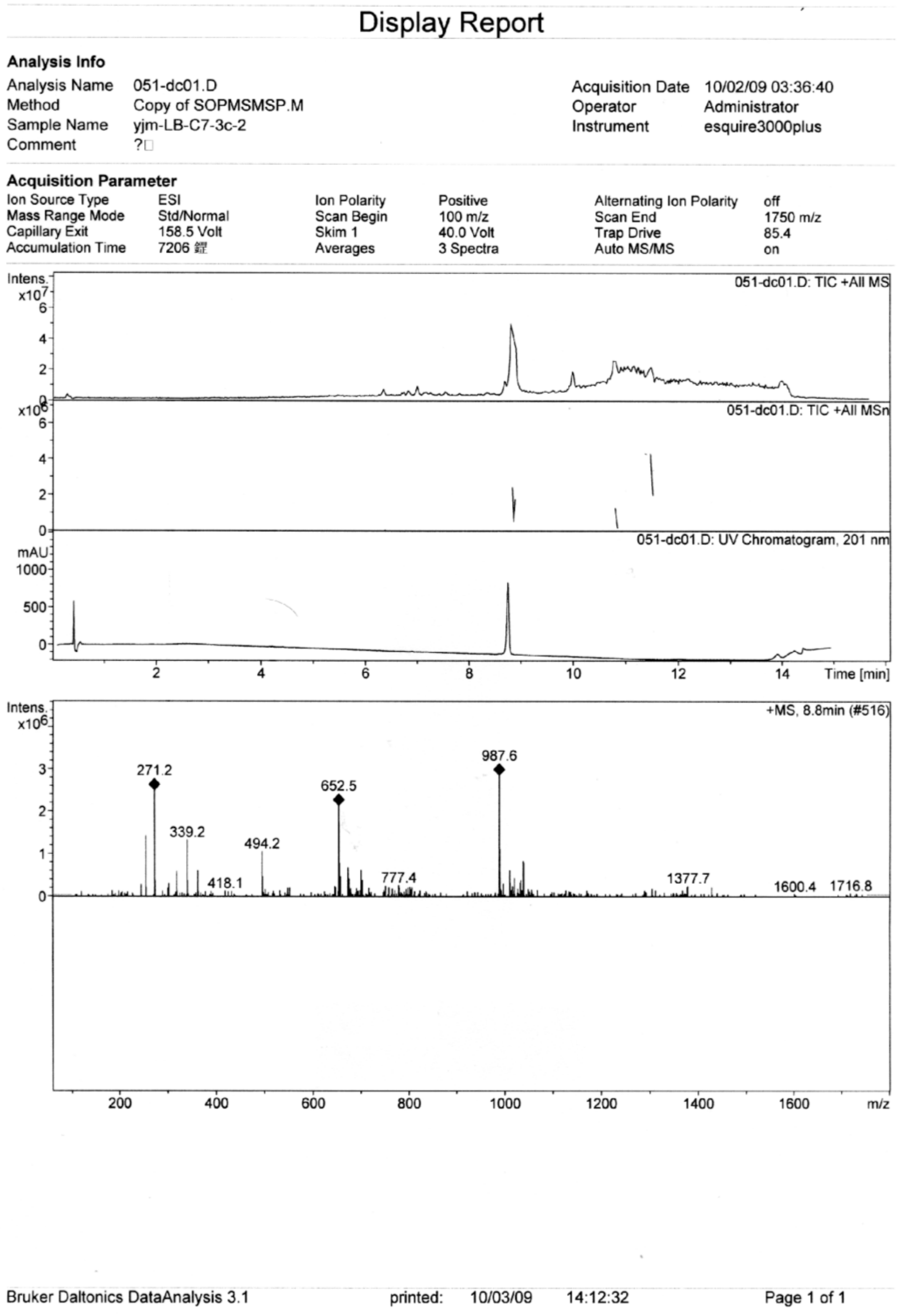

Figure S7. (−)-ESIMS spectrum of formosin A (**1**)

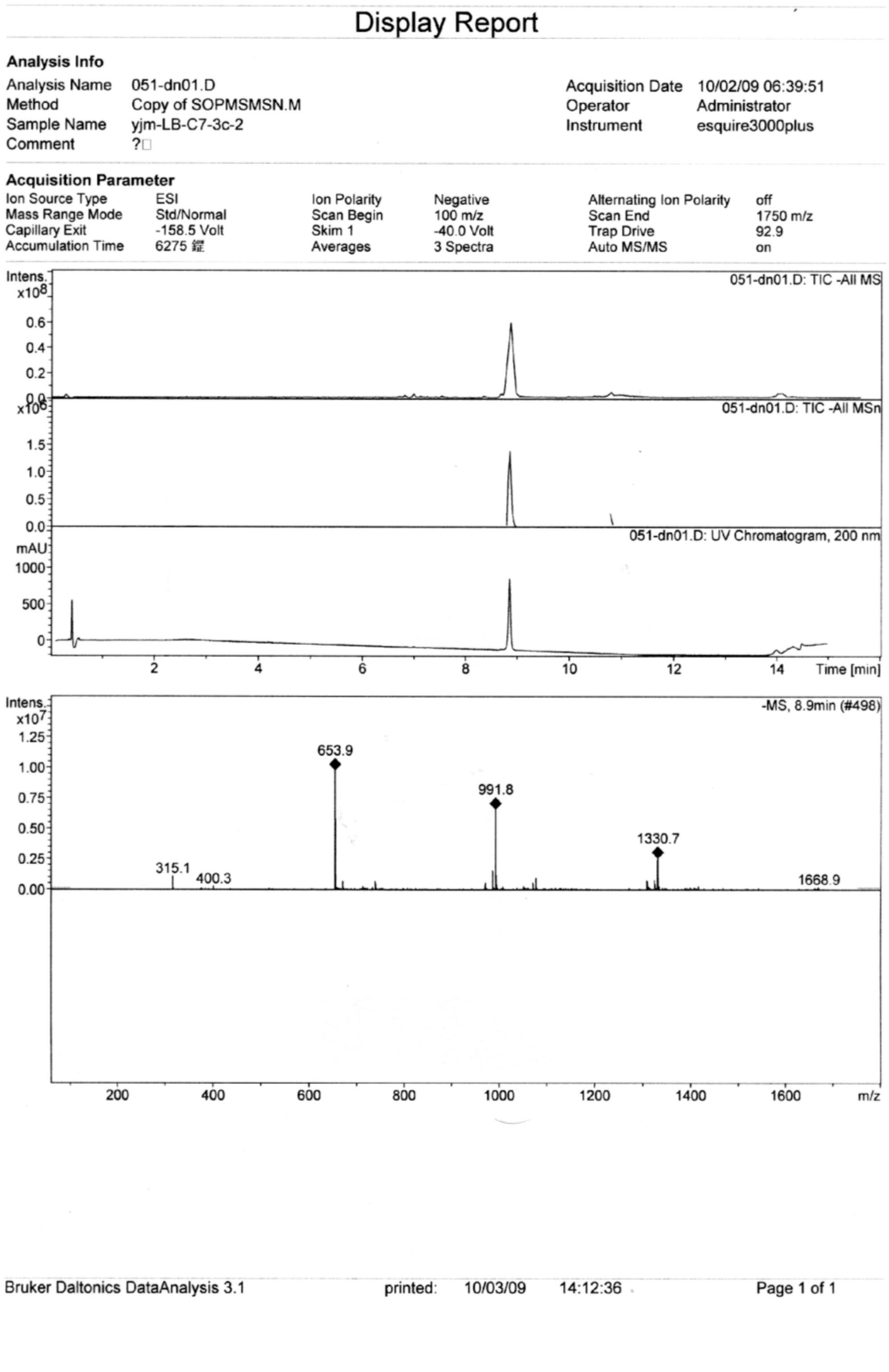

Figure S8. (+)-HRESIMS spectrum of formosin A (**1**)

Mass Spectrum SmartFormula Report

Analysis Info

Analysis Name	D:\Data\FH\LB-C7-3C-2.d	Acquisition Date	6/12/2010 10:19:20 PM
Method	tune_wide.m	Operator	ECNU
Sample Name	LB-C7-3C-2	Instrument / Ser#	micrOTOF-Q 10198
Comment			

Acquisition Parameter

Source Type	ESI	Ion Polarity	Positive	Set Nebulizer	0.4 Bar
Focus	Active	Set Capillary	4500 V	Set Dry Heater	180 °C
Scan Begin	50 m/z	Set End Plate Offset	-500 V	Set Dry Gas	4.0 l/min
Scan End	3000 m/z	Set Collision Cell RF	600.0 Vpp	Set Divert Valve	Source

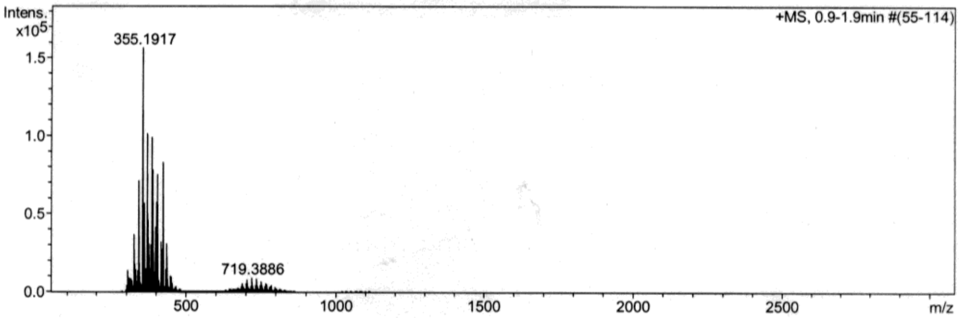

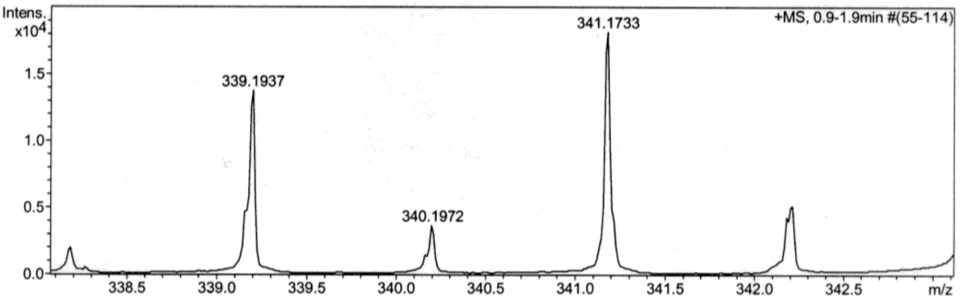

Meas. m/z	#	Formula	m/z	err [ppm]	Mean err [ppm]	N-Rule	e⁻ Conf	mSigma	Std I	Std Mean m/z	Std m/z Diff
339.1937											
	1	C 20 H 28 Na O 3	339.1931	-1.9	-2.0	ok	even	34.77	0.0539	0.0007	0.0002

uker Compass DataAnalysis 4.0 printed: 6/12/2010 10:29:28 PM Page 1 of 1

Figure S9. IR spectrum of formosin A (**1**)

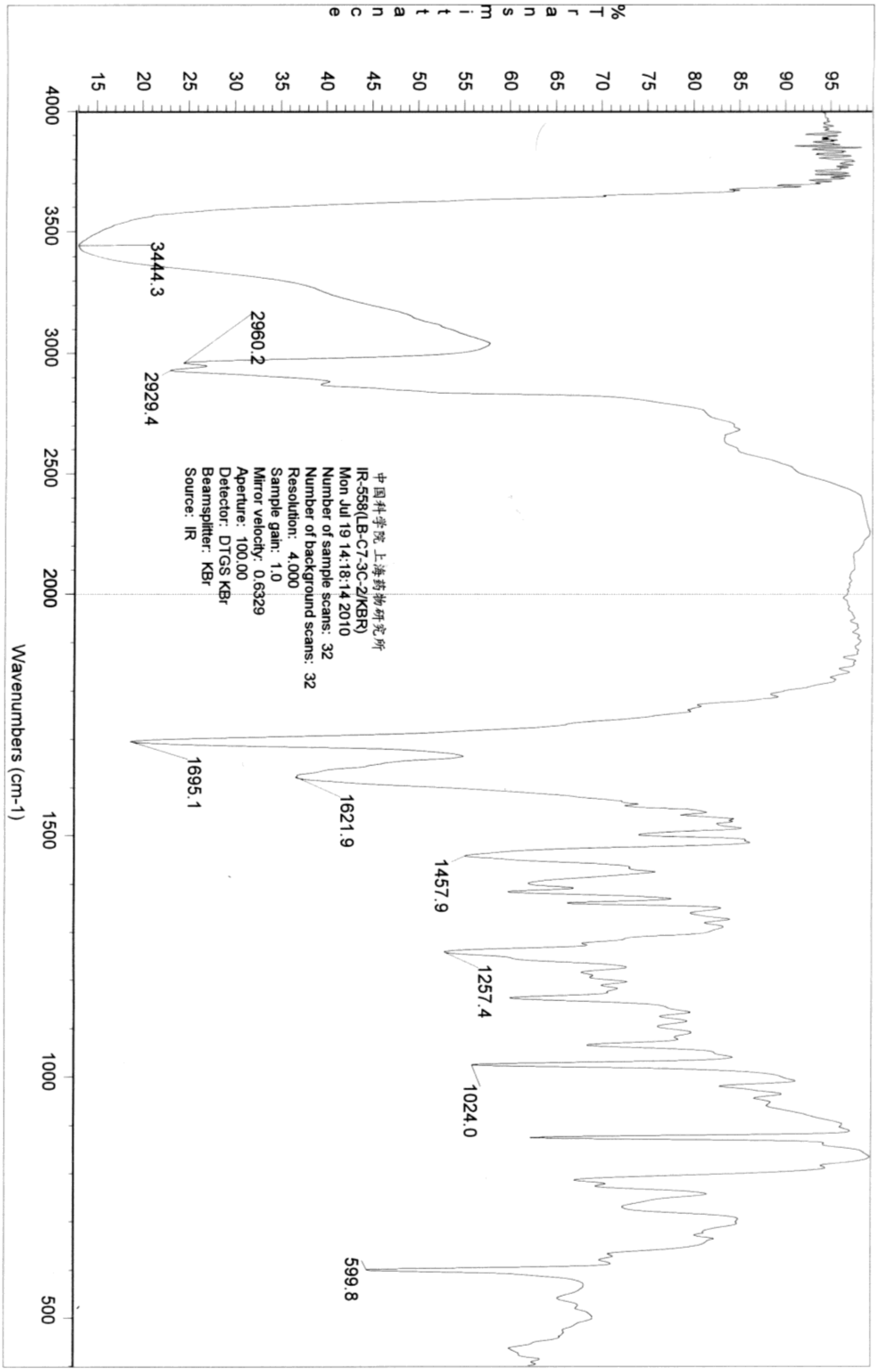

Figure S10. ^{1}H NMR spectrum of formosin B (**2**) in $CDCl_3$

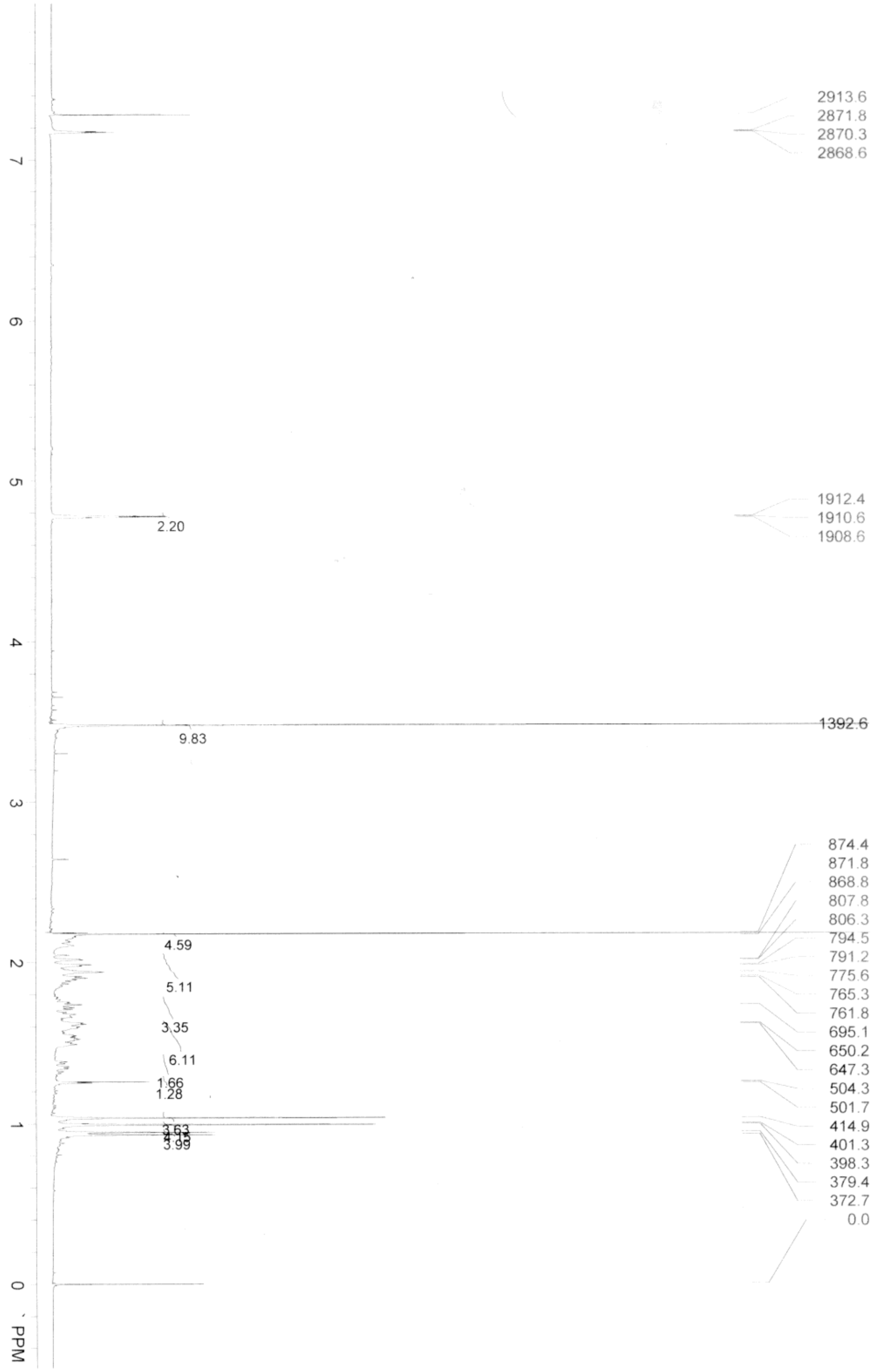

Figure S12. ^{13}C NMR spectrum of formosin B (**2**) in $CDCl_3$

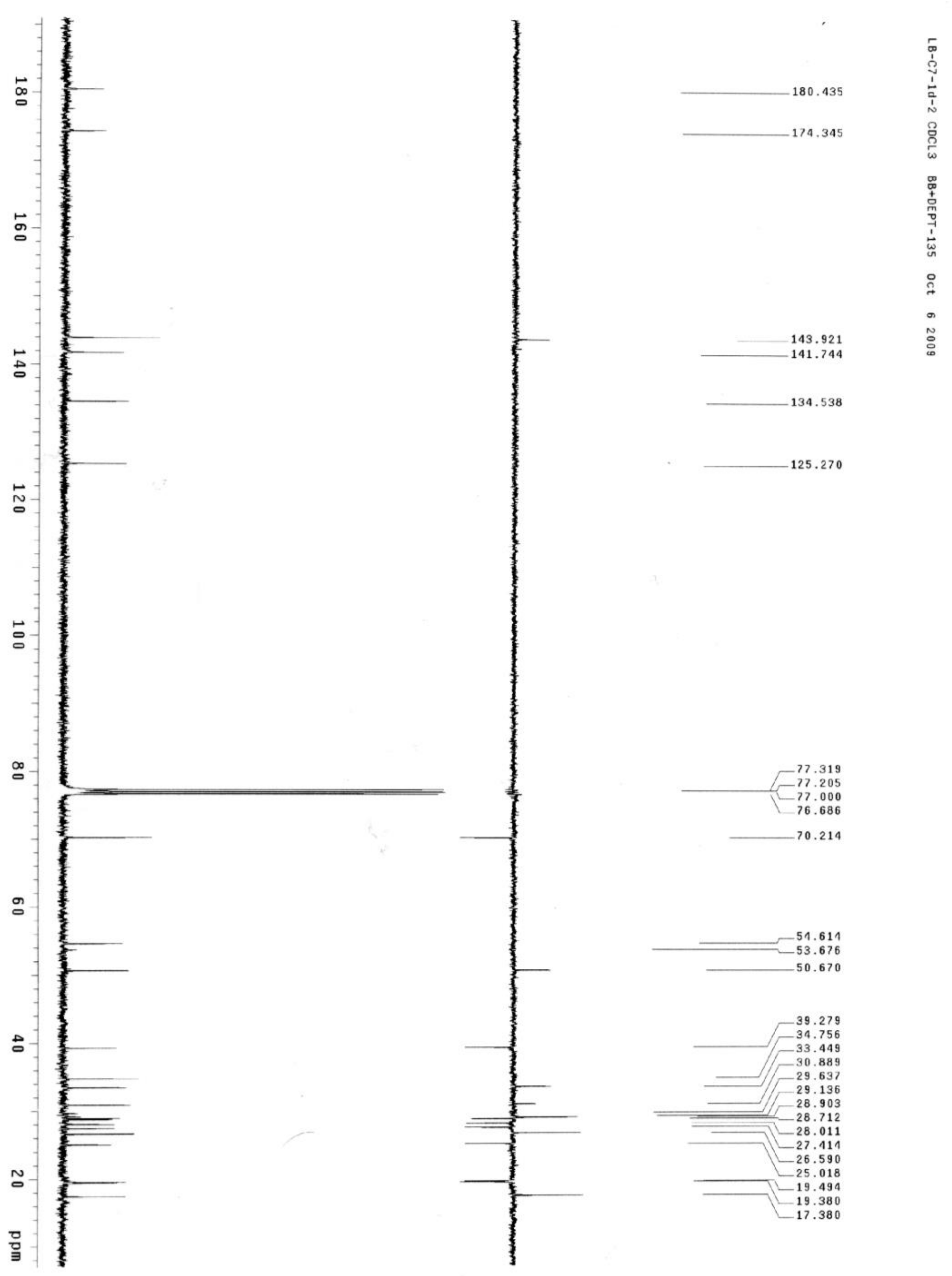

Figure S12. HSQC spectrum of formosin B (**2**) in $CDCl_3$

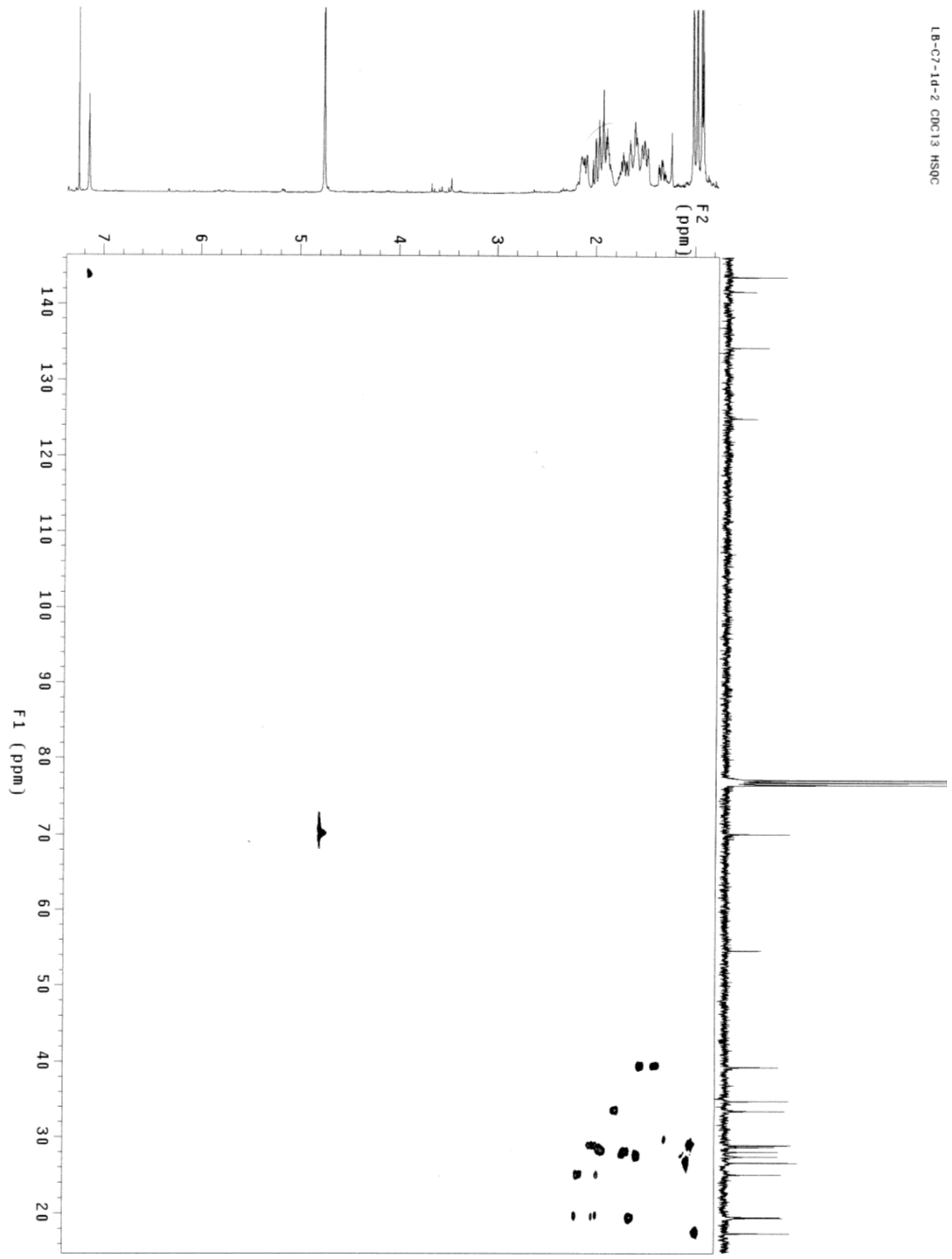

Figure S13. HMBC spectrum of formosin B (**2**) in $CDCl_3$

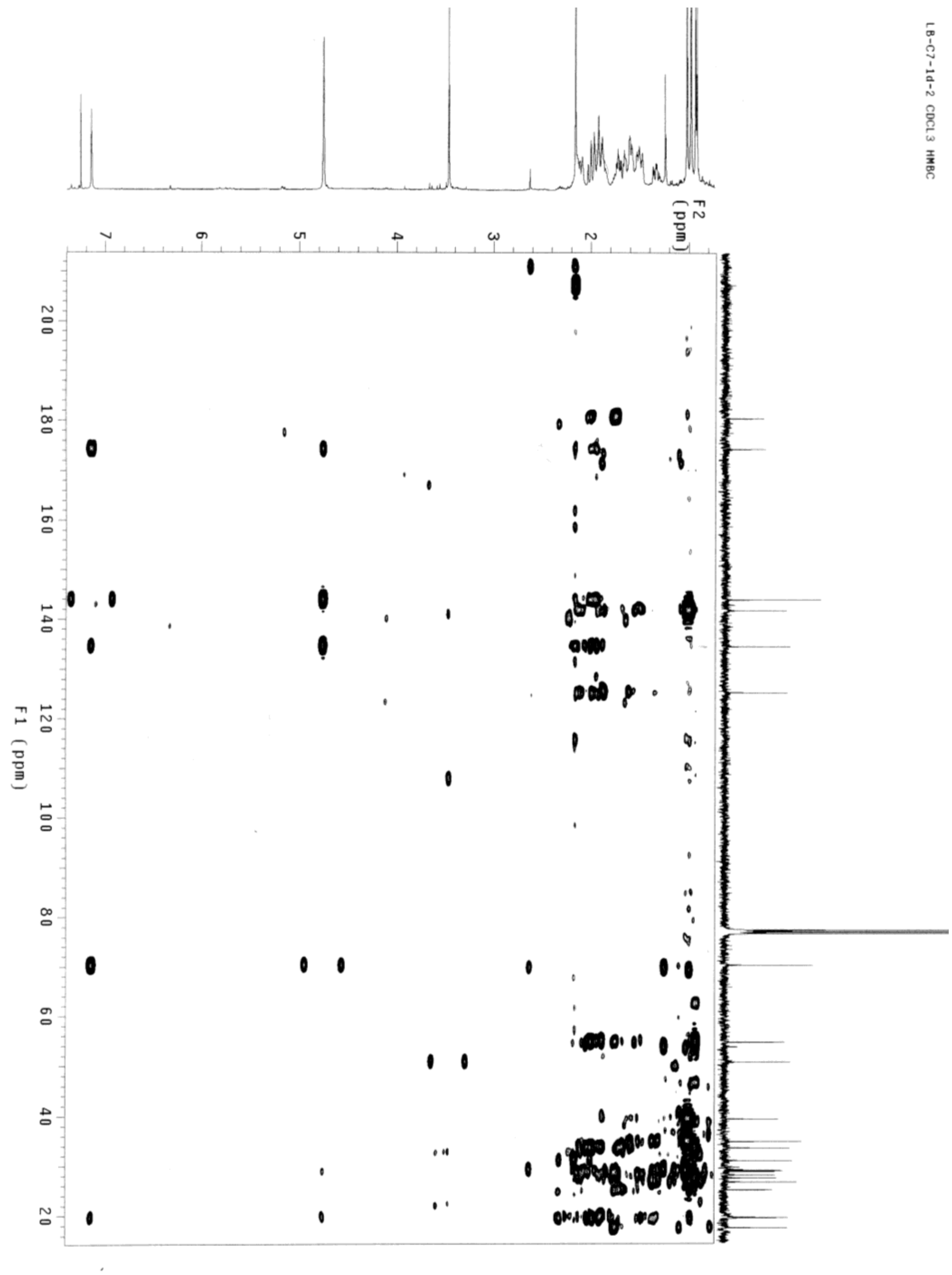

Figure S14. (+)-ESIMS spectrum of formosin B (**2**)

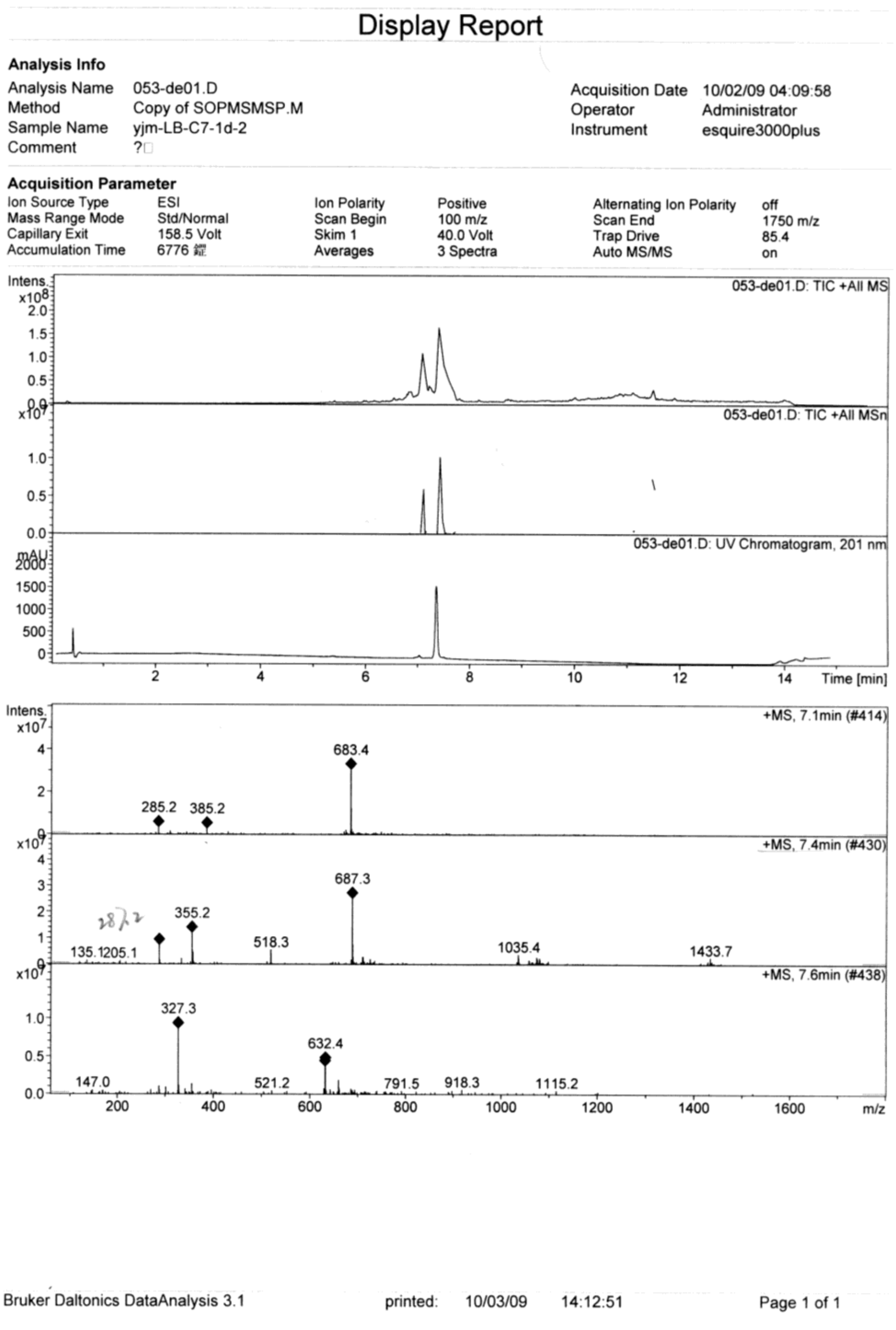

Figure S15. (−)-ESIMS spectrum of formosin B (**2**)

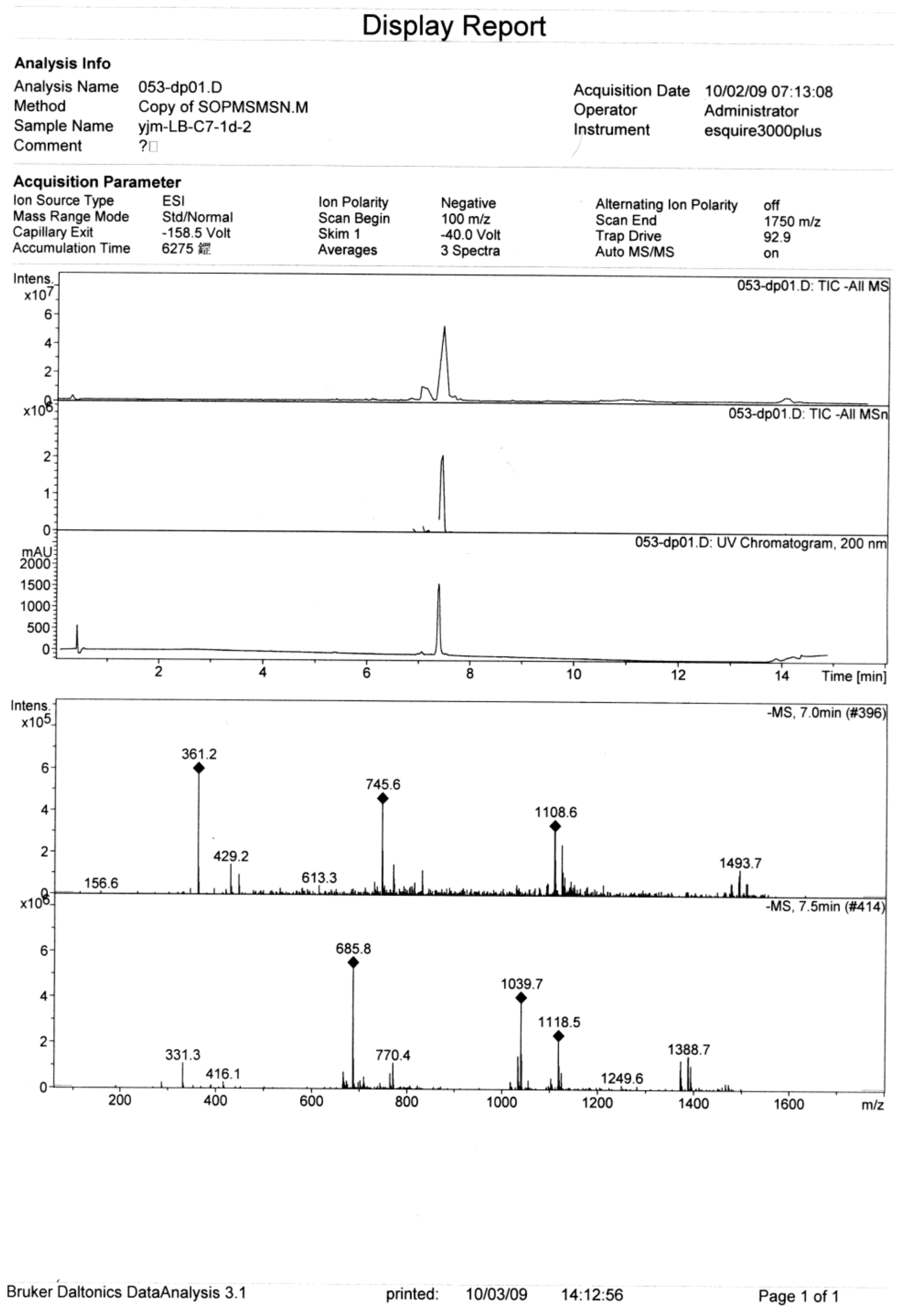

Figure S16. (+)-HRESIMS spectrum of formosin B (**2**)

Mass Spectrum SmartFormula Report

Analysis Info

Analysis Name D:\Data\FH\LB-C7-1d-2.d
Method tune_wide.m
Sample Name LB-C7-1d-2
Comment

Acquisition Date 6/12/2010 10:30:41 PM
Operator ECNU
Instrument / Ser# micrOTOF-Q 10198

Acquisition Parameter

Source Type	ESI	Ion Polarity	Positive	Set Nebulizer	0.4 Bar
Focus	Active	Set Capillary	4500 V	Set Dry Heater	180 °C
Scan Begin	50 m/z	Set End Plate Offset	-500 V	Set Dry Gas	4.0 l/min
Scan End	3000 m/z	Set Collision Cell RF	600.0 Vpp	Set Divert Valve	Source

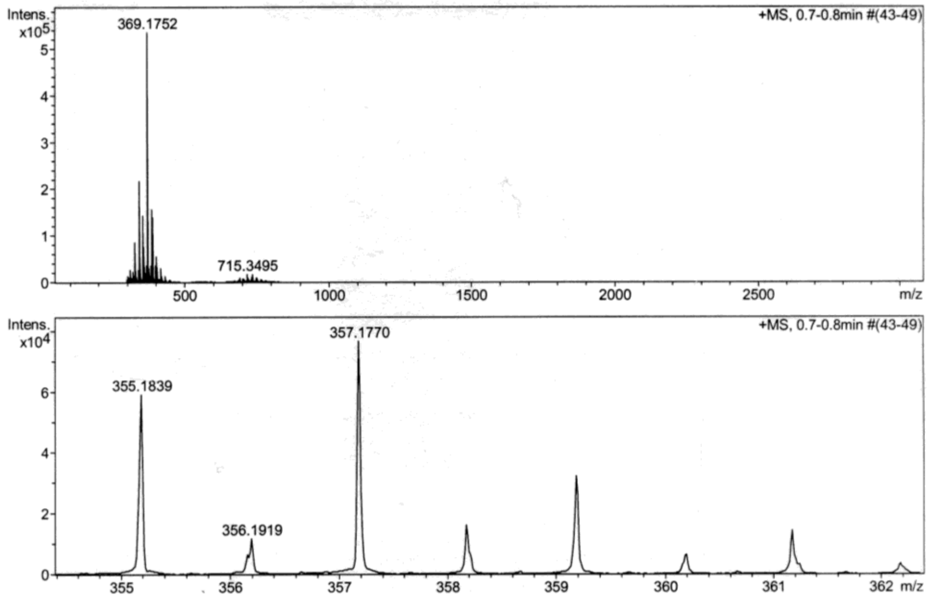

Meas. m/z	#	Formula	m/z	err [ppm]	Mean err [ppm]	N-Rule	e⁻ Conf	mSigma	Std I	Std Mean m/z	Std m/z Diff
355.1839											
	1	C 20 H 28 Na O 4	355.1880	11.6	9.5	ok	even	20.47	0.0306	0.0038	0.0047

Bruker Compass DataAnalysis 4.0 printed: 6/12/2010 10:38:00 PM Page 1 of 1

Figure S17. IR spectrum of formosin B (**2**)

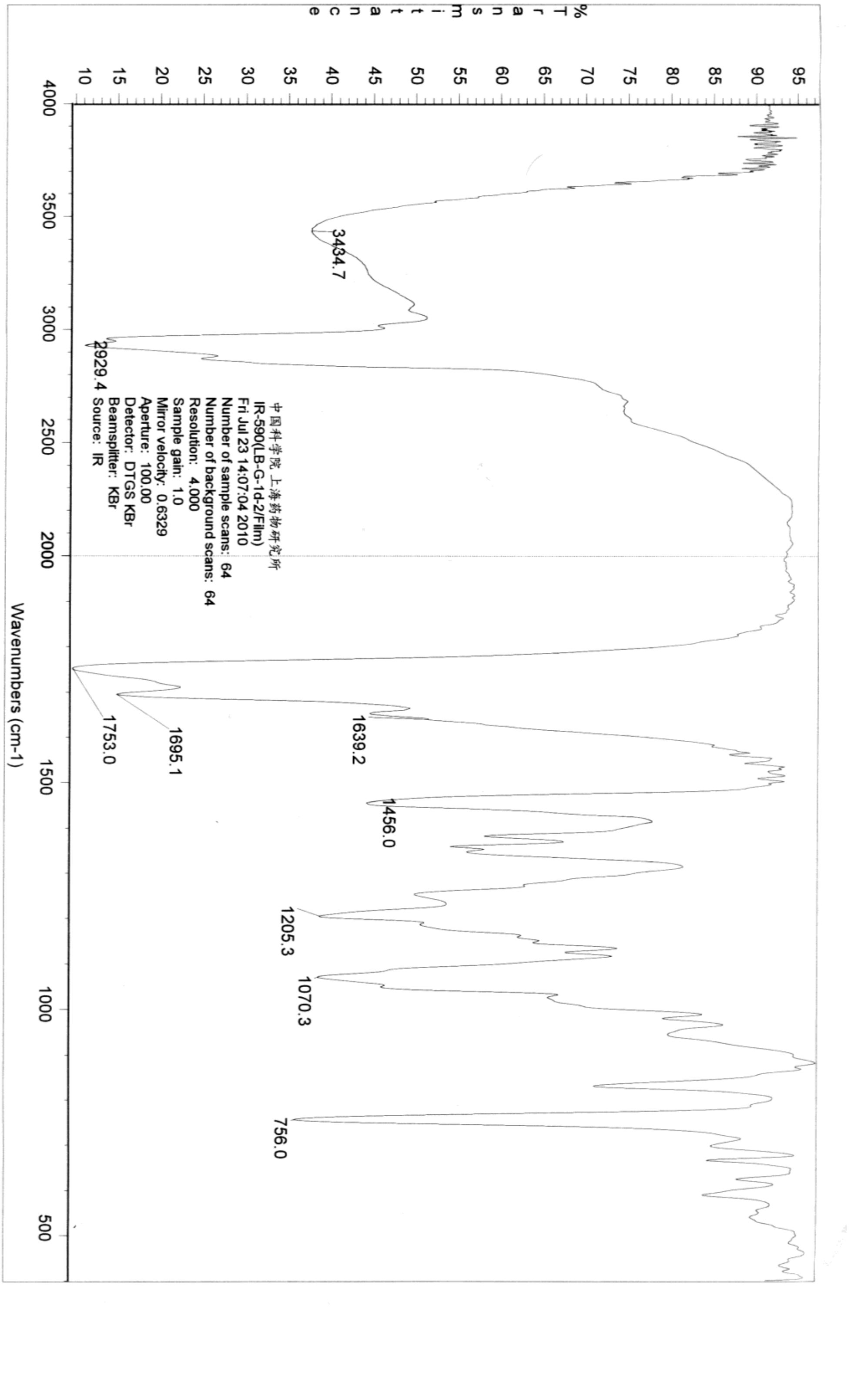

Figure S19. ^{1}H NMR spectrum of formosin C (**3**) in $CDCl_3$

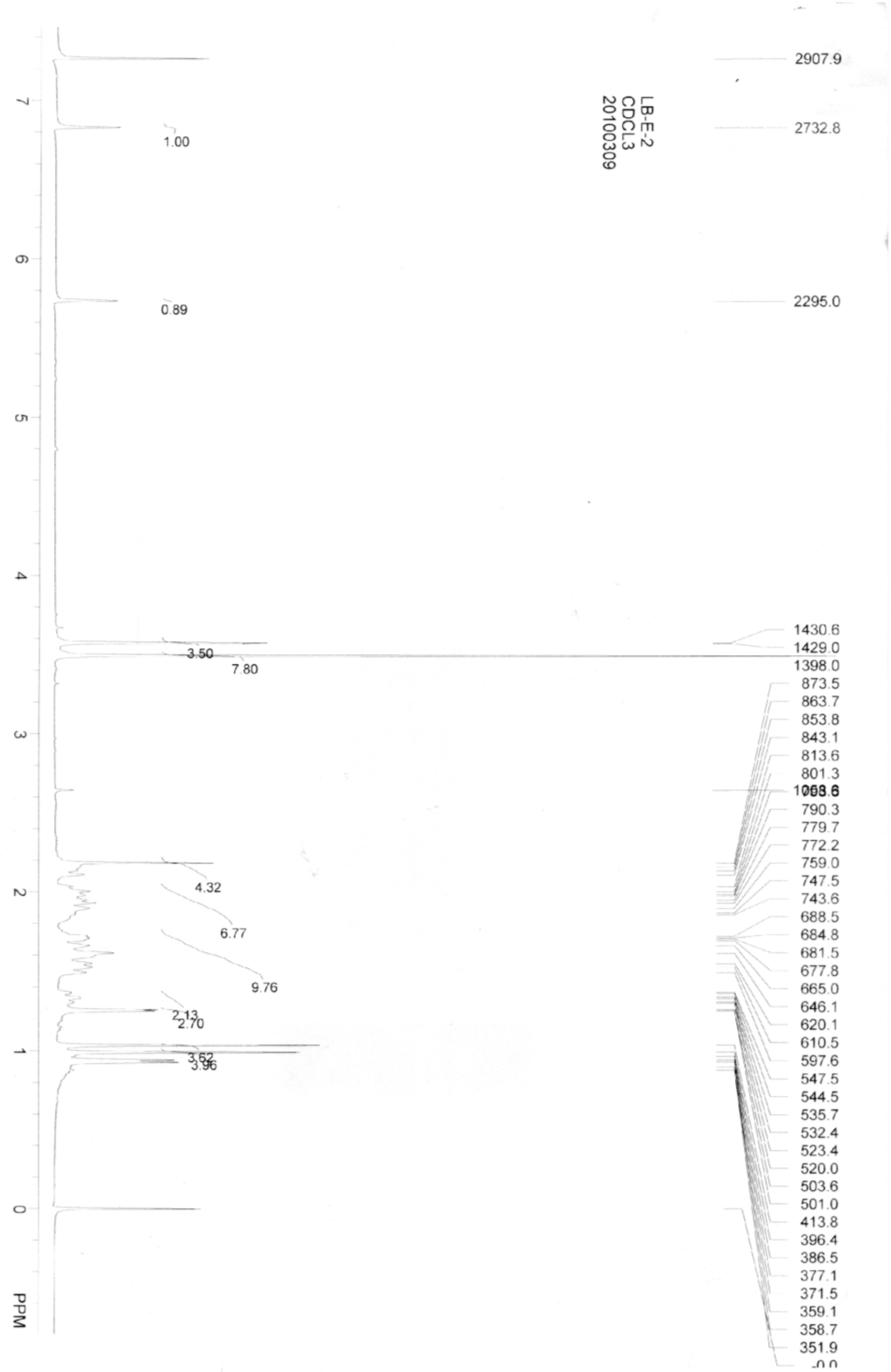

Figure S19. ^{13}C NMR spectrum of formosin C (**3**) in $CDCl_3$

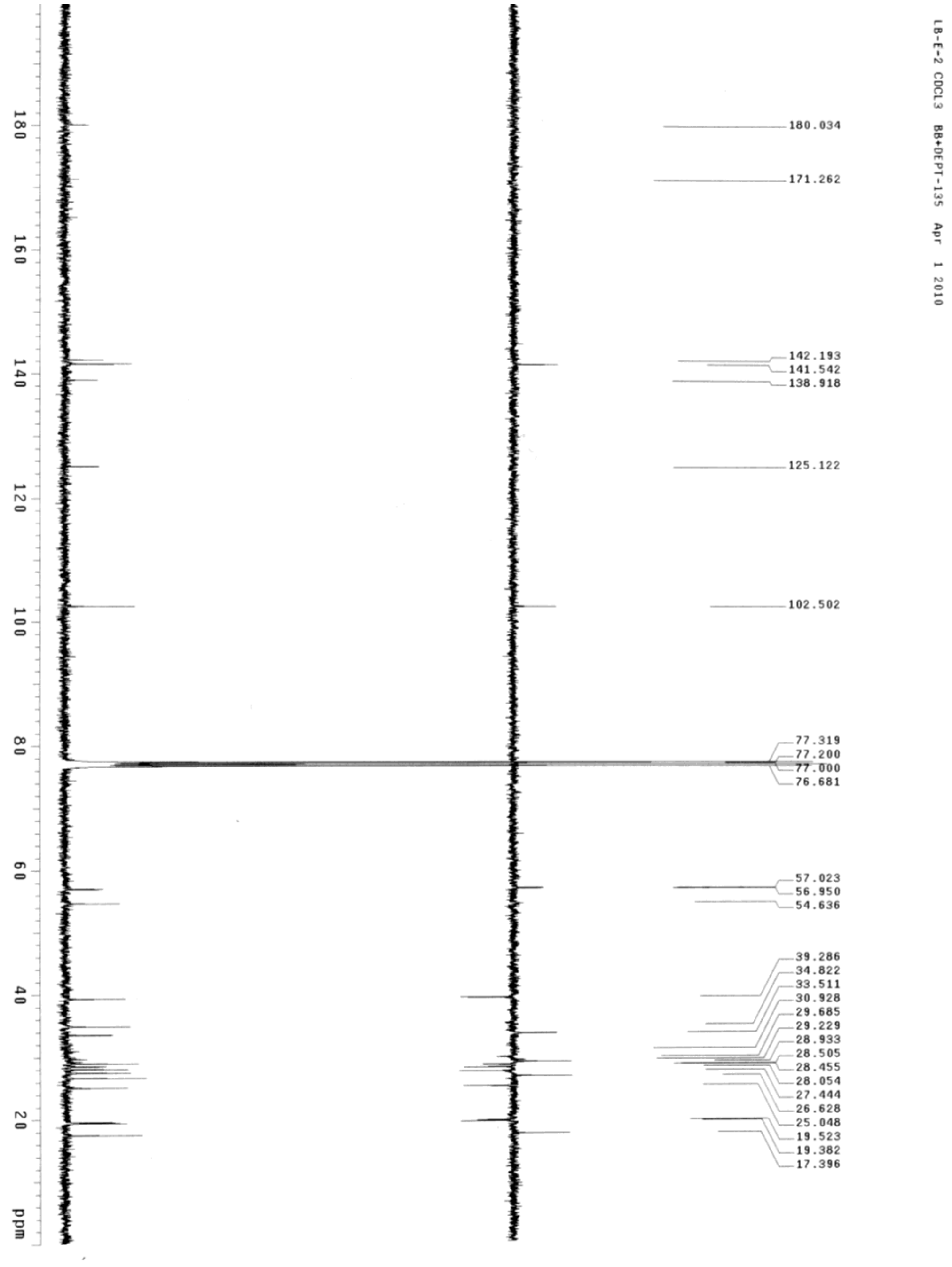

Figure S20. HSQC spectrum of formosin C (**3**)

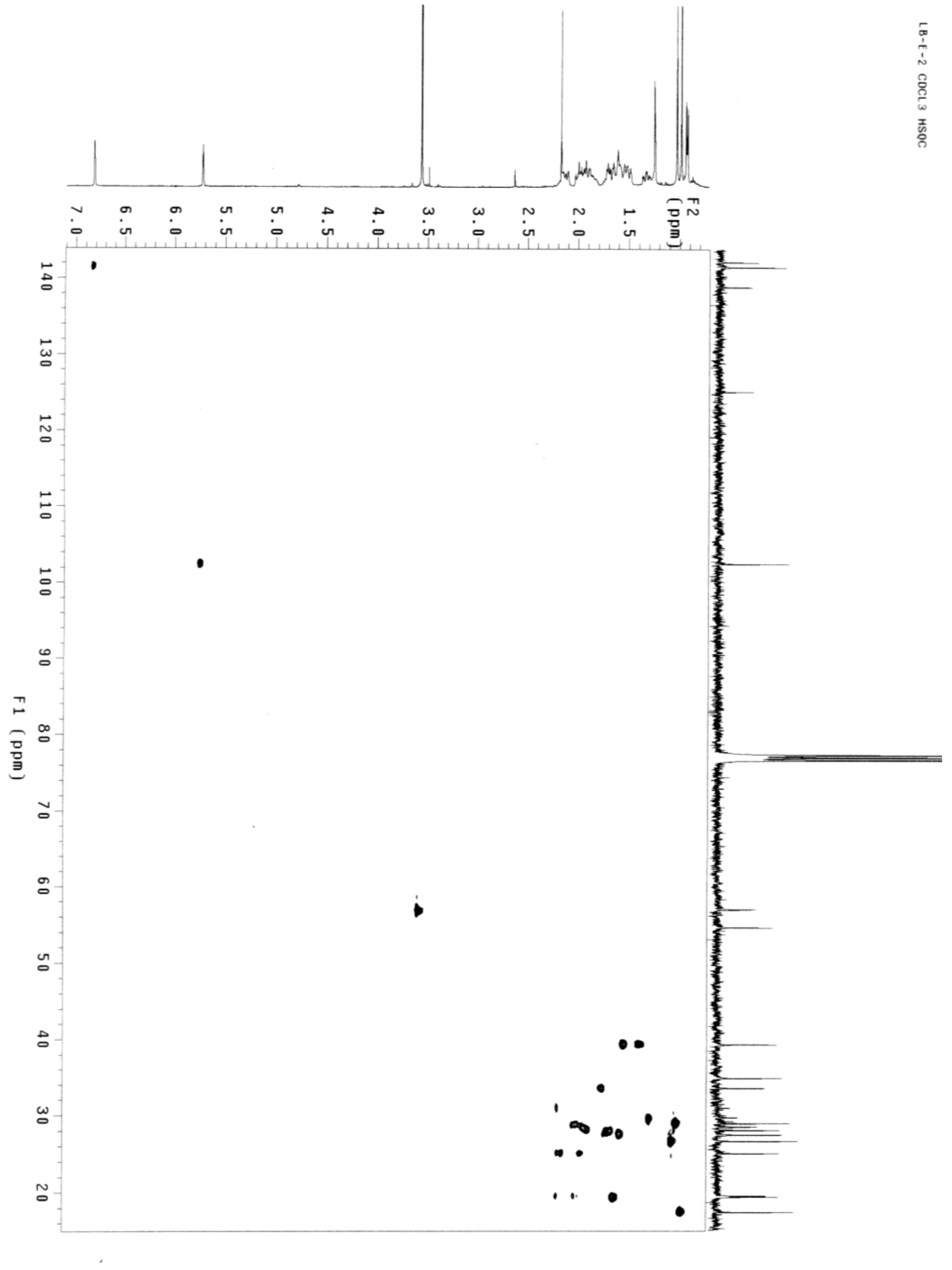

Figure S21. HMBC spectrum of formosin C (**3**)

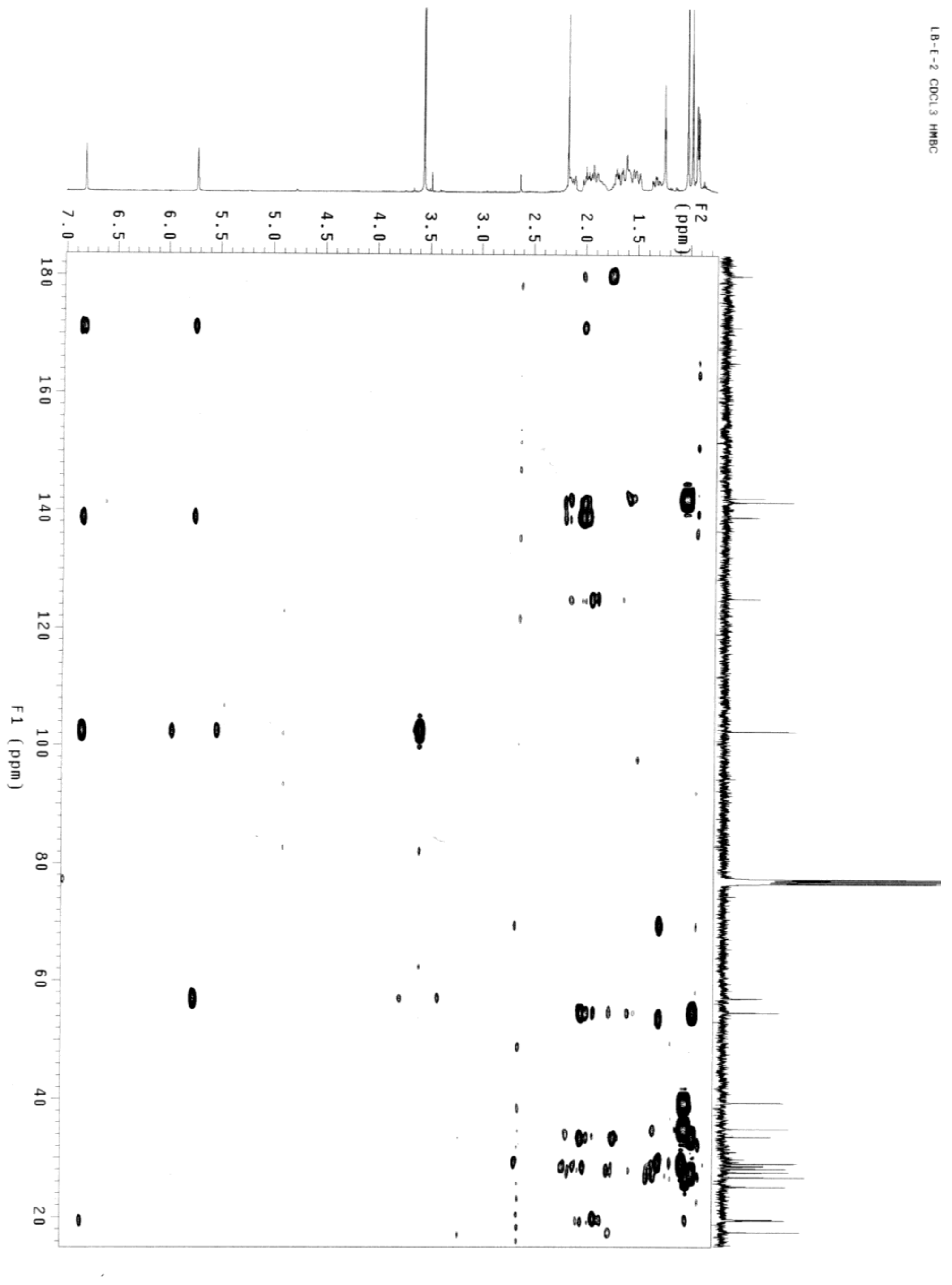

Figure S22. ROESY spectrum of formosin C (**3**)

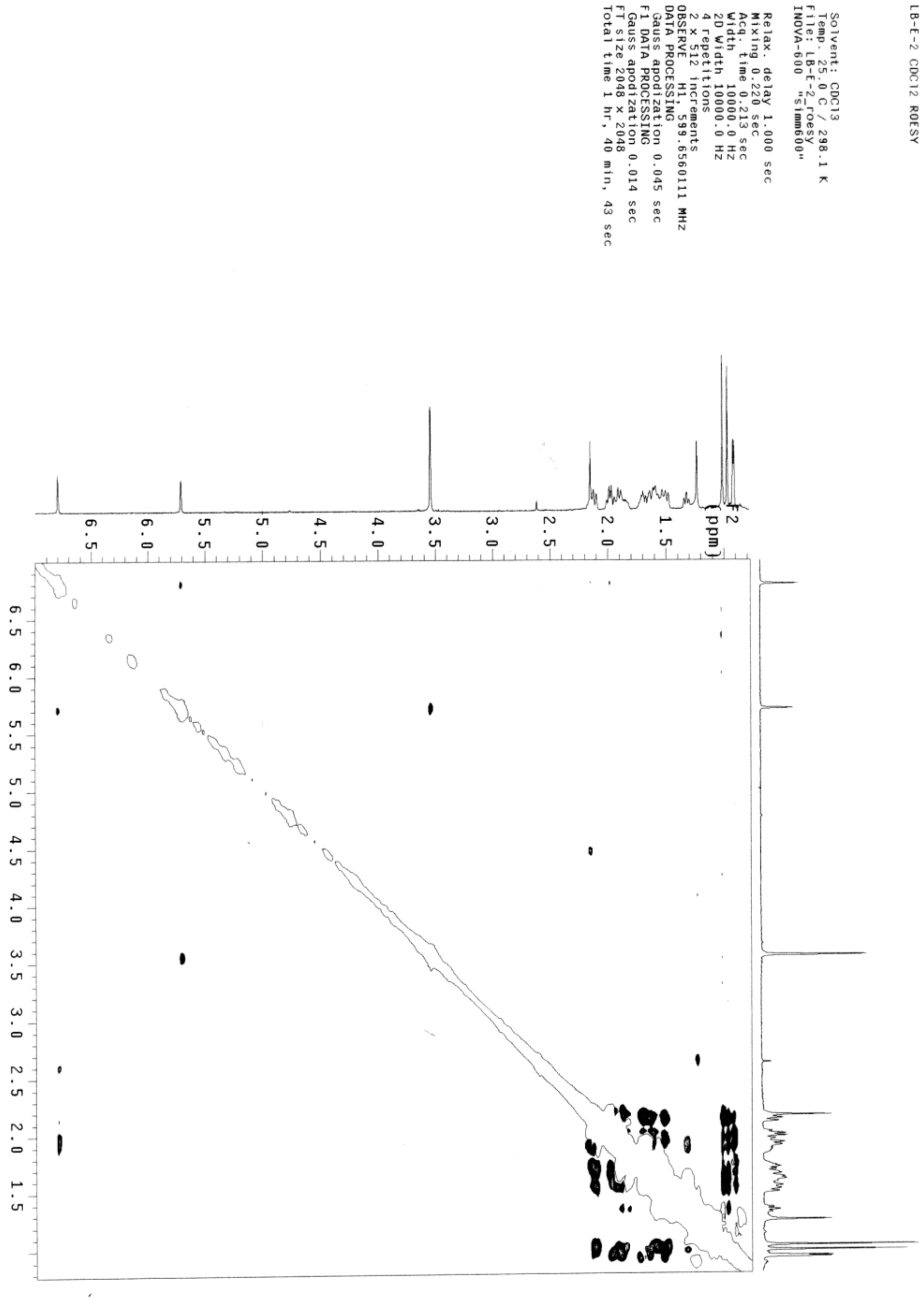

Figure S23. (+)-ESIMS spectrum of formosin C (**3**)

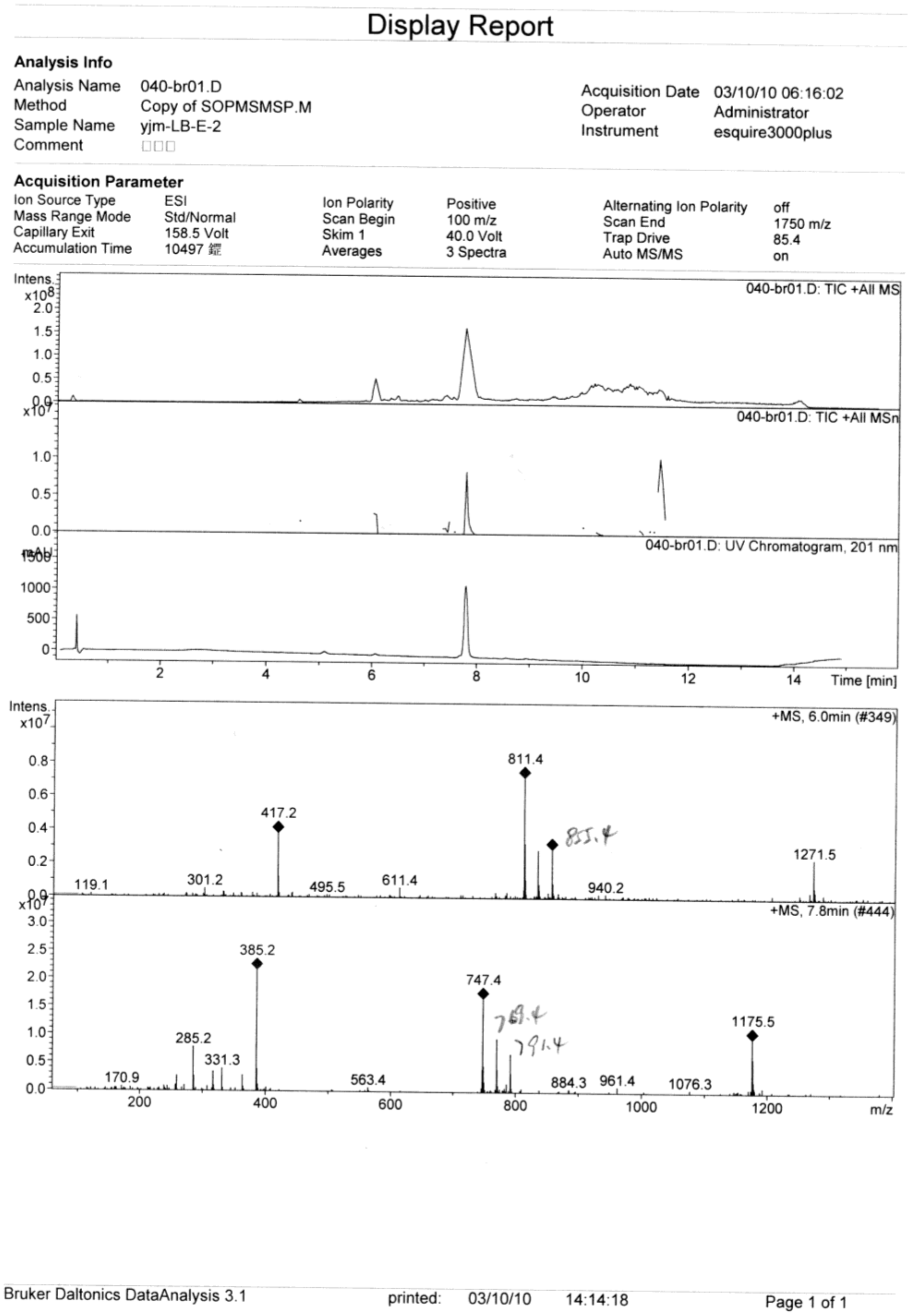

Figure S24. (−)-ESIMS spectrum of formosin C (**3**)

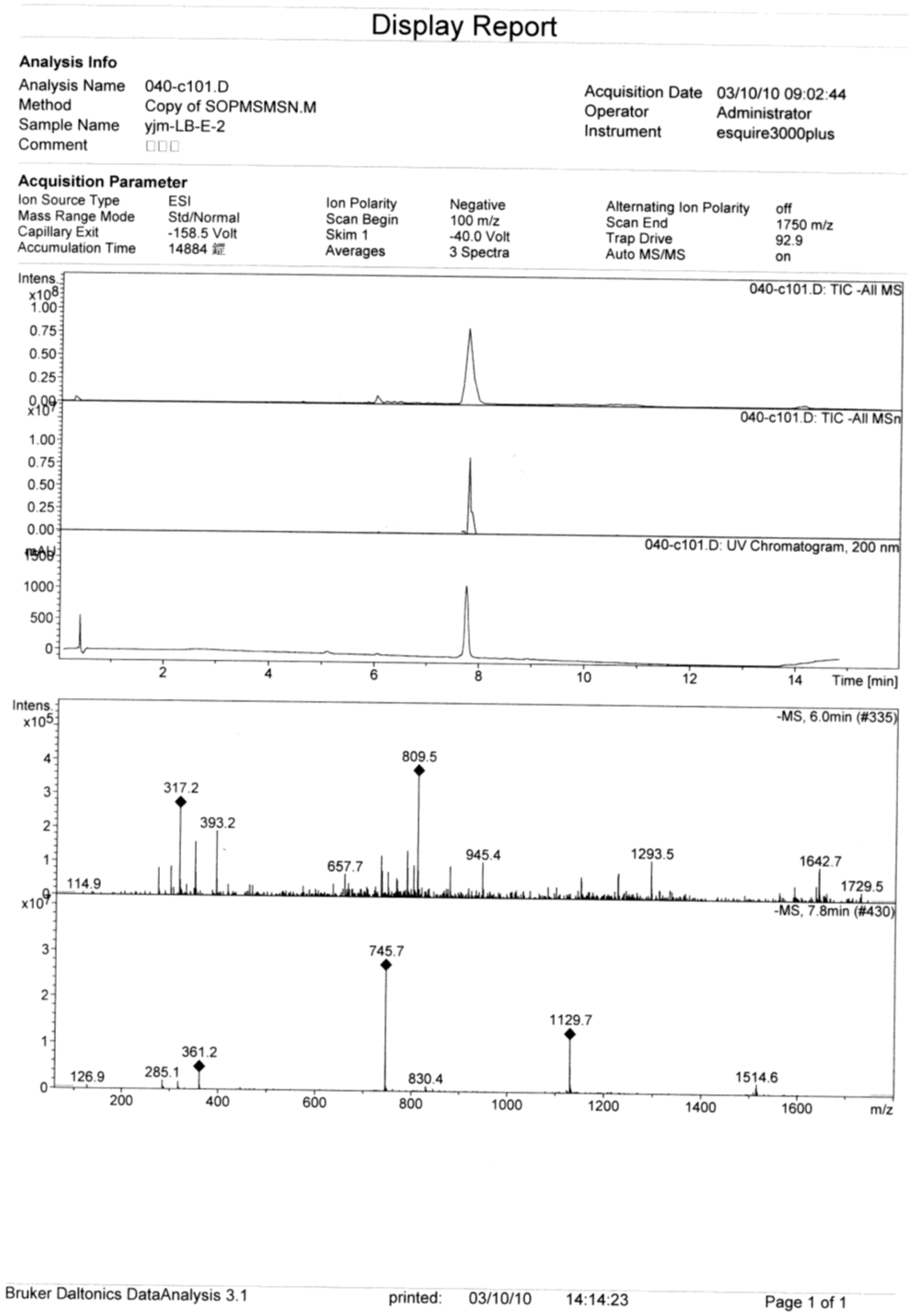

Figure S25. (+)-HRESIMS spectrum of formosin C (**3**)

Elemental Composition Report **Page 1**

Tolerance = 50.0 PPM / DBE: min = -1.5, max = 50.0
Isotope cluster parameters: Separation = 1.0 Abundance = 1.0%

Monoisotopic Mass, Odd and Even Electron Ions
13 formula(e) evaluated with 1 results within limits (up to 20 closest results for each mass)

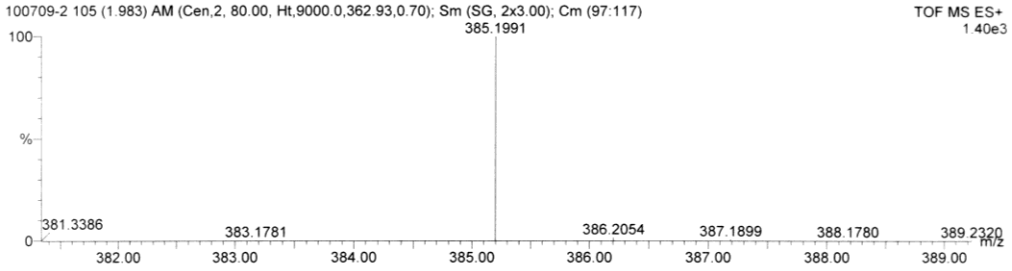

Minimum:	20.00				-1.5		
Maximum:	100.00		200.0	50.0	50.0		
Mass	RA	Calc. Mass	mDa	PPM	DBE	Score	Formula
385.1991	100.00	385.1991	0.0	0.0	6.5	1	C21 H30 O5 Na

Figure S26. IR spectrum of formosin C (**3**)

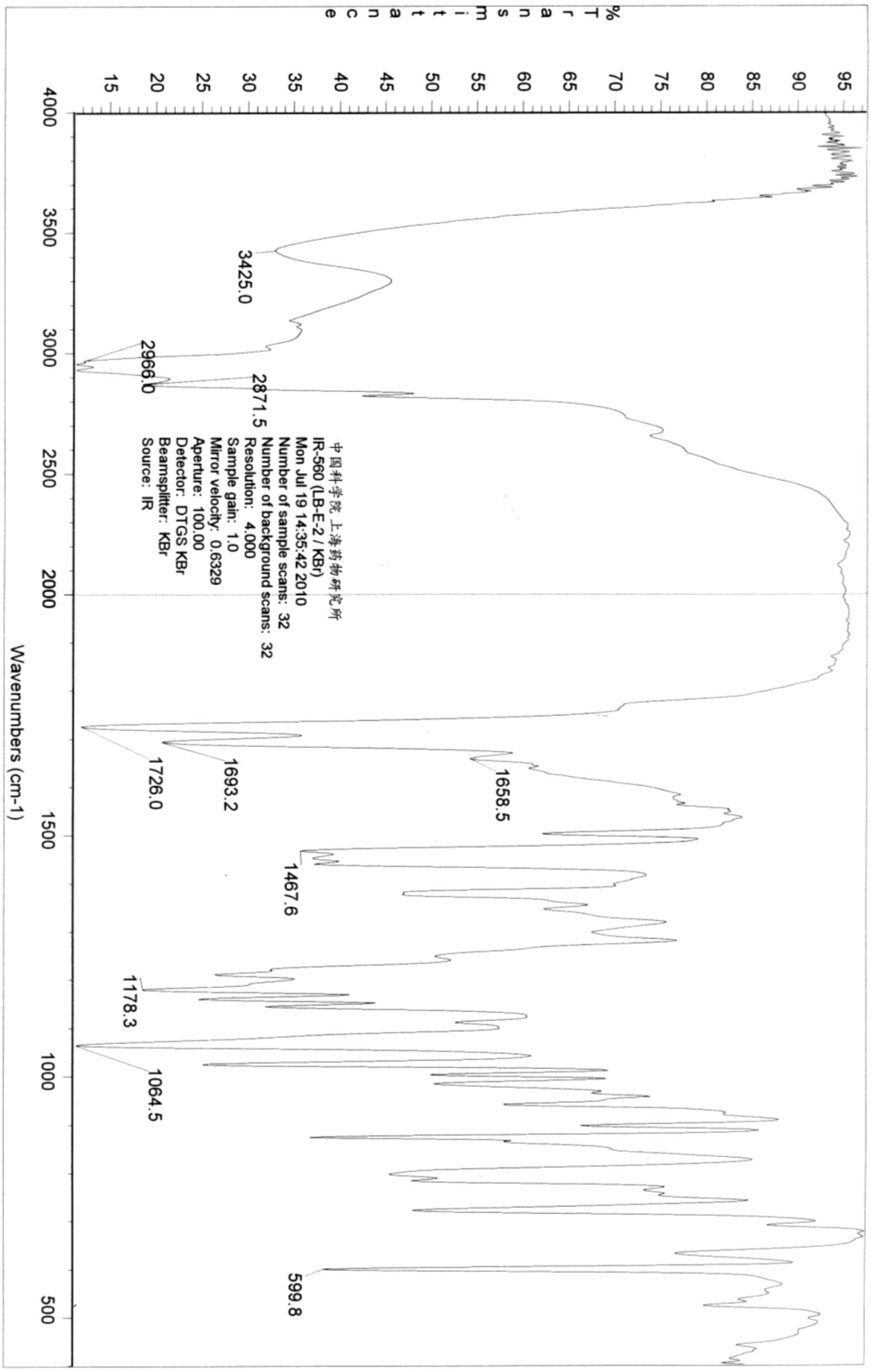

Figure S27. ^{1}H NMR spectrum of formosin D (**4**) in $CDCl_3$

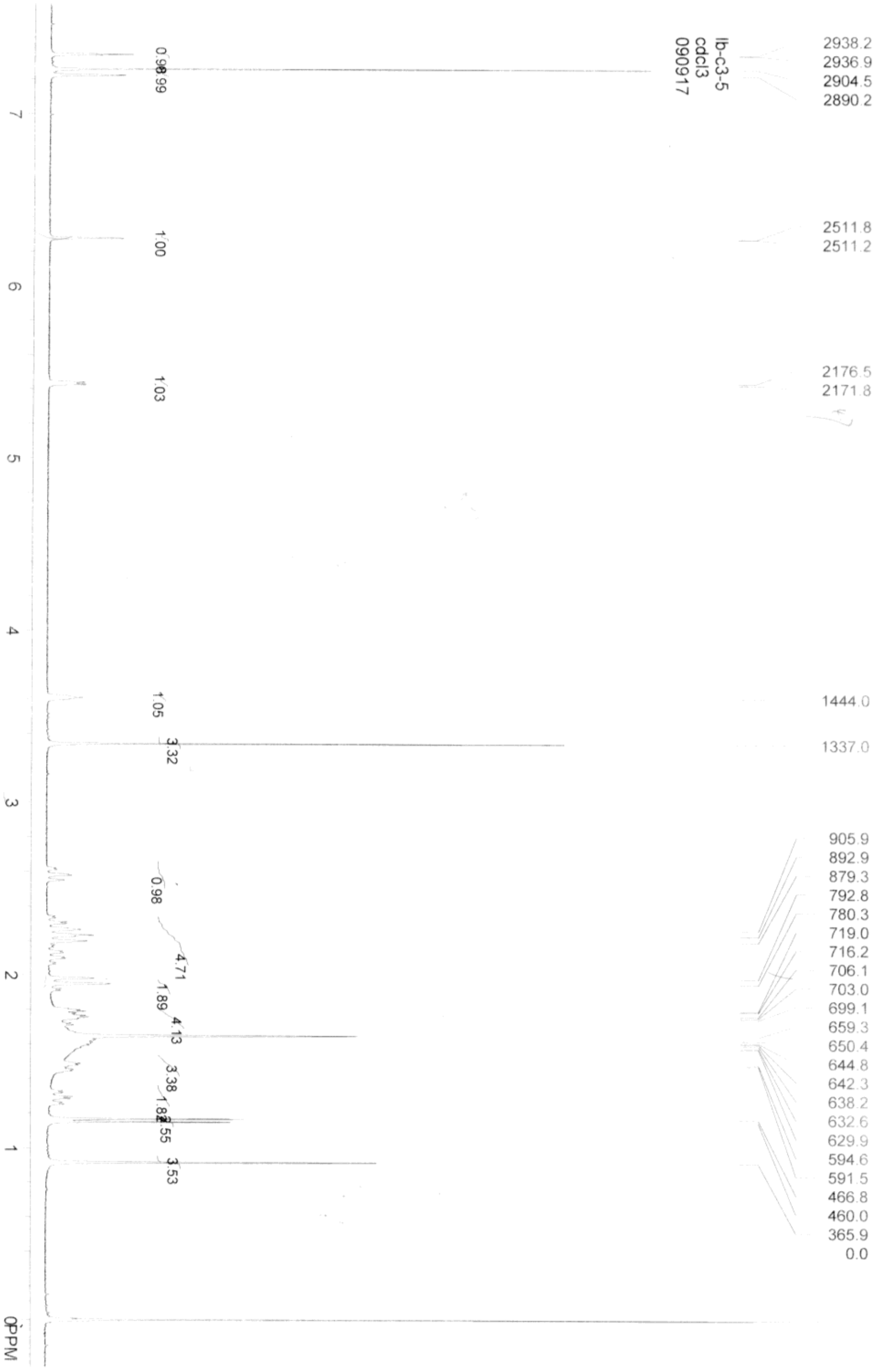

Figure S28. ^{13}C NMR spectrum of formosin D (**4**) in $CDCl_3$

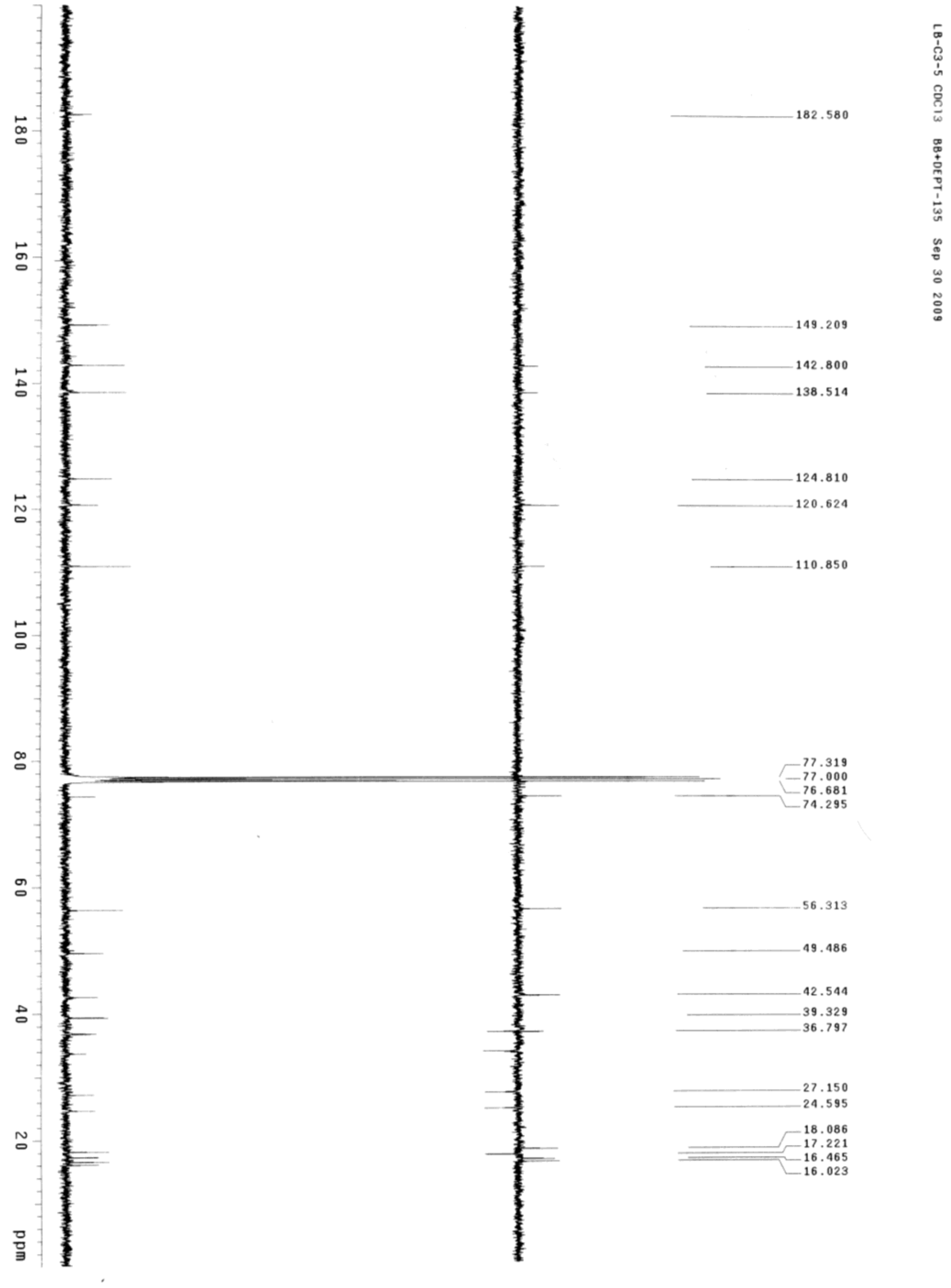

Figure S29. HSQC spectrum of formosin D (**4**)

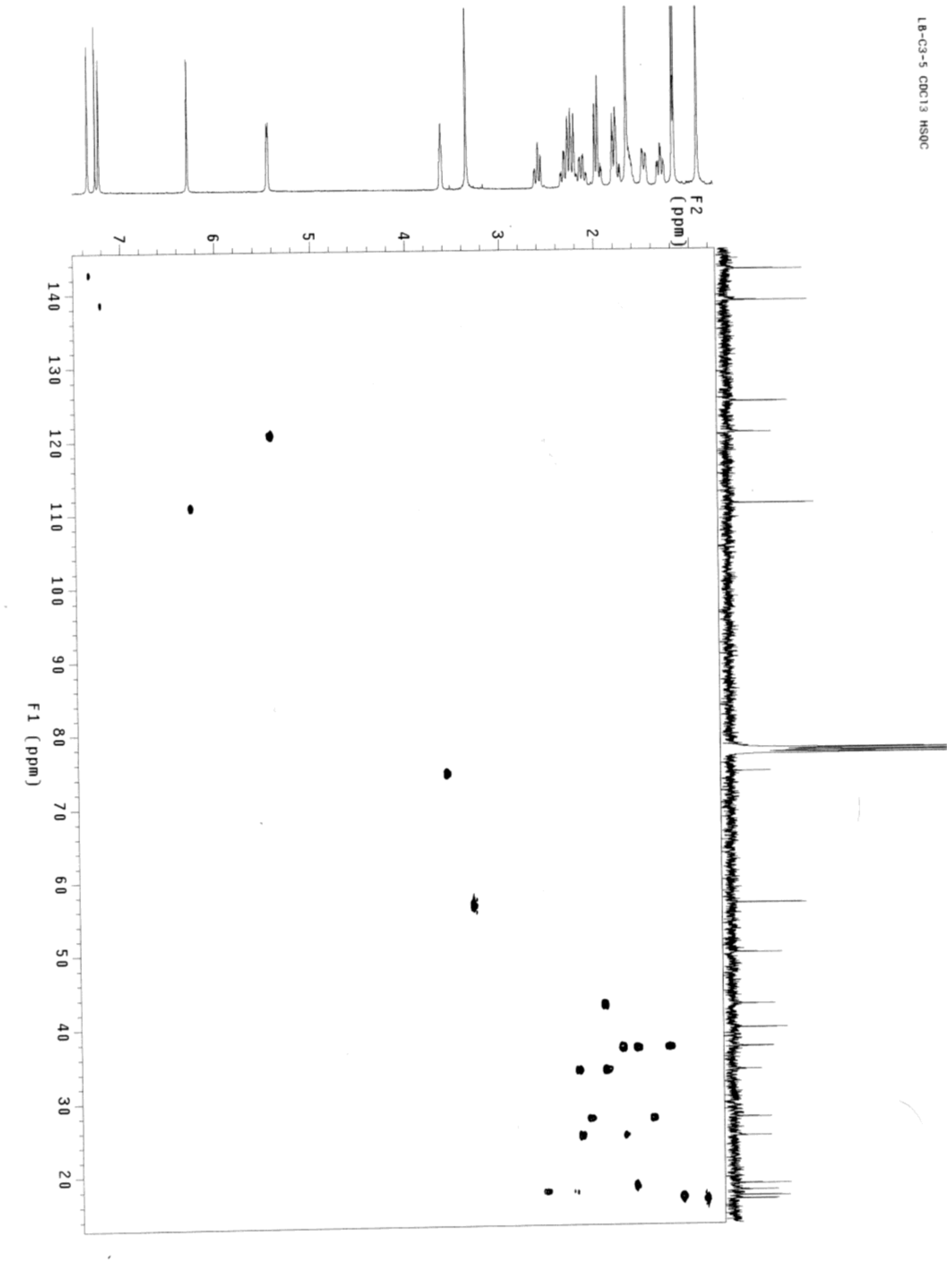

Figure S30. HMBC spectrum of formosin D (**4**)

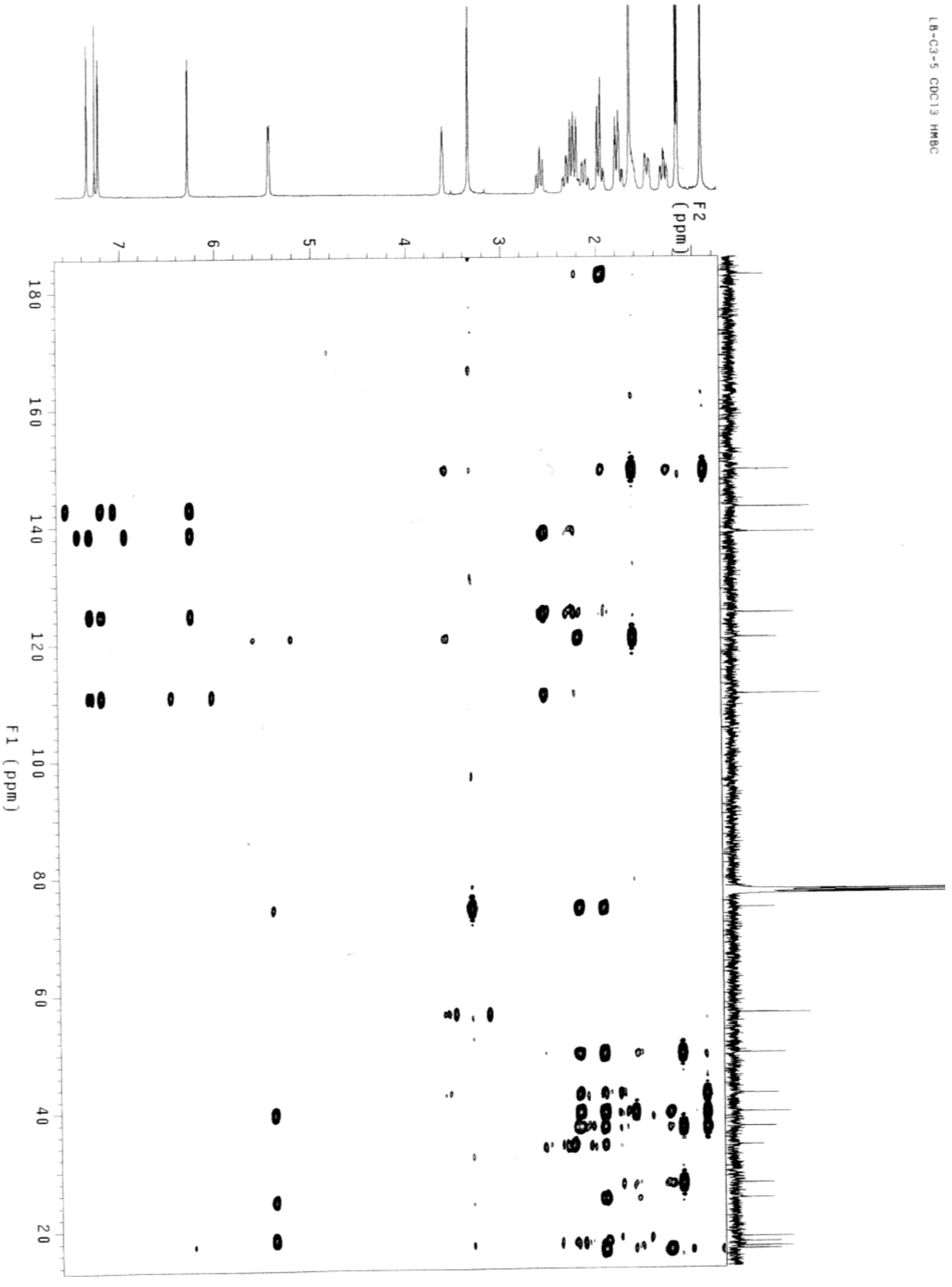

Figure S31. ROESY spectrum of formosin D (**4**)

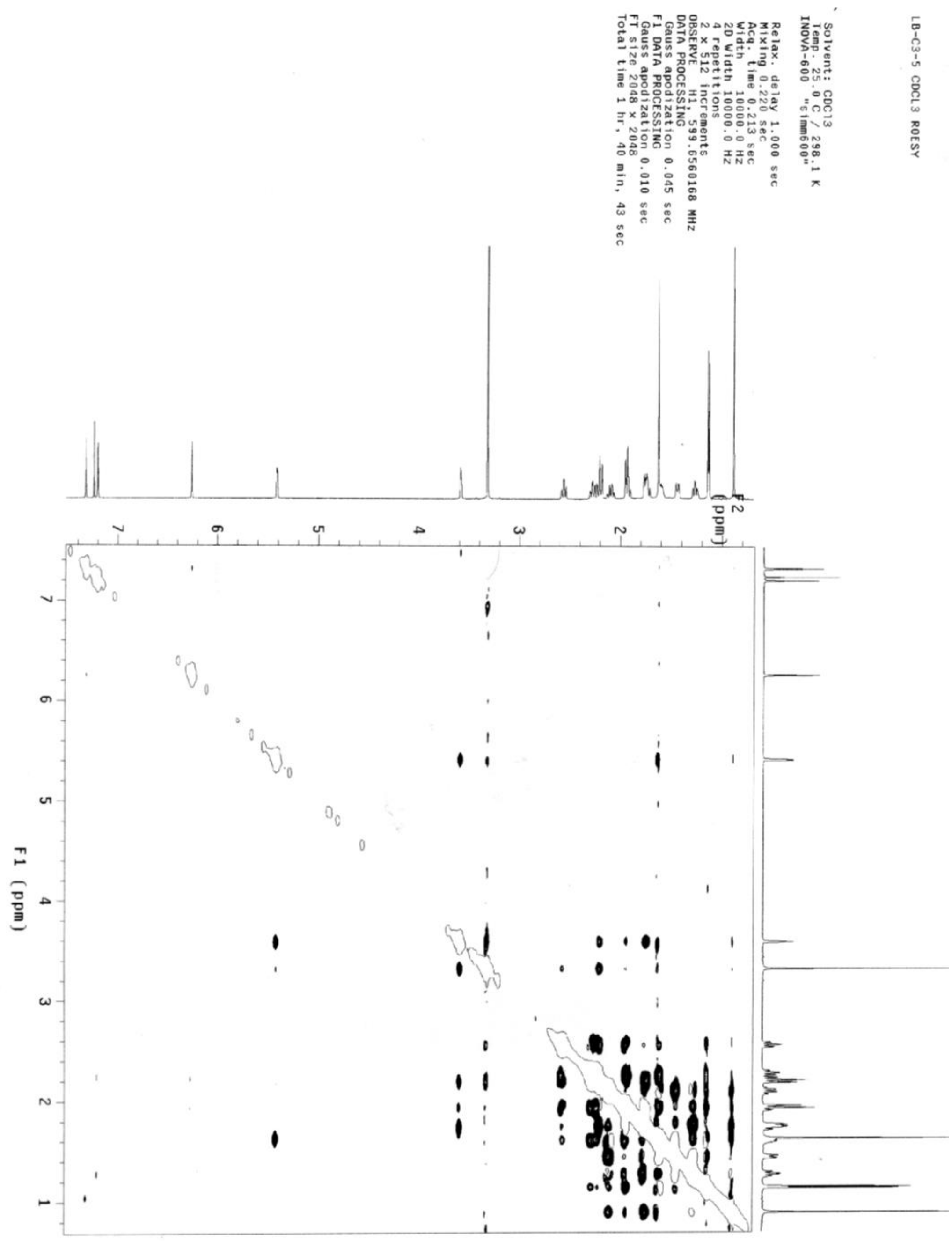

Figure S32. (+)-ESIMS spectrum of formosin D (**4**)

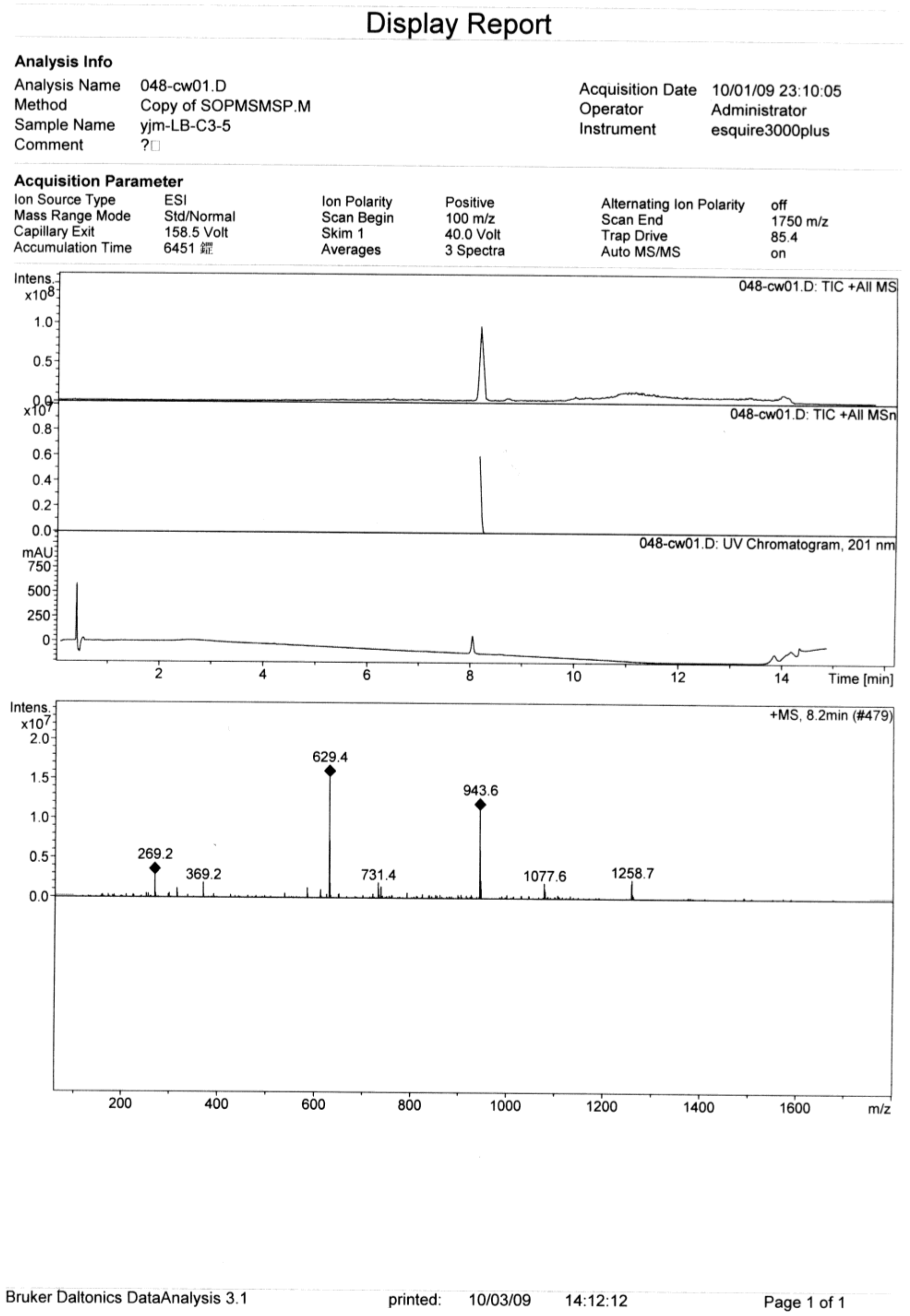

Figure S33. (−)-ESIMS spectrum of formosin D (**4**)

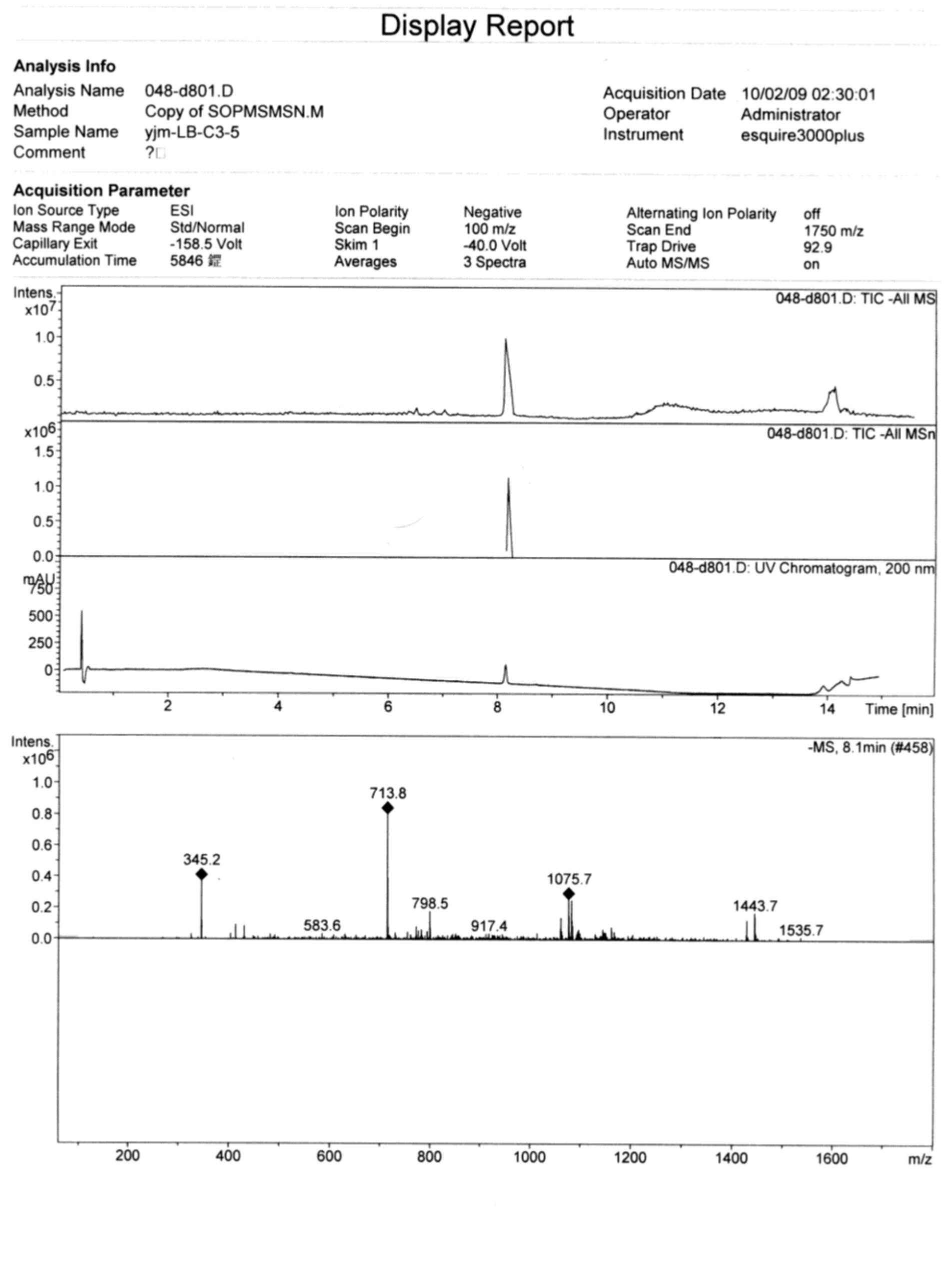

Figure S34. (+)-HRESIMS spectrum of formosin D (**4**)

Mass Spectrum SmartFormula Report

Analysis Info

Analysis Name D:\Data\FH\LB-C3-5.d
Method tune_wide.m
Sample Name LB-C3-5
Comment

Acquisition Date 6/12/2010 9:56:20 PM

Operator ECNU
Instrument / Ser# micrOTOF-Q 10198

Acquisition Parameter

Source Type	ESI	Ion Polarity	Positive	Set Nebulizer	0.4 Bar
Focus	Active	Set Capillary	4500 V	Set Dry Heater	180 °C
Scan Begin	50 m/z	Set End Plate Offset	-500 V	Set Dry Gas	4.0 l/min
Scan End	3000 m/z	Set Collision Cell RF	600.0 Vpp	Set Divert Valve	Source

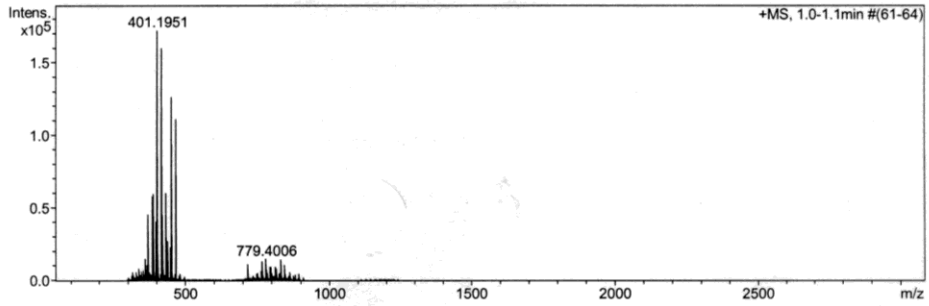

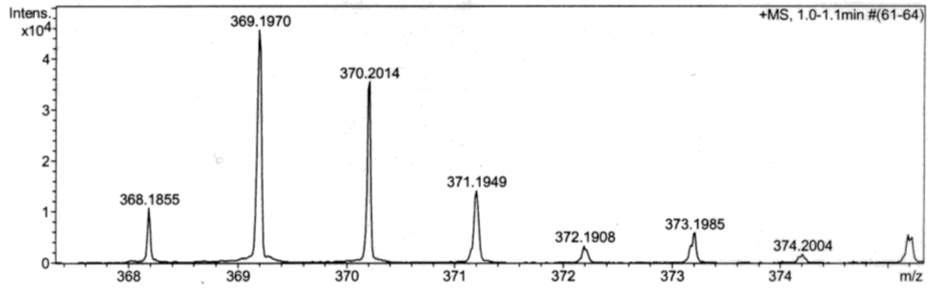

Meas. m/z	#	Formula	m/z	err [ppm]	Mean err [ppm]	rdb	N-Rule	e⁻ Conf	mSigma	Std I	Std Mean m/z	Std m/z Diff
369.1970												
	1	C 21 H 30 Na O 4	369.2036	18.1	20.5	6.5	ok	even	355.49	0.3884	0.0083	0.0011

Bruker Compass DataAnalysis 4.0 printed: 6/12/2010 10:06:35 PM Page 1 of 1

Figure S35. IR spectrum of formosin D (**4**)

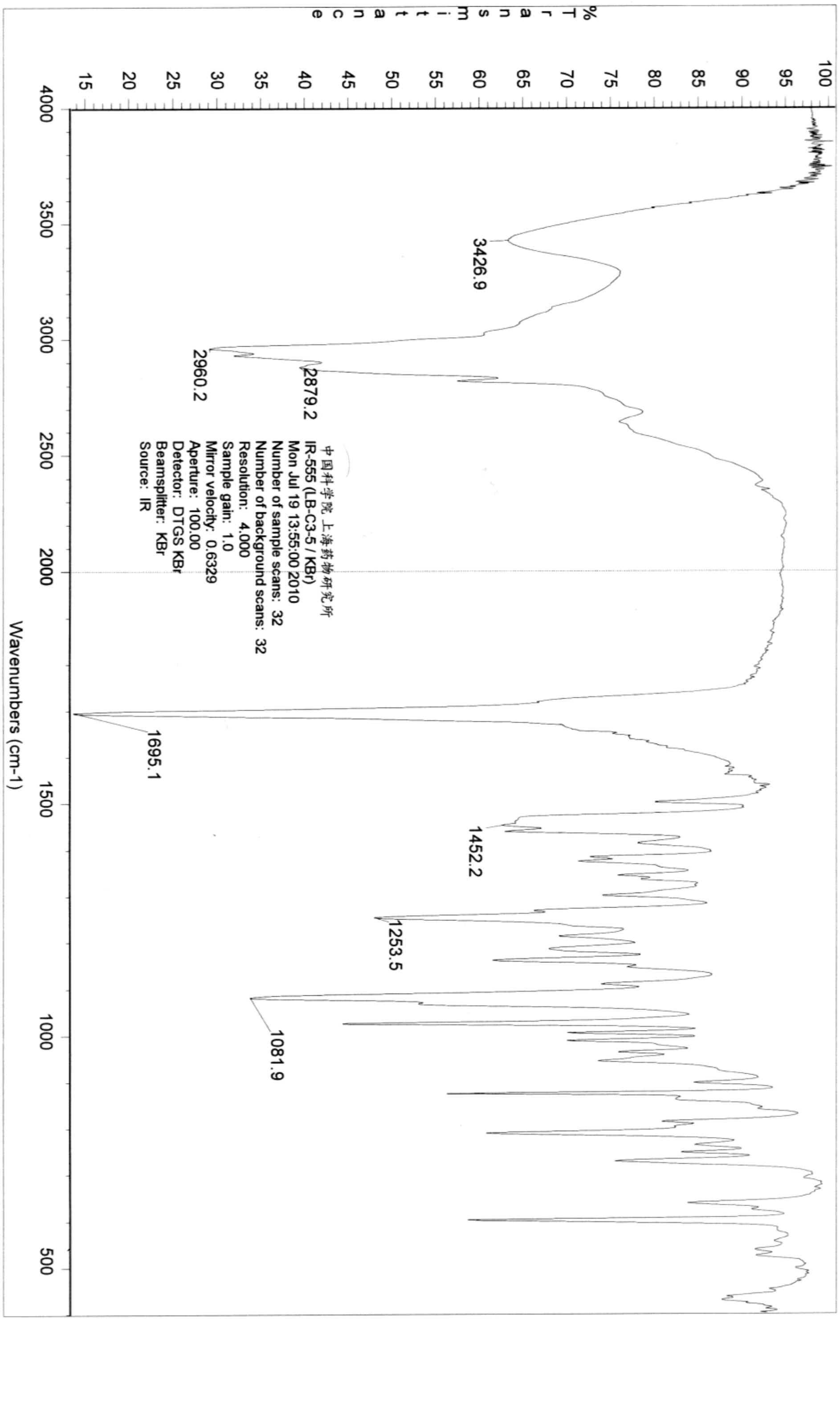

Figure S36. ^{1}H NMR spectrum of formosin E (**5**) in $CDCl_3$

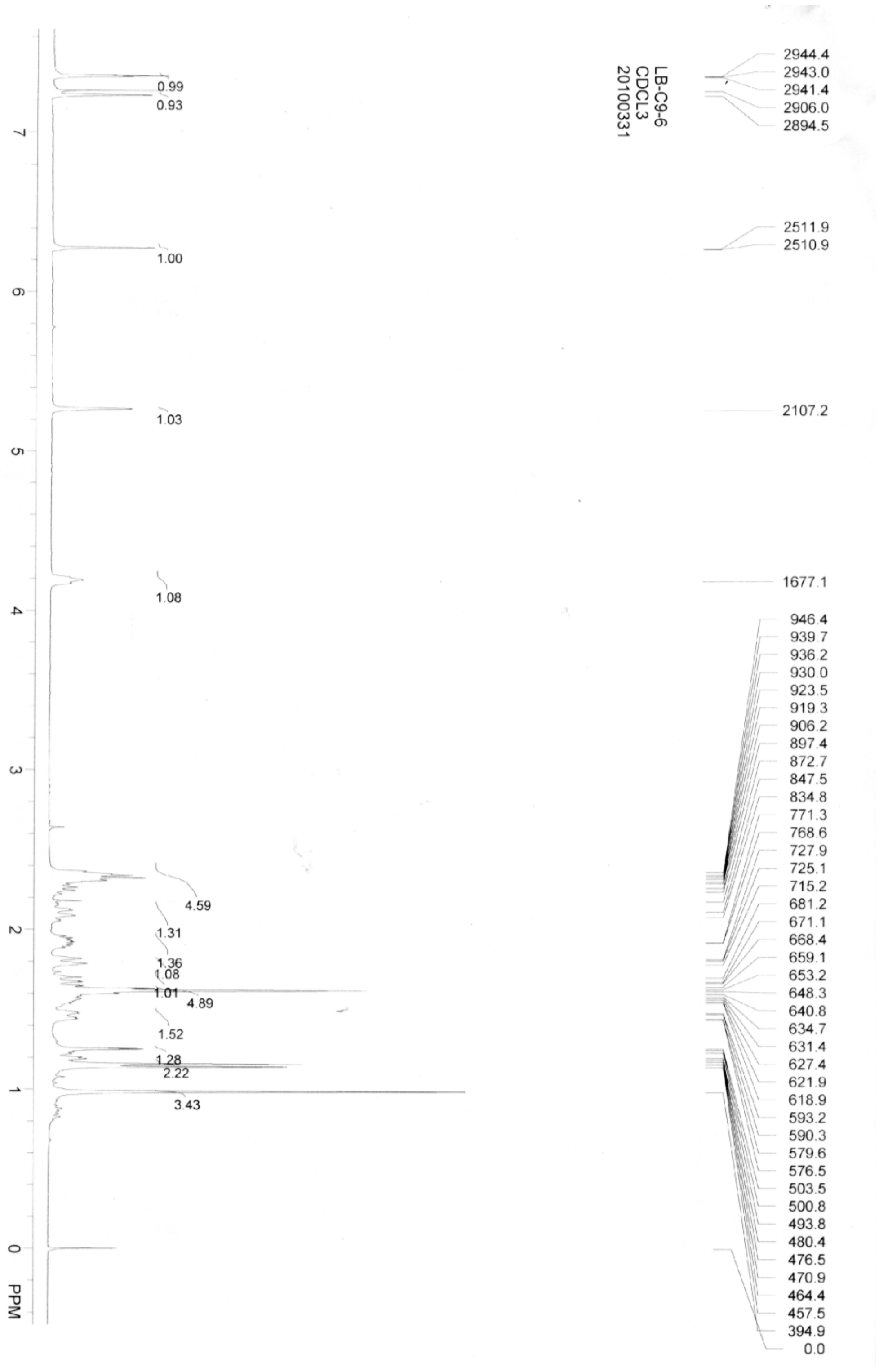

Figure S37. ^{13}C NMR spectrum of formosin E (**5**) in $CDCl_3$

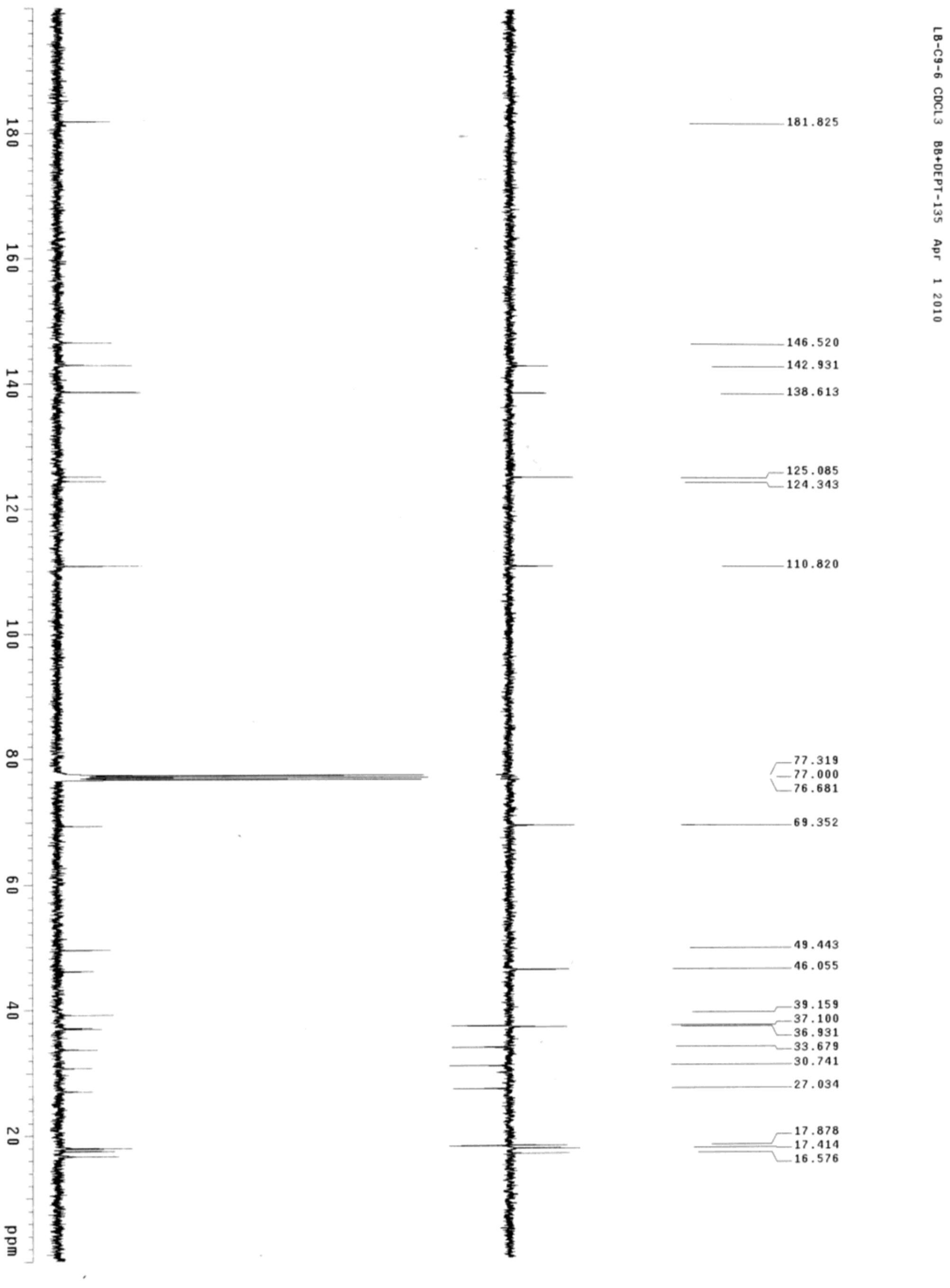

Figure S38. HSQC spectrum of formosin E (**5**) in $CDCl_3$

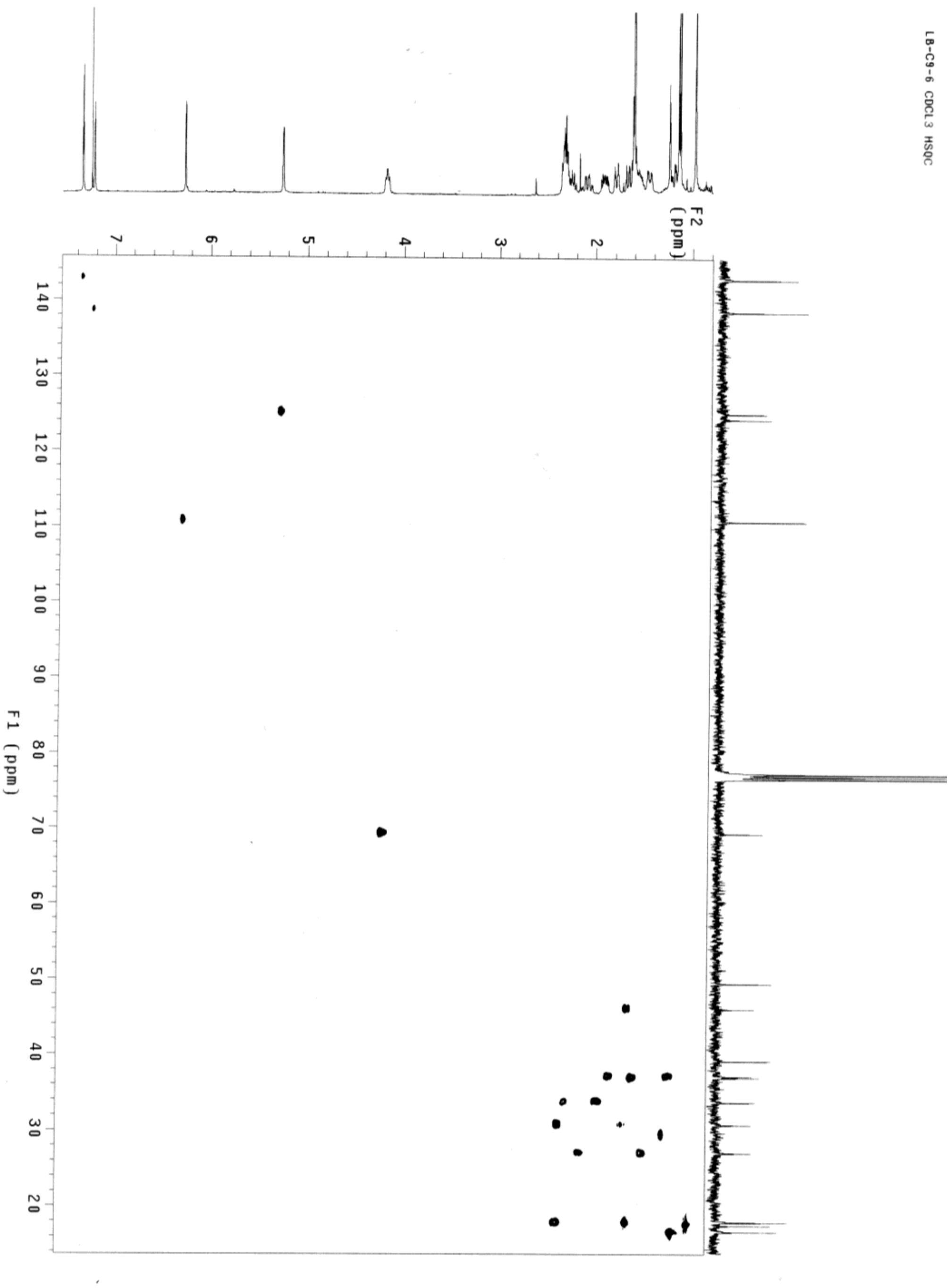

Figure S39. HMBC spectrum of formosin E (**5**) in $CDCl_3$

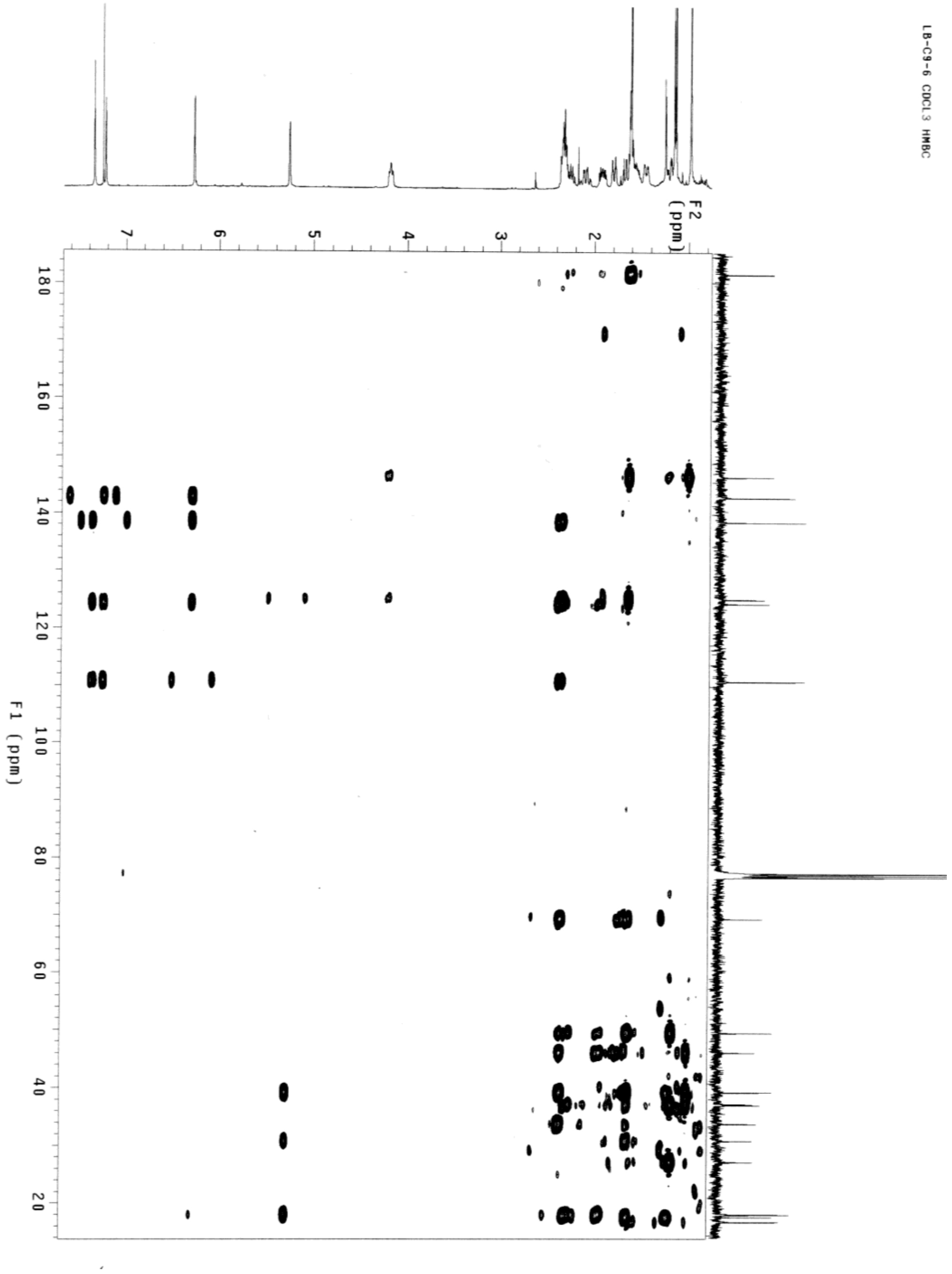

Figure S40. ROESY spectrum of formosin E (**5**) in $CDCl_3$

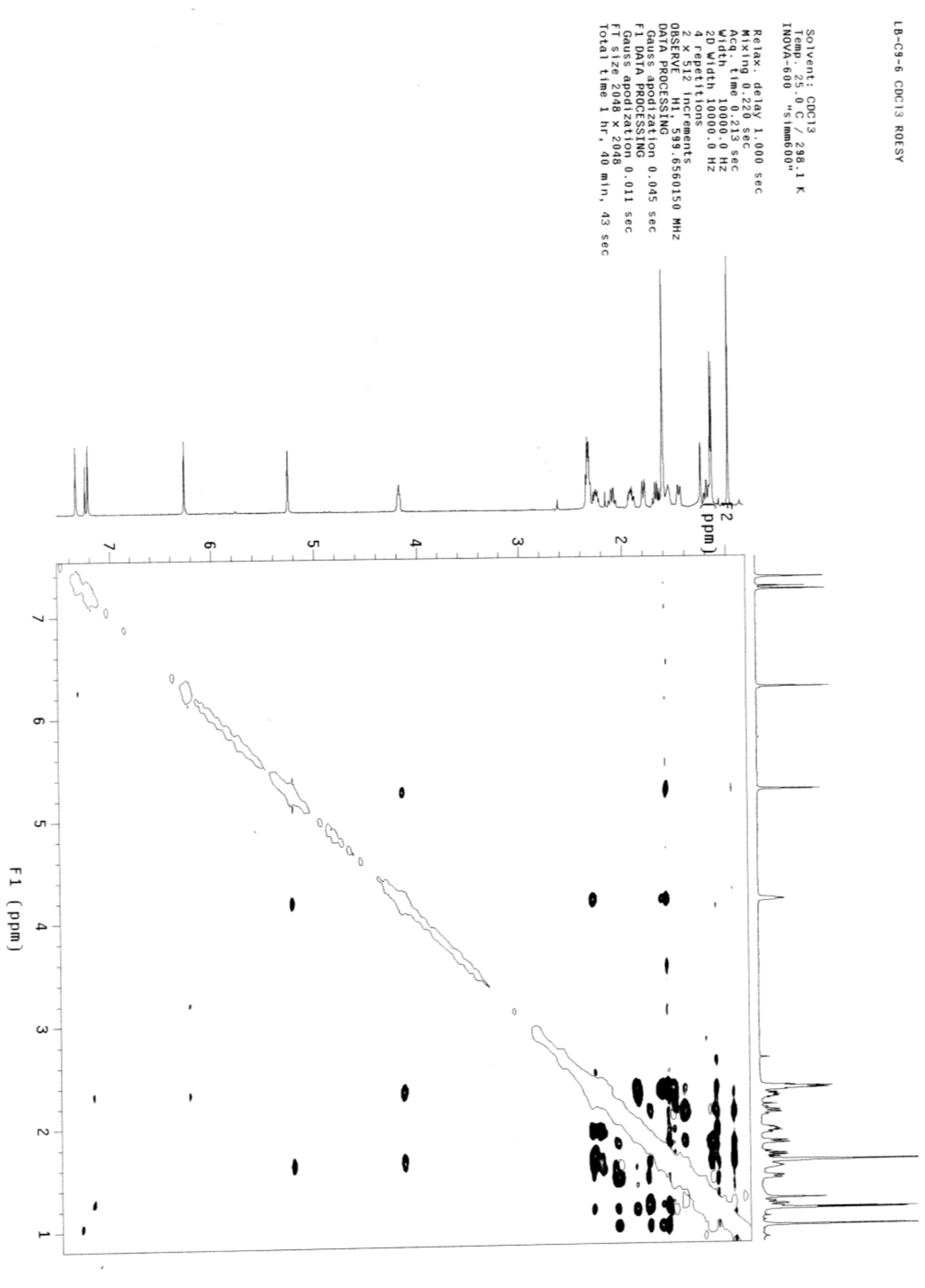

Figure S41. (+)-ESIMS spectrum of formosin E (**5**)

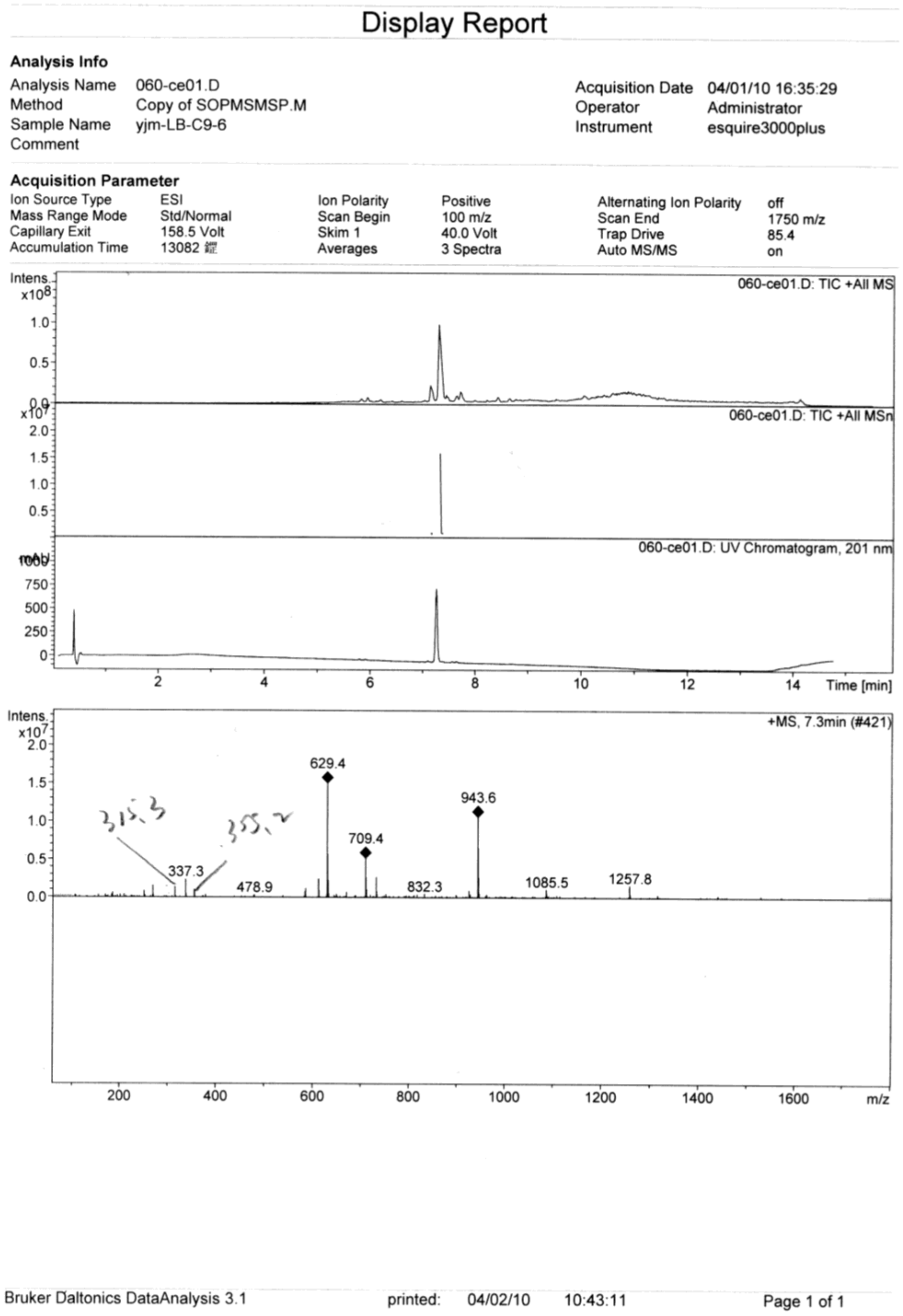

Figure S42. (−)-ESIMS spectrum of formosin E (**5**)

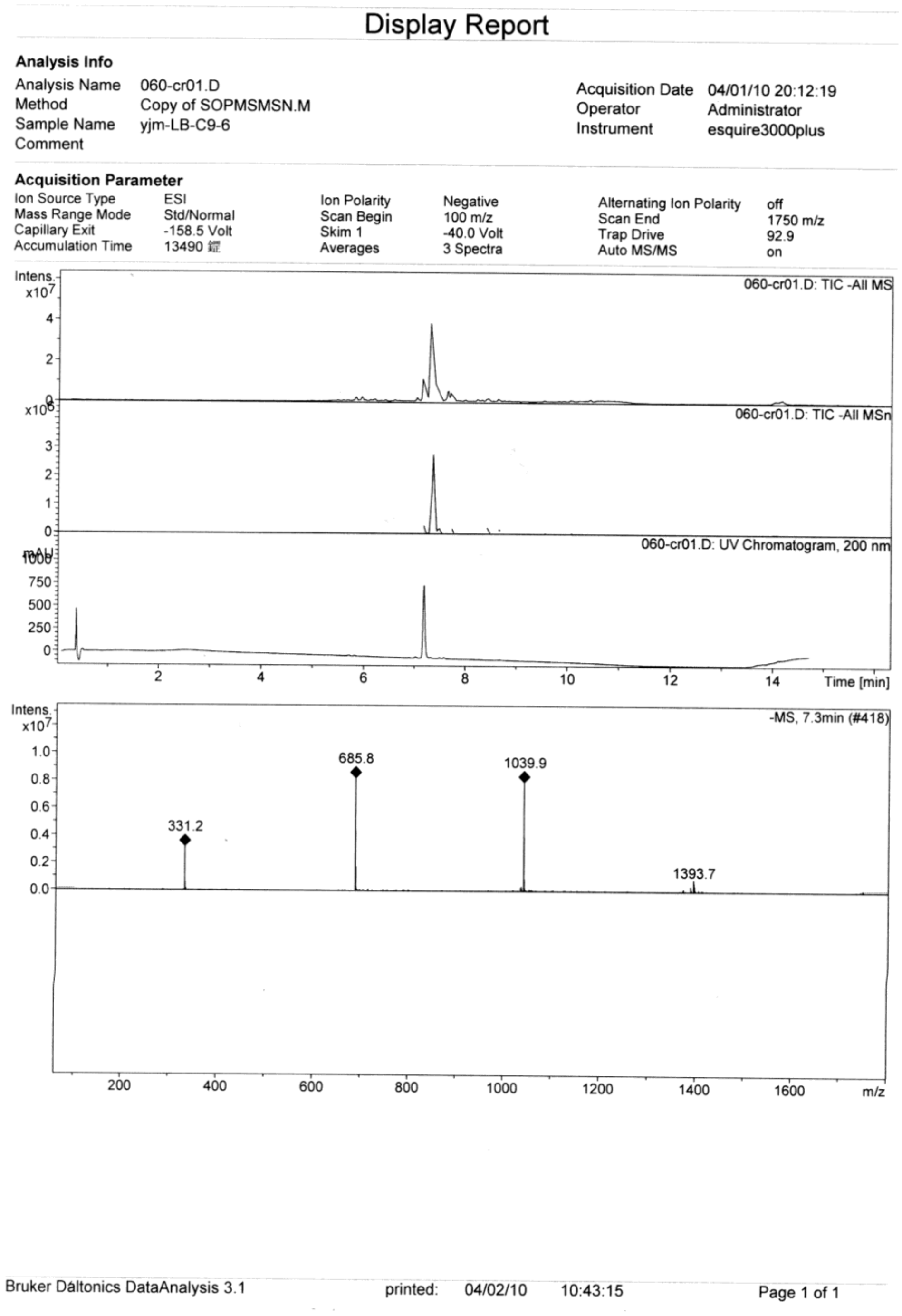

Figure S43. (−)-HRESIMS spectrum of formosin E (**5**)

Elemental Composition Report **Page 1**

Tolerance = 50.0 PPM / DBE: min = -1.5, max = 50.0
Isotope cluster parameters: Separation = 1.0 Abundance = 1.0%

Monoisotopic Mass, Odd and Even Electron Ions
10 formula(e) evaluated with 1 results within limits (up to 20 closest results for each mass)

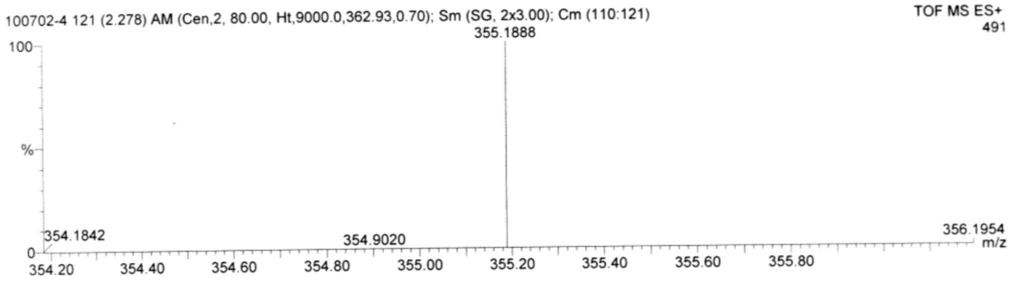

Minimum:	20.00				-1.5		
Maximum:	100.00		200.0	50.0	50.0		
Mass	RA	Calc. Mass	mDa	PPM	DBE	Score	Formula
355.1888	100.00	355.1885	0.3	0.8	6.5	1	C20 H28 O4 Na

Figure S44. IR spectrum of formosin E (**5**)

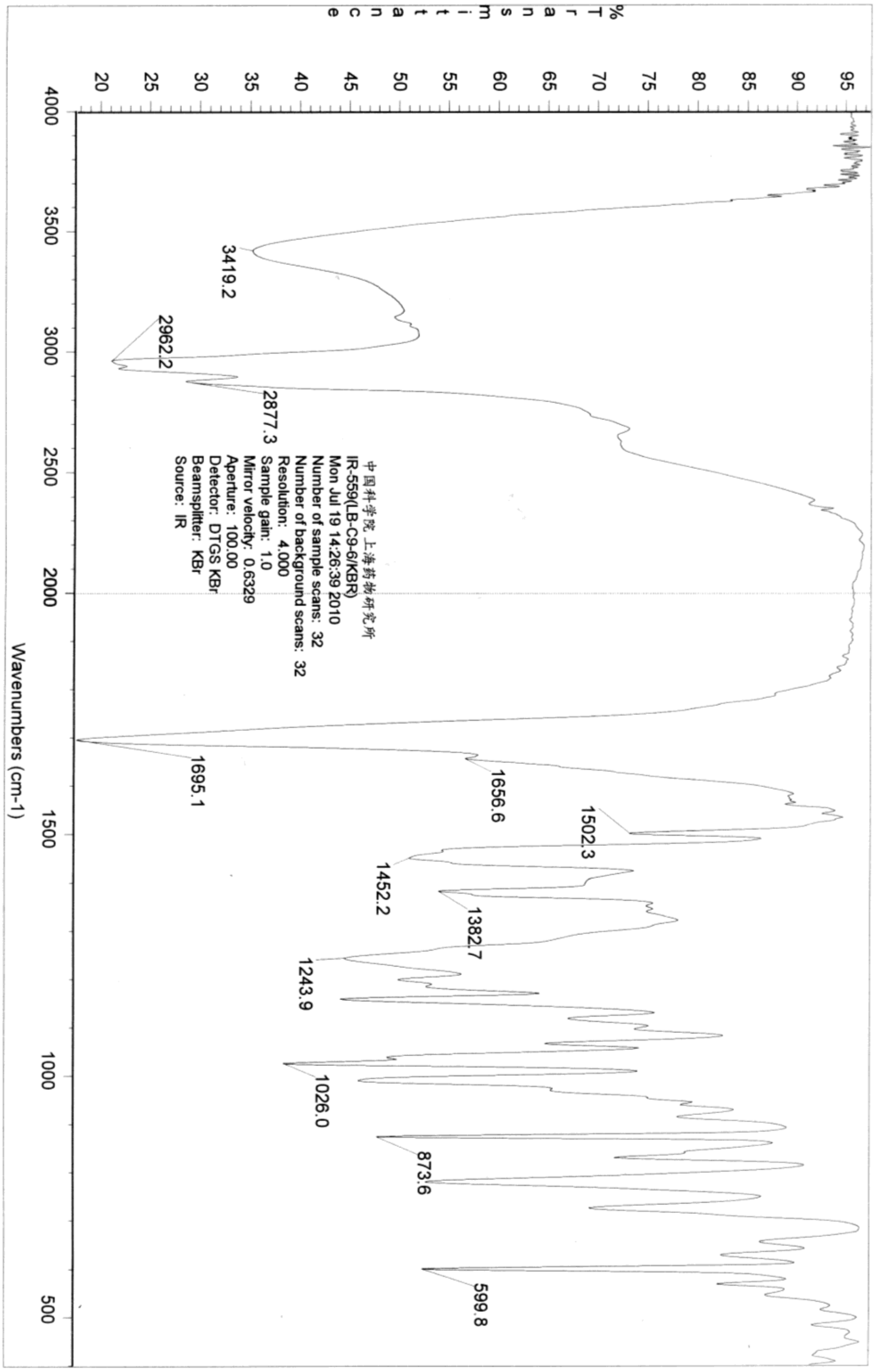

Figure S45. ^{1}H NMR spectrum of formosin F (**6**) in $CDCl_3$

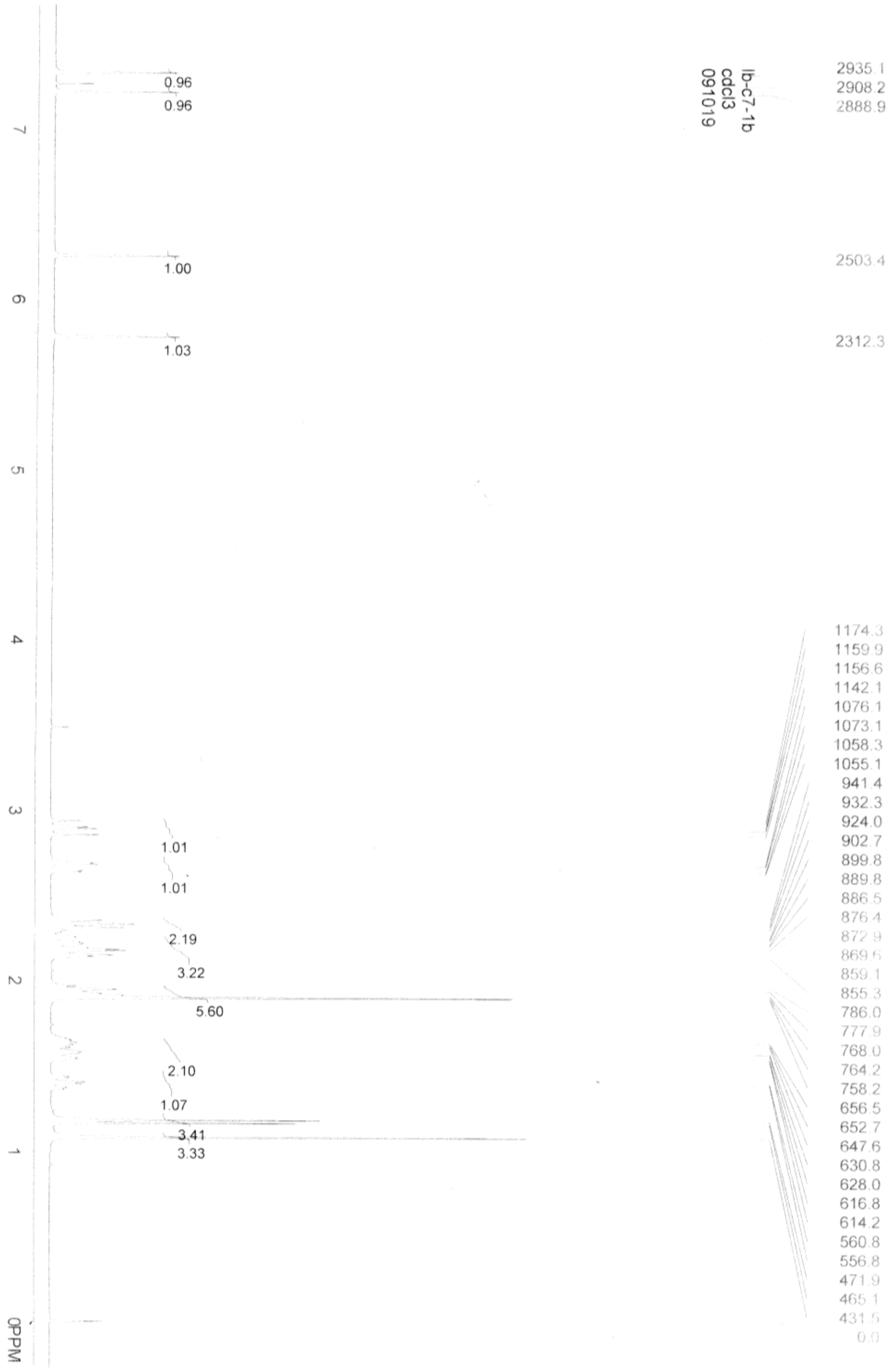

Figure S46. ^{13}C NMR spectrum of formosin F (**6**) in $CDCl_3$

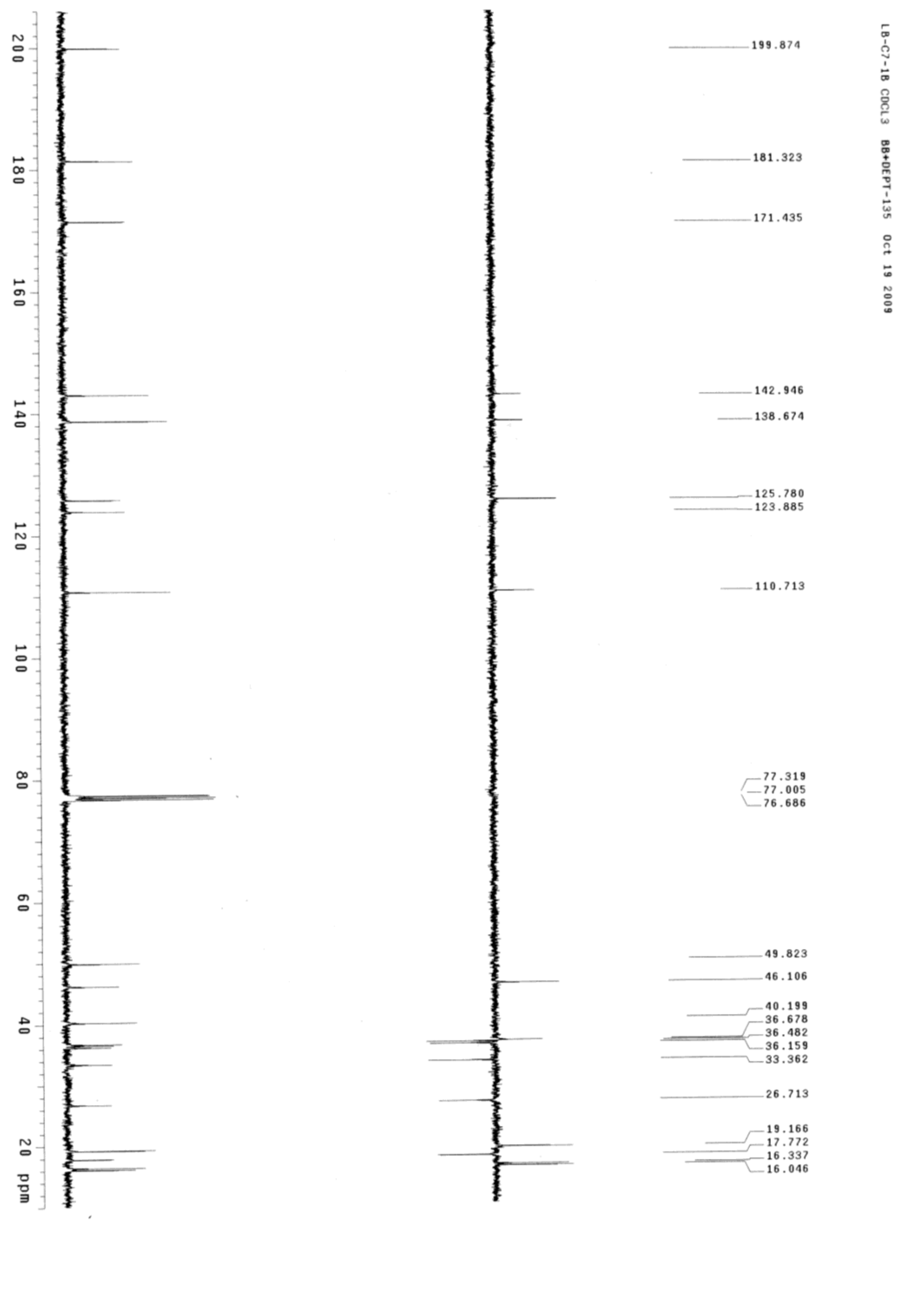

Figure S47. HSQC spectrum of formosin F (**6**) in $CDCl_3$

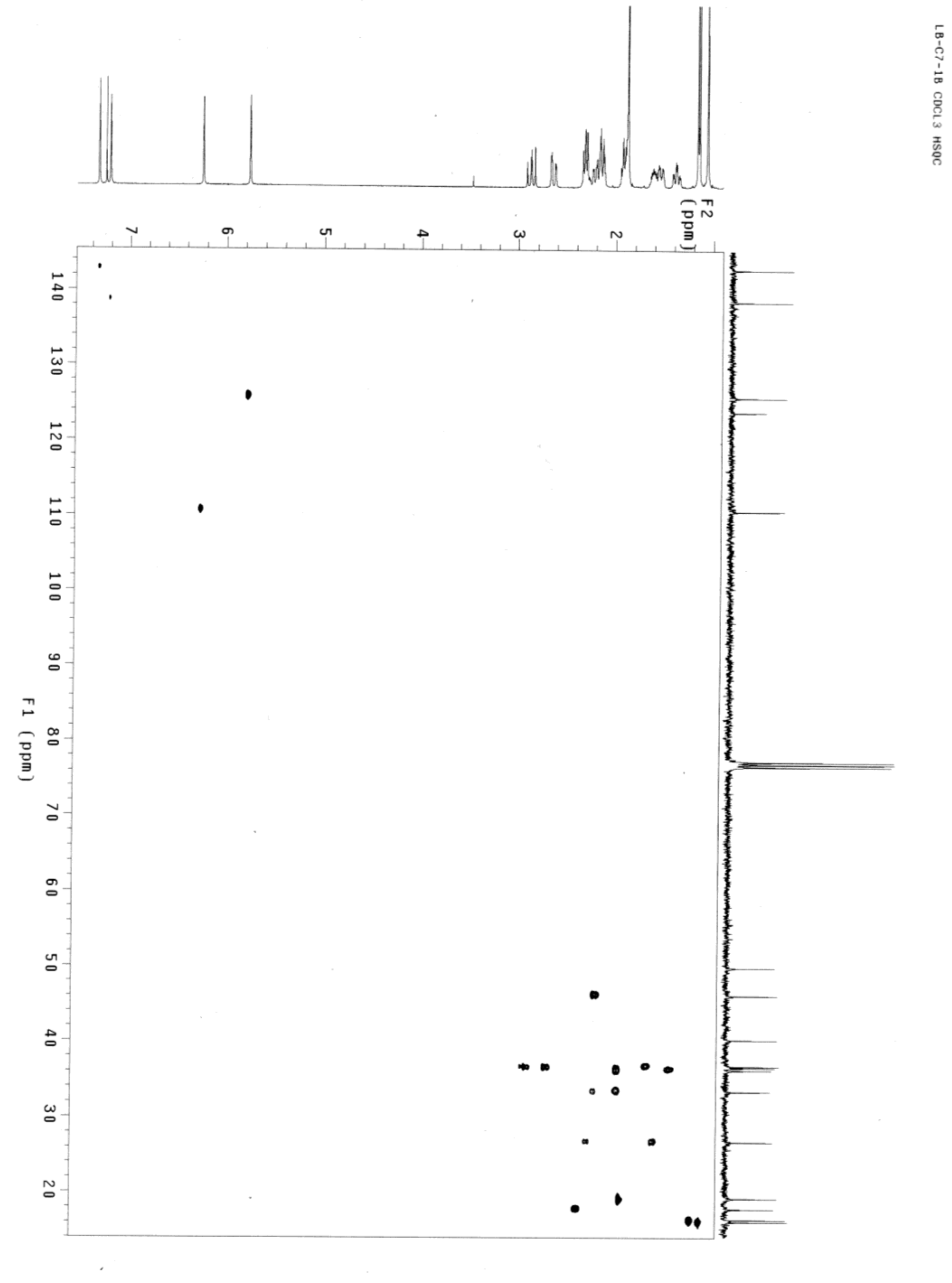

Figure S48. HMBC spectrum of formosin F (**6**) in $CDCl_3$

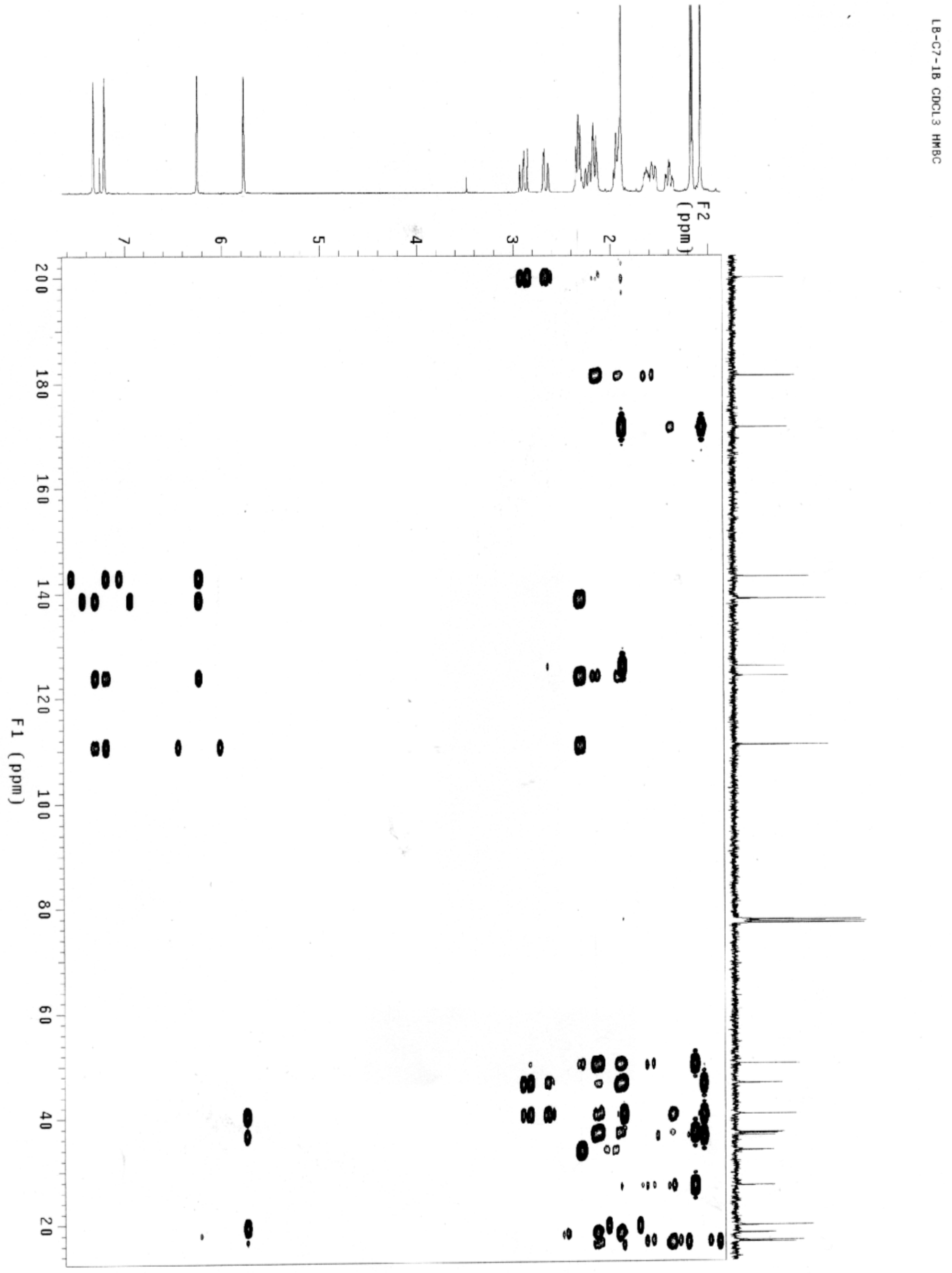

Figure S49. (+)-ESIMS spectrum of formosin F (**6**)

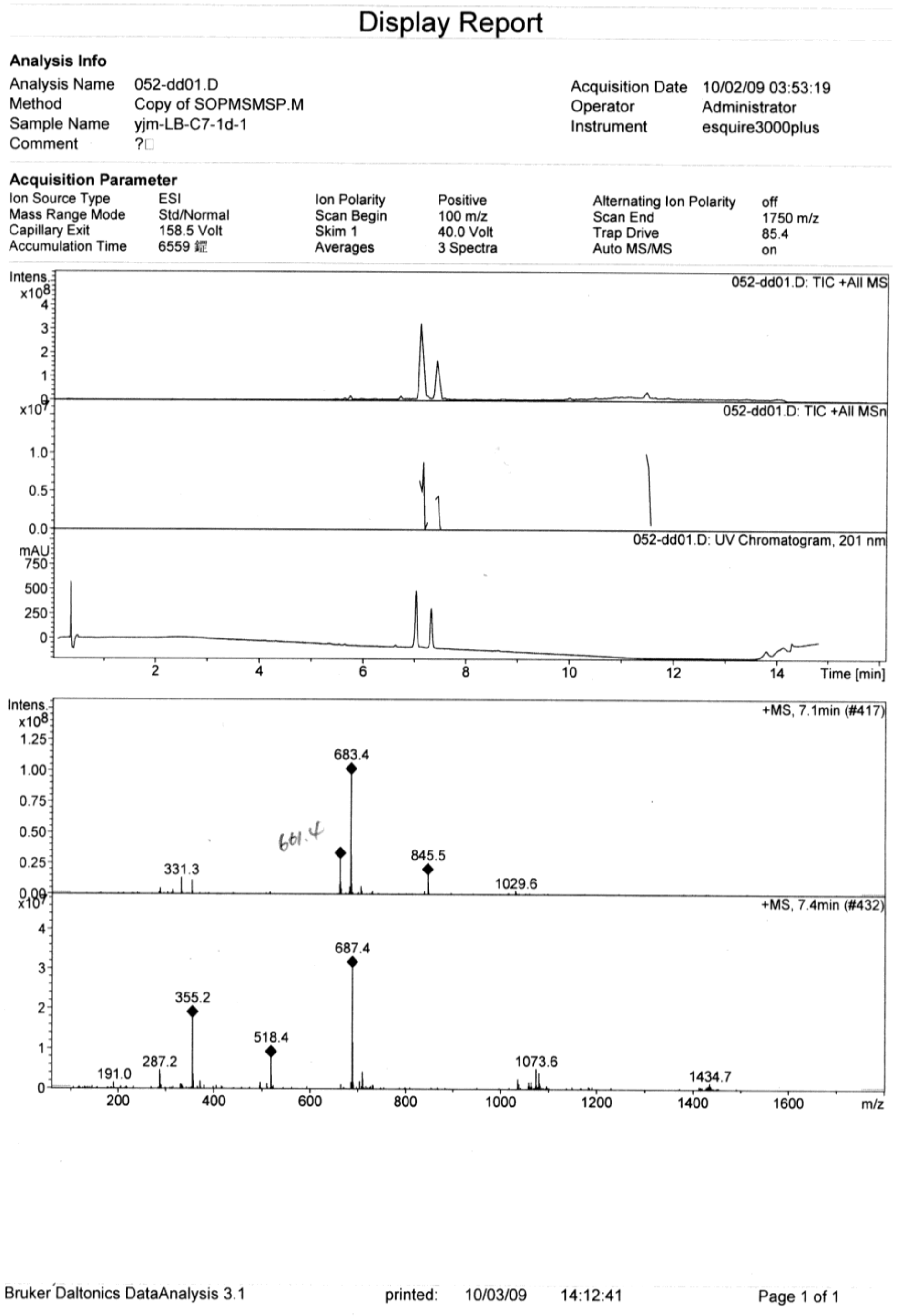

Figure S50.(−)-ESIMS spectrum of formosin F (**6**)

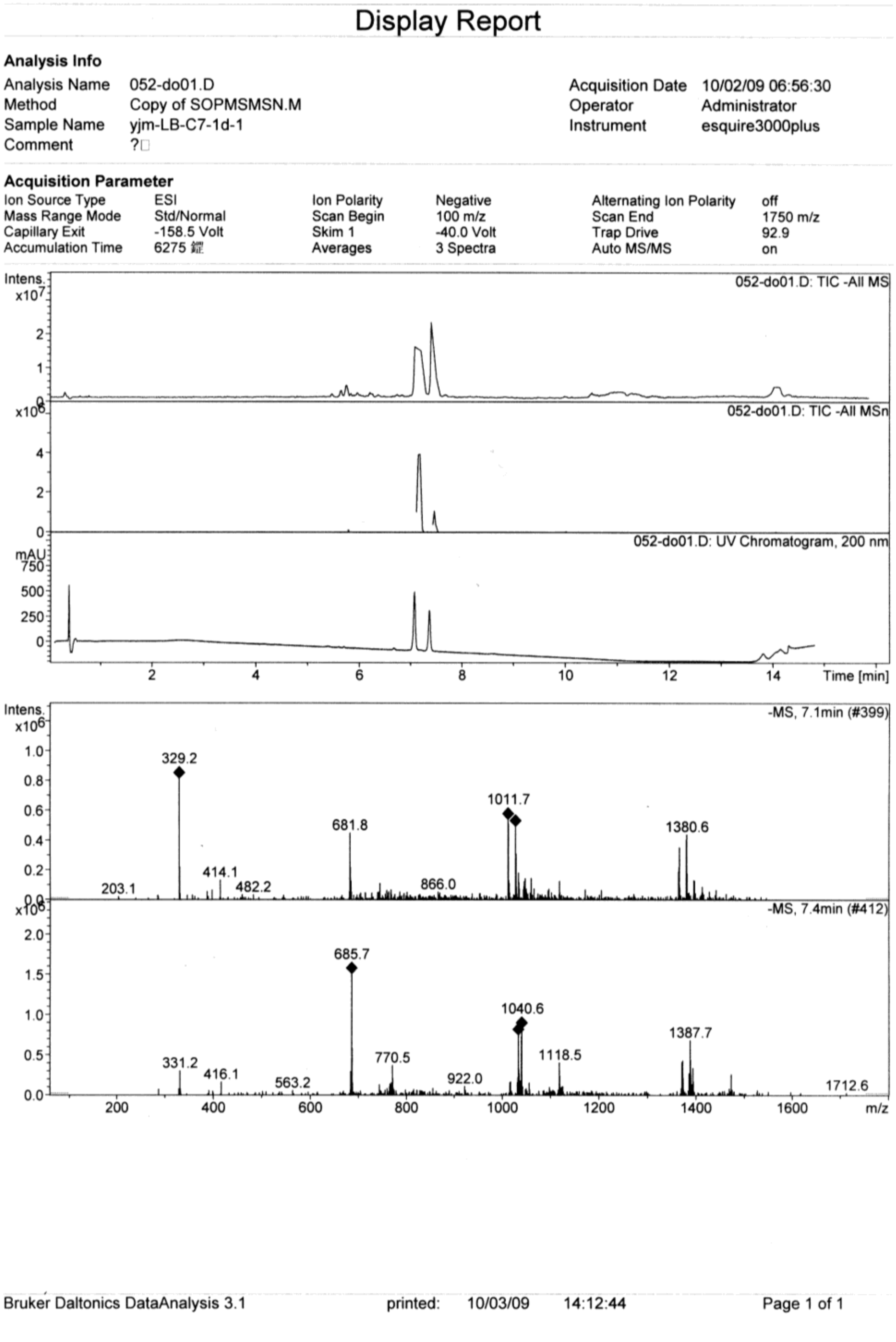

Figure S51. (+)-HRESIMS spectrum of formosin F (**6**)

Mass Spectrum SmartFormula Report

Analysis Info

Analysis Name D:\Data\YUE\LB-C7-1B.d
Method tune_low.m
Sample Name LB-C7-1B
Comment

Acquisition Date 6/7/2010 5:48:56 PM
Operator ECNU
Instrument / Ser# micrOTOF-Q 10198

Acquisition Parameter

Source Type	ESI	Ion Polarity	Positive	Set Nebulizer	0.4 Bar
Focus	Active	Set Capillary	4500 V	Set Dry Heater	180 °C
Scan Begin	50 m/z	Set End Plate Offset	-500 V	Set Dry Gas	4.0 l/min
Scan End	3000 m/z	Set Collision Cell RF	150.0 Vpp	Set Divert Valve	Waste

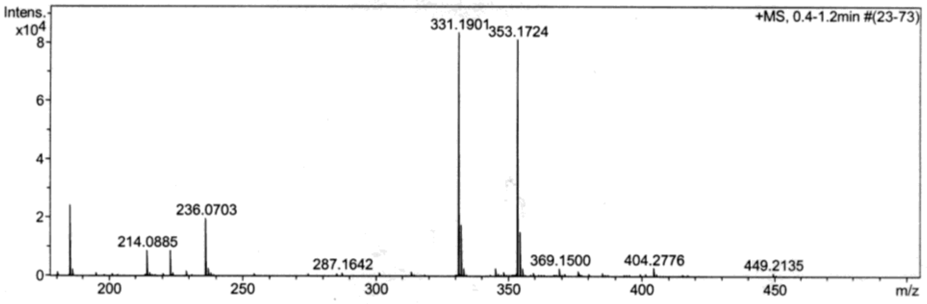

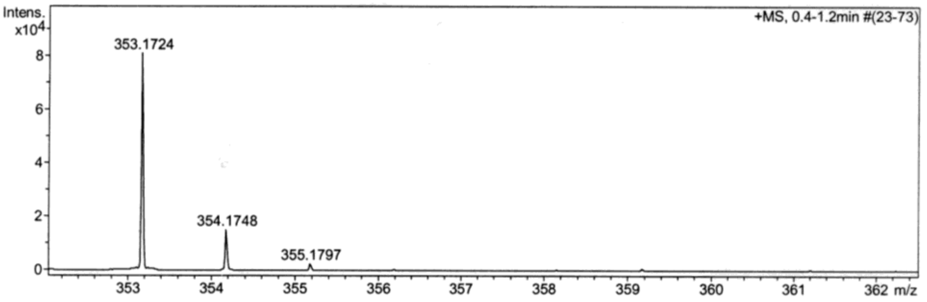

Meas. m/z	#	Formula	m/z	err [ppm]	Mean err [ppm]	N-Rule	e⁻ Conf	mSigma	Std I	Std Mean m/z	Std m/z Diff
353.1724											
	1	C 20 H 26 Na O 4	353.1723	-0.2	0.1	ok	even	16.50	0.0269	0.0004	0.0009

Bruker Compass DataAnalysis 4.0 printed: 6/7/2010 5:58:32 PM Page 1 of 1

Figure S52. IR spectrum of formosin F (**6**)

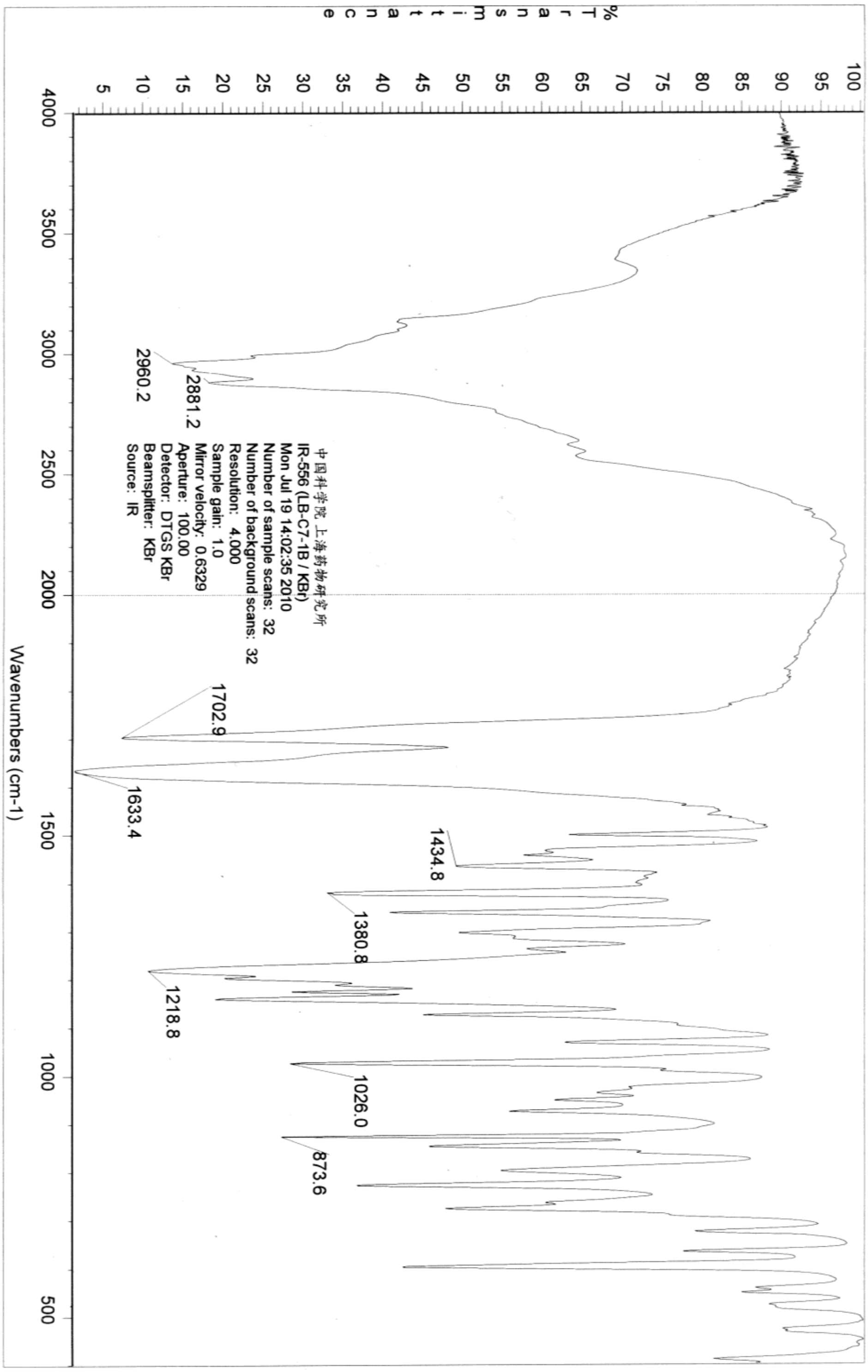

Supplement: Supplementary file 1 — Supplementary material 1 (PDF 3792 kb) Electronic supplementary material The online version of this article (doi:) contains supplementary material, which is available to authorized users [file 13659_2016_86_MOESM1_ESM.pdf]
